# Luminescent Platform for Thermal Sensing and Imaging Based on Structural Phase‐Transition

**DOI:** 10.1002/advs.202508920

**Published:** 2025-07-04

**Authors:** Anam Javaid, Maja Szymczak, Malgorzata Kubicka, Vasyl Kinzhybalo, Marek Drozd, Damian Szymanski, Lukasz Marciniak

**Affiliations:** ^1^ Institute of Low Temperature and Structure Research Polish Academy of Sciences Okólna 2 Wrocław 50‐422 Poland

**Keywords:** luminescence thermometry, no‐filter approach, phase transition, thermal imaging

## Abstract

The luminescent properties of Eu^3+^ ions are highly sensitive to changes in their local crystal environment. While this feature has been widely studied, its application in thermometers based on thermally induced structural phase transitions is a recent development. These thermometers often suffer from a narrow thermal operating range, prompting the search for new host materials. In this context, Na_3_Sc_2_(PO_4_)_3_:Eu^3+^ as a function of temperature and dopant ion concentration. As demonstrated, Na_3_Sc_2_(PO_4_)_3_:Eu^3+^ was investigated as a potential candidate. This material undergoes a reversible phase transition from a monoclinic to a trigonal structure, leading to significant changes in both the emission spectra and the luminescence decay of Eu^3+^ ions. These effects enable the development of both ratiometric and lifetime‐based luminescent thermometers, achieving maximum relative sensitivities of 3.4% K^−1^ and 1.0% K^−^1, respectively. Furthermore, the thermal operating range can be tuned by adjusting the Eu^3+^ concentration. Importantly, this study demonstrates, for the first time, temperature imaging using only Eu^3+^‐doped phosphor via a digital camera without the use of optical filters. These results position Na_3_Sc_2_(PO_4_)_3_:Eu^3+^ as a promising multifunctional material for advanced applications in contactless temperature sensing and thermal imaging.

## Graphical abstract

This study demonstrates the feasibility of employing a Eu3+‐doped, phase transition‐based ratiometric luminescent thermometer for thermal imaging application.

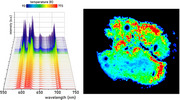

## 1 Introduction

Luminescent thermometry, a technique for temperature measurement based on changes in the spectroscopic properties of a phosphor, has seen significant advancement in recent years due to its unique capabilities.^[^ ^1^, ^2^, ^3^, ^4^, ^5^, ^6^, ^7^, ^8^ ^]^ Chief among these are remote, rapid, and dynamic readout.^[^ ^9^, ^10^ ^]^ Moreover, luminescent thermometry is one of the few techniques that enables not only point‐specific temperature readout but also thermal imaging of the analysed object.^[^ ^11^, ^12^, ^13^, ^14^ ^]^ Of the various spectroscopic parameters of the phosphor that can be utilized for temperature measurement, the ratiometric and lifetime‐based approaches offer the highest reading reliability. In these approaches, temperature is determined through the thermal change in either the luminescence intensity ratio or luminescence decay.^[^ ^3^, ^15^ ^]^ However, due to physical constraints, the thermal operating range of luminescent thermometers is limited to the range within which the luminescence intensity of one of the monitored bands remains detectable. Conversely, a dynamic thermal response in the measured parameter favours high relative sensitivity in luminescent thermometers.^[^ ^9^, ^10^, ^16^ ^]^ Considering these factors, two main directions have emerged in the development of luminescent thermometry: i) thermometers with relatively low sensitivity but a wide thermal operating range,^[^ ^17^, ^18^, ^19^, ^20^, ^21^ ^]^ and ii) thermometers with high sensitivity operating within a narrow thermal range.^[^ ^7^, ^16^, ^22^, ^23^, ^24^ ^]^ Although both of these directions are equally important, the choice of which of these types of luminescent thermometers should be used is determined by the requirements of a specific application. In many cases, the thermal operating range of a given system or device requiring temperature measurement is relatively not wide and in such a case high relative sensitivity is highly desirable.^[^ ^25^, ^26^, ^27^, ^28^, ^29^ ^]^

One recent approach to achieving high sensitivity within a narrow thermal range involves thermometers that utilize thermally induced structural phase transitions.^[^ ^30^, ^31^, ^32^, ^33^, ^34^, ^35^ ^]^ In this method, the structural phase transition alters the point symmetry of the crystallographic site occupied by the luminescent ion, resulting in a change in the emission spectrum shape and enabling a ratiometric approach to temperature measurement. An important advantage of this approach, aside from high relative sensitivity, is the ability to adjust the operating range by altering the host material composition to suit application requirements.^[^ ^32^, ^33^ ^]^ However, this tunning is feasible only within a certain thermal range.

Therefore, further research on the implementation of other host materials into phase‐transition‐based luminescence thermometers is highly desirable. In this work, we present a study on the impact of the thermally induced structural transition in Na_3_Sc_2_(PO_4_)_3_ on the spectroscopic properties of doped Eu^3+^ ions. As is well known, in Na_3_Sc_2_(PO_4_)_3_, temperature changes lead to two structural phase transitions, a feature highly relevant for luminescent thermometry.^[^ ^36^, ^37^, ^38^, ^39^, ^40^, ^41^, ^42^, ^43^, ^44^, ^45^, ^46^, ^47^ ^]^ The observed thermal variations in the spectroscopic properties of Na_3_Sc_2_(PO_4_)_3_:Eu^3+^, manifesting as changes in the emission spectrum shape and depopulation kinetics of the **^5^D_0_** level of Eu^3+^ ions, were examined as a function of Eu^3+^ ion concentration. These studies demonstrate the potential of Na_3_Sc_2_(PO_4_)_3_:Eu^3+^ for use in both ratiometric and lifetime‐based luminescent thermometry.

The luminescence of Eu^3+^ doped phosphors is usually not considered for temperature sensing application. This is largely due to the fact that the two most intense emission bands of Eu^3+^, localized ≈590 nm (^5^D_0_→^7^F_1_) and 620 nm (^5^D_0_→^7^F_2_), both arise from the depopulation of the same ^5^D_0_ excited state. As a result, conventional luminescence thermal quenching mechanisms lead to a comparable reduction in intensity for both transitions, limiting their utility for differential thermal analysis. The main novelty of this work is associated with the approach which leverages a thermally induced structural phase transition in the host matrix, which alters the local symmetry of the crystallographic site occupied by Eu^3+^ ions. This change preferentially affects the electric dipole transition (^5^D_0_→^7^F_2_), which is highly sensitive to the local environment, while the magnetic dipole transition (^5^D_0_→^7^F_1_) remains comparatively stable. This differential response enables ratiometric analysis based on the relative intensities of the two transitions. Furthermore, we report for the first time that the distinct spectral features of these transitions allow for their separate detection via the green and red channels of a standard digital camera. This capability facilitates filter‐free thermal imaging using the developed phosphor, a result we have confirmed experimentally.

## 2 Results and Discussion

**Figure 1:**
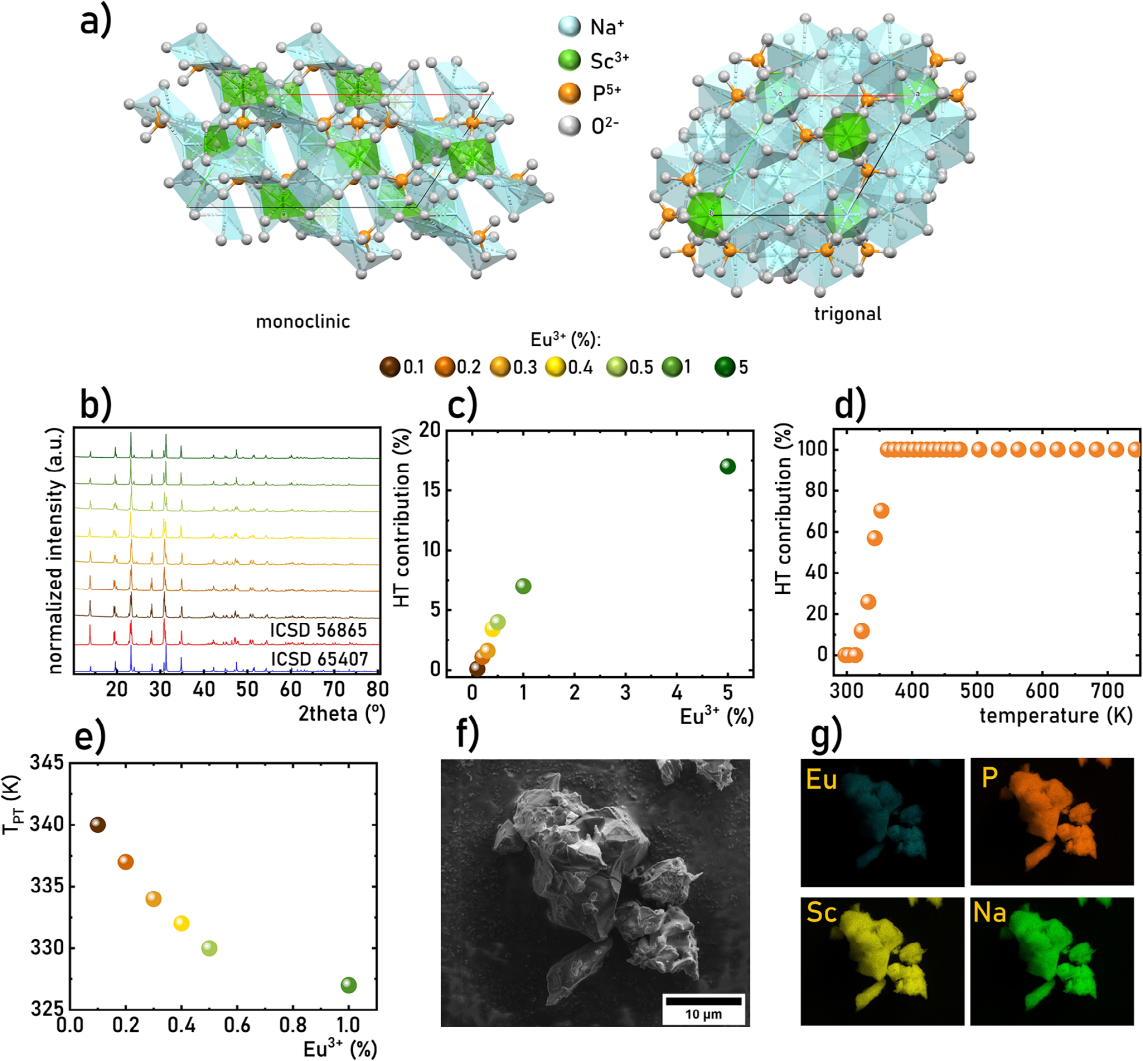
Visualization of the structure of the monoclinic and trigonal phases of Na_3_Sc_2_(PO_4_)_3_ a) the comparison of room temperature XRD patterns of Na_3_Sc_2_(PO_4_)_3_:Eu^3+^ with different Eu^3+^ concentration b); the contribution of the trigonal phase of Na_3_Sc_2_(PO_4_)_3_ in the room temperature XRD patterns as a function of Eu^3+^ concentration c); the contribution of the trigonal phase of Na_3_Sc_2_(PO_4_)_3_ as a function of temperature for Na_3_Sc_2_(PO_4_)_3_:0.2%Eu^3+^ d); temperature of the *α*→*β* phase transition of Na_3_Sc_2_(PO_4_)_3_:Eu^3+^ as a function of Eu^3+^ concentration determined from the DSC analysis e); representative SEM image f) and corresponding elemental maps of the Eu (cyan), P (orange), Sc (yellow) and Na (green) for Na_3_Sc_2_(PO_4_)_3_:0.2%Eu^3+^.

It is widely reported that the Na_3_Sc_2_(PO_4_)_3_ exists in three crystal structures(*α*, *β*, and *γ* phases), depending on the synthesis conditions and ionic substitution schemes.^[^ ^36^, ^37^, ^38^, ^39^, ^40^, ^41^, ^42^, ^43^, ^44^, ^45^, ^48^ ^]^ The *α* phase is a monoclinic structure with a space group *Bb*, while the *β* and *γ* phases have a trigonal structure with *R3c* space group (**Figure** **1**a).^[^ ^49^ ^]^ All three phases possess a structure composed of ScO_6_ octahedra, PO_4_ tetrahedra and 5, 7, and 8‐coordinated by O^2‐^ Na^+^ ions. For both phases, the most preferential location for Eu^3+^ dopant ions is the six‐fold coordinated Sc^3+^ ions, due to the similarity in ionic radius as well as the same ionic charge. The room temperature XRD patterns of Na_3_Sc_2_(PO_4_)_3_ with different concentration of Eu^3+^ correlate well with the reference patterns Figure 1b, see also Figure S1, Supporting Information).^[^ ^49^ ^]^ However, the Rietveld refinement of the obtained XRD patterns revealed small (≈1.2%) amount of ScPO_4_ (Figures S2–S30, Supporting Information). Due to the its small amount and the fact that ScPO_4_ does not undergo of thermally induced phase transition in the analyzed thermal range its presence does not have influence on the optical properties investigated in this paper.

As reported in the literature, the Na_3_Sc_2_(PO_4_) undergoes two first‐order reversible, temperature‐induced phase transitions: *α* ↔ *β* at T ≈313–337 K^[^ ^48^ ^]^ and *β* ↔ *γ* at ≈439 K^[^ ^50^ ^]^ (during heating). However, any evidence of *β* ↔ *γ* phase transition was recognized based on the XRD patterns analysis. The analysis of the XRD pattern of Na_3_Sc_2_(PO_4_):0.2%Eu^3+^ measured as a function of temperature indicates that at room temperature sample consist almost only of the low temperature *α* phase. An increase in temperature results in gradual increase of the amount of high‐temperature *β* phase and ≈350 K its contribution reached 50%. Above 380 K only *β* phase was observed. Due to the smaller ionic radii of Eu^3+^ ions in respect to the host Sc^3+^ cations an increase in the doping level results in a slight decrease of the phase transition temperature. This effect associated with the structure strain induced by the doping of host material was frequently reported in the literature and can be used for the modulation of the phase transition temperature.^[^ ^32^, ^35^, ^51^, ^52^ ^]^ The difference in ionic radii between host material cations and dopant ions causes the interionic distances in the host material to change thus modifying the value of the thermal energy that must be supplied to induce the phase transition. According to previous studies performed on LiYO_2_:Eu^3+^,Yb^3+^, Gd^3+^, when a cation is substituted whose place is occupied by an Eu^3+^ ion with an ion with a larger ionic radius than the host cation, the temperature of the phase transition decreases while the opposite effect is observed for a dopant cation with a smaller ionic radius.^[^ ^32^ ^]^ Therefore, the contribution of the *β* phase enhances in room temperature XRD patterns with an increase in Eu^3+^ concentration (Figure 1d). The highest value reaching 16.8% was obtained for Na_3_Sc_2_(PO_4_):5%Eu^3+^. To trance the influence of Eu^3+^ dopant concentration on *β* ↔ *γ* phase transition temperature the DSC analysis was performed (Figure 1e). The obtained results revealed that the phase transition temperature monotonically decreases from 340 K for 0.1%Eu^3+^ to 326 K for 5%Eu^3+^. Morphological studies confirm that the synthesized powders consist of microsized grains (Figure 1f, see also Figure S31, Supporting Information) with the uniformly distributed elements (Figure 1f, see also Figure S32, Supporting Information for O distribution). The calculation of the molar proportion between constituent elements confirmed correlates well with the intentional composition of the phosphor (see Table S1 and Figure S33, Supporting Information).

**Figure 2:**
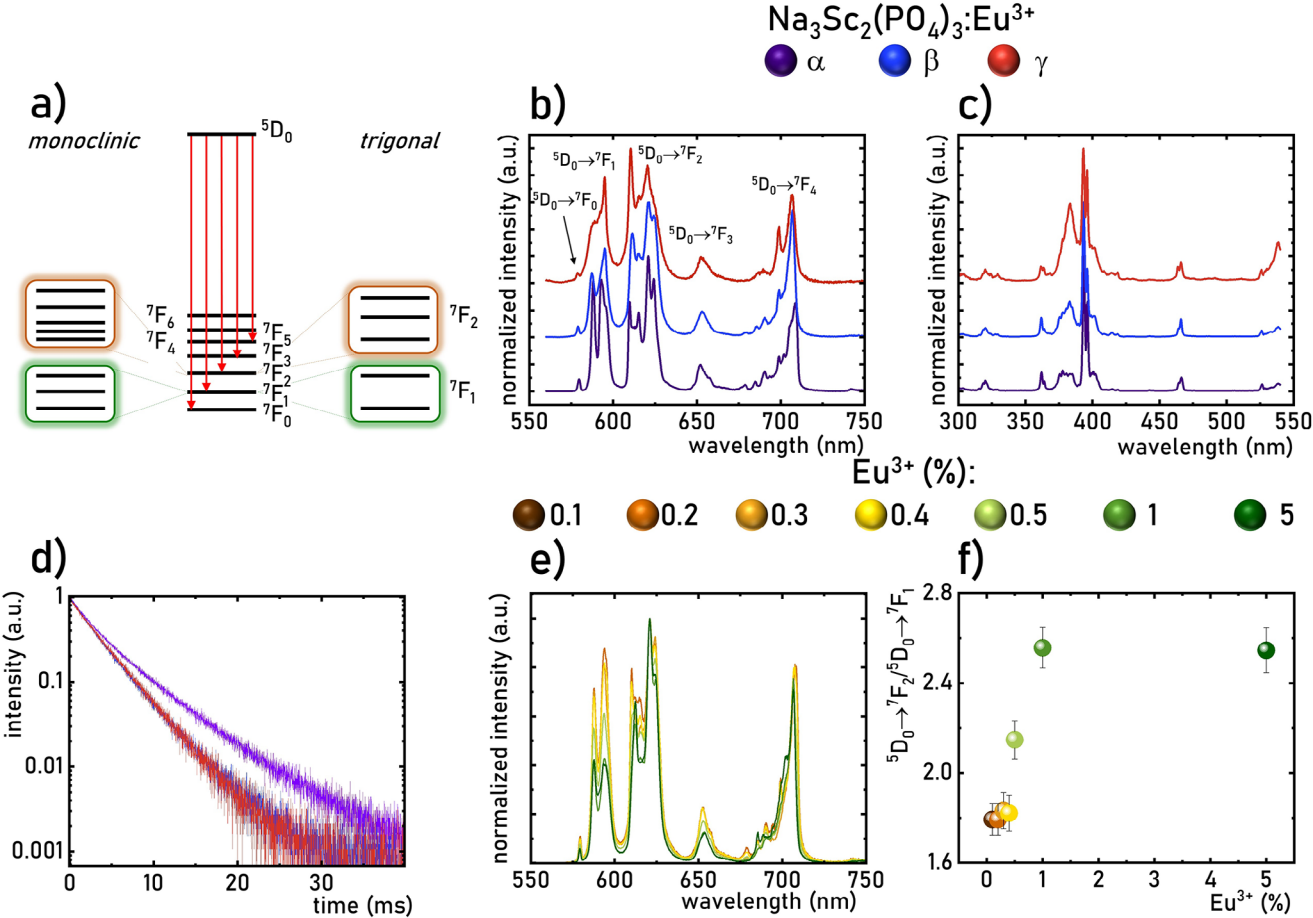
Simplified energy level diagram of Eu^3+^ ions a); comparison of the emission spectra of Na_3_Sc_2_(PO_4_)_3_: 0.2%Eu^3+^ measured at 83 K (violet line) 363 K (blue line) and 603 K (red line) b) and corresponding excitation spectra (*λ_exc_* = 393.5 nm) c); and luminescence decay profiles *(λ_exc_* = 393.5 nm, *λ_em_* = 620 nm) d); the comparison of the normalized room temperature emission spectra of Na_3_Sc_2_(PO_4_)_3_: Eu^3+^ with different concentration of dopant ions e) and the asymmetric ratio as a function of Eu^3+^ concentration f).

The luminescence properties of Eu^3+^ ions are associated with the intraconfigurational *4f→4f* electronic transitions.^[^ ^53^, ^54^, ^55^, ^56^ ^]^ The shape of the emission spectra is primarily influenced by the radiative depopulation of the 5D_0_ excited state to the 4F_J_ multiplets. Eu^3^⁺ ions stand out among lanthanides ions due to the high sensitivity of their luminescent properties to changes in the structural environment of the crystallographic sites they occupy within the host material.^[^ ^57^, ^58^, ^59^ ^]^ This sensitivity manifests in several ways. First, the 5D_0_→7F_1_ electronic transition is a magnetic dipole transition, whereas the 5D_0_→7F_2_ transition is of electric dipole character.^[^ ^59^ ^]^ Unlike magnetic dipole, the intensity of electric dipole transitions depends on the point symmetry of the ion's environment and in many cases increases as the symmetry decreases. Therefore, the intensity ratio of the 5D_0_→7F_2_ to 5D_0_→7F_1_ transitions, often termed the asymmetry ratio, is sometimes used as a measure of point symmetry.^[^ ^60^, ^61^, ^62^, ^63^ ^]^ However, as noted by Binneman,^[^ ^60^ ^]^ the intensity of the 5D_0_→7F_2_ band can be influenced by multiple factors, making it misleading to directly correlate an increase in asymmetry ratio with a decrease in point symmetry. Nonetheless, asymmetry ratio analysis remains a useful indicator of structural change. Furthermore, since the 5D_0_ and 7F_0_ levels do not split into Stark components, the 5D_0_→7F_0_ electronic transition, observed in low‐symmetry host materials, appears as a single emission line. The presence of multiple lines for this transition indicates emissions from non‐equivalent crystallographic sites occupied by Eu^3+^ ions. Additionally, the number of Stark components into which the Eu^3+^ multiplets split depends on the host material's symmetry, with higher symmetry resulting in fewer Stark levels.^[^ ^60^ ^]^ Therefore, for instance, in a monoclinic phase of Na_3_Sc_2_(PO_4_)_3_:Eu^3+^, the 7F_1_ and 7F_2_ levels split into three and five Stark levels, respectively (**Figure** **2**a), whereas, in trigonal symmetry, these multiplets split into two and three Stark levels, respectively. Due to these properties, Eu^3+^ ions, often called ‘optical structural probes’,^[^ ^60^, ^61^, ^62^, ^63^, ^64^ ^]^ are excellent candidates for analyzing structural changes in Na_3_Sc_2_(PO_4_)_3_. Emission spectra for Na_3_Sc_2_(PO_4_)_3_:Eu^3+^, measured at three different temperatures (83 K, 373 K, 500 K) based on DSC analysis, represent *α*, *β* and *γ* phases of Na_3_Sc_2_(PO_4_)_3_:Eu^3+^, respectively (Figure 2b). In each analyzed phase, a single line corresponding to the 5D_0_→7F_0_ transition is observed, indicating the emission corresponds to a single type of Eu^3^⁺ luminescent center. Both the shape of the Eu^3^⁺ emission bands and their relative intensities significantly depend on the crystallographic phase. For the monoclinic phase of Na_3_Sc_2_(PO_4_)_3_:Eu^3+^, the emission bands display more Stark lines than in the trigonal phases (Figure 2b). Additionally, the intensity of the 5D_0_→7F_2_ band increases in respect to the 5D_0_→7F_1_ band as the structure changes from *α* to *β*. The obtained LIR parameter values for these phases are 1.62 for *α*, 2.34 for *β*, and 2.3 for *γ*. Although less pronounced, differences between different Na_3_Sc_2_(PO_4_)_3_:Eu^3+^ phases are also observed in the excitation spectra (Figure 2c, see also Figure S34, Supporting Information). Regardless of the crystallographic phase, however, the excitation spectra are dominated by the 7F_0_→5L_6_ band at ≈393.5 nm, which was used as the excitation wavelength throughout this study. A comparison of luminescence decay profiles for all Na_3_Sc_2_(PO_4_)_3_:Eu^3+^ phases indicates that the longest *τ_avr_* = 3.37 ms was obtained in phase *α* (Figure 2d). The phase transition to *β* shortened *τ_avr_* to 2.47 ms, while the *β*‐to‐*γ* transition did not alter the luminescence kinetics of Eu^3+^ ions. Given that the 5D_0_ emitting level is separated from the 7F_6_ level by ≈11 500 cm^−1^, the probability of its nonradiative depopulation via multiphonon processes is low.^[^ ^65^, ^66^, ^67^, ^68^, ^69^ ^]^ According to the studies performed by Kim et al, the maximal phonon energy in this system is ≈1200 cm^−1^.^[^ ^70^ ^]^ Thus, the observed *τ_avr_* reduction can be attributed to an increased probability of radiative depopulation of the 5D_0_ state associated with the structural phase transition.^[^ ^32^, ^71^ ^]^ This hypothesis is in agreement with the higher emission intensity of the emission band attributed to the ^5^D_0_→^7^F_2_ electronic transition observed for β in respect to the α.

These results clearly show that both the emission spectra and depopulation kinetics of the 5D_0_ level of Eu^3+^ ions are sensitive to thermally induced structural transitions in Na_3_Sc_2_(PO_4_)_3_:Eu^3+^. As demonstrated by DSC studies, increasing the Eu^3+^ ion concentration lowers the structural phase transition temperature in Na_3_Sc_2_(PO_4_)_3_:Eu^3+^, consequently impacting the shape of the obtained room temperature emission spectra. As the Eu^3+^ concentration increases, the intensity of the 5D_0_→7F_2_ band rises in respect to the 5D_0_→7F_1_ band (Figure 2e). The ratio of these intensities increases monotonically to a value of 2.59 for 1%Eu^3+^, beyond which further doping does not alter this parameter (Figure 2f). This enhancement in the luminescence intensity ratio (LIR) for low Eu^3+^ concentrations is attributed to an increase in the high‐temperature phase of Na_3_Sc_2_(PO_4_)_3_:Eu^3+^ and thus an increased presence of Eu^3+^ ions in the trigonal phase. However, LIR values at Eu^3+^ concentrations above 1% exceed those recorded for Na_3_Sc_2_(PO_4_)_3_:0.2%Eu^3+^, suggesting that effects beyond phase transition influence the material at high dopant concentrations. These additional effects likely stem from additional symmetry changes due to the reduction of the elemental cell of Na_3_Sc_2_(PO_4_)_3_:Eu^3+^ associated with the ionic radius difference between the Eu^3+^ dopant and the Sc^3+^ cation it replaces.^[^ ^72^ ^]^

**Figure 3:**
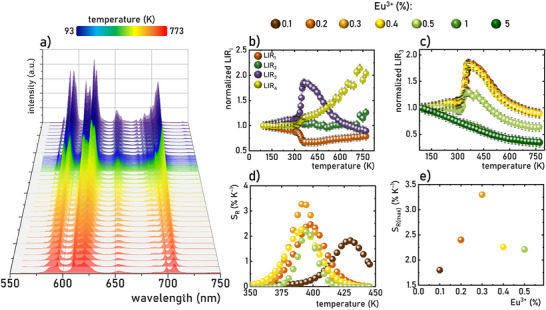
Luminescence spectra of Na_3_Sc_2_(PO_4_)_3_:0.2%Eu^3+^ measured as a function of temperature a); the thermal dependence of normalized *LIR_i_* for Na_3_Sc_2_(PO_4_)_3_:0.2%Eu^3+^ b); the thermal dependence of normalized *LIR_3_* for different Eu^3+^ ions concentrations c) and corresponding thermal dependence of *S_R_* d); the influence of the Eu^3+^ ions concentrations on the *S_Rmax_* of the ratiometric luminescence thermometers based on Na_3_Sc_2_(PO_4_)_3_:0.2%Eu^3+^ e).

As demonstrated above, the structural phase transition in Na_3_Sc_2_(PO_4_)_3_:Eu^3+^ significantly affects the shape of the emission spectra of Eu^3+^ ions (see also Figures S35–S40, Supporting Information). More pronounced changes are expected during the *α*–*β* transition due to the significant symmetry alteration in the structure. The analysis of the emission spectra of Na_3_Sc_2_(PO_4_)_3_:Eu^3+^ measured as a function of temperature reveals clear and gradual changes in spectral shape (**Figure** **3**a). Increasing the temperature results in a progressive reduction in the intensity of Stark lines associated with the luminescence of Eu^3+^ ions in the monoclinic phase, accompanied by an enhancement in those associated with the trigonal phase. These transitions alter the shape of each emission band as well as their relative intensities. To quantify these changes, four different luminescence intensity ratio (*LIR*) parameters were proposed as follows:

(1)

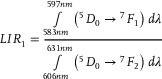



(2)

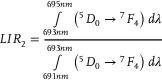



(3)

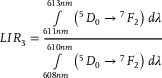



(4)

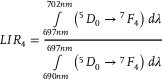




A comparison of the thermal dependence of *LIR_i_* for a representative sample of Na_3_Sc_2_(PO_4_)_3_:0.2%Eu^3+^, shown in Figure 3b, indicates significant changes in each *LIR* parameter with increasing temperature near the structural phase transition (see also Figures S41–S44, Supporting Information). However, the most pronounced variations are observed for *LIR_3_*, and thus the subsequent analysis in this work focuses on this thermometric parameter. To investigate the effect of Eu^3+^ ion concentration on the thermometric performance of the ratiometric luminescence thermometer of Na_3_Sc_2_(PO_4_)_3_:Eu^3+^, the thermal dependences of *LIR_3_* are presented in a normalized form (normalized to its initial value obtained at 83 K) (Figure 3c). This analysis reveals several noteworthy trends. For Eu^3^⁺ ion concentrations below 0.5%, *LIR_3_* initially increases slightly with rising temperature up to ≈300 K, above which exhibits a rapid increase, peaking at ≈370 K. Beyond this temperature, a further increase results in a gradual decline in *LIR_3_*. The threshold temperature at which a sharp rise in *LIR_3_* is observed decreases with increasing Eu^3+^ ion concentration, consistent with the lowering of the phase transition temperature. For a concentration of 0.3%Eu^3+^, a nearly twofold increase in *LIR_3_* values is observed in the 300–380 K thermal range. Conversely, for Eu^3+^ concentrations exceeding 1%, *LIR_3_* exhibits a monotonic decrease across the entire temperature range analyzed. It is critical to note that for reliable temperature sensing with a luminescent thermometer, the temperature‐dependent parameter must exhibit a monotonic trend within the operational thermal range. Consequently, the decrease in *LIR_3_* above ≈450 K renders Na_3_Sc_2_(PO_4_)_3_:Eu^3+^ unsuitable for temperature sensing beyond this threshold. Furthermore, the thermal operating range gradually narrows with increasing Eu^3+^ ion concentration. Quantitative evaluation of the observed changes in *LIR_3_* is achieved by determining the relative sensitivity (*S_R_*) using the following equation:

(5) $$S_{R}=\frac{1}{LIR}\frac{\Delta LIR}{\Delta T}\cdot 100\%$$

where *∆LIR* represents the change in *LIR* corresponding to the change in temperature by *∆T*. The *S_R_* values for Na_3_Sc_2_(PO_4_)_3_:Eu^3+^ gradually increase with temperature, reaching a maximum at ≈390 K (Figure 3d). However, the temperature at which *S_Rmax_* is observed decreases progressively with higher Eu^3+^ ion concentrations. The maximum *S_R_* value increases with rising dopant ion concentration, peaking at *S_Rmax_* = 3.4% K^−1^ for 0.3%Eu^3 +^, after which further increases in Eu^3+^ concentration result in a decline in *S_R_* (Figure 3e). However, the reason why the highest *S_R_* value was obtained for 0.3%Eu^3+^ is not entirely clear. Notably, all the *S_R_* values obtained exceed 1.7% K^−1^, a relatively high sensitivity compared to other ratiometric luminescence thermometers based on lanthanide ion emissions.^[^ ^6^, ^16^, ^73^ ^]^ It should be noted here that the ratiometric response of the luminescence thermometers based on Na_3_Sc_2_(PO_4_)_3_:0.3%Eu^3+^ is characterized by high repeatability confirmed within several heating‐cooling cycles (Figure S45a, Supporting Information). Additionally, when the Na_3_Sc_2_(PO_4_)_3_:0.3%Eu^3+^ was exposed on the constant temperature condition (365 K in this case) the response time (considered as a time after which LIR reached its final and constant value) was found to be ≈1s (Figure S45b, Supporting Information), which represents reasonable values for many different type of applications. Moreover, due to the high chemical stability of this inorganic phosphor the thermometric performance of this luminescence thermometer was not change within 4 weeks of measurements. The calibration curve for thermal dependence of LIR can be expressed using the equation shown in Equation S1, see also Table S2 (Supporting Information) for representative fitting parameters.

**Figure 4:**
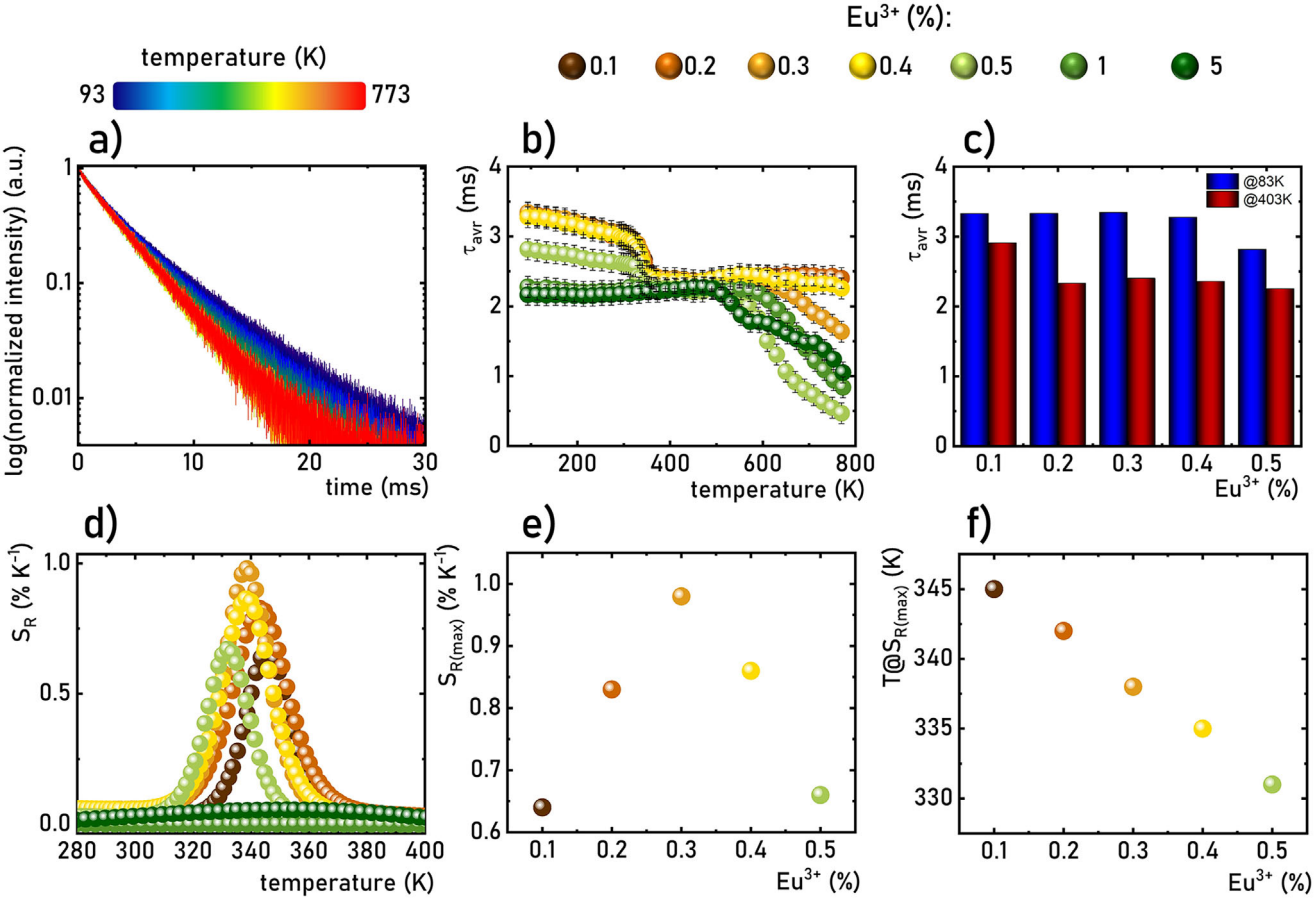
Luminescence decay profile of Na_3_Sc_2_(PO_4_)_3_:0.2%Eu^3+^ measured as a function of temperature a); *τ_avr_* as a function of temperature for different concentrations of Eu^3+^ ions b); comparison of the *τ_avr_* at 83 K and 403 K for different concentrations of Eu^3+^ ions c); thermal dependence of *S_R_* of lifetime‐based luminescence thermometers on Na_3_Sc_2_(PO_4_)_3_:Eu^3+^ with different concentration of Eu^3+^ ions d) *S_Rmax_* as a function of dopant concentration e) and influence of Eu^3+^ concentration on a temperature at which *S_Rmax_* was obtained f).

As discussed above, structural changes associated with the thermally induced phase transition in Na_3_Sc_2_(PO_4_)_3_:Eu^3+^ result in notable variations in luminescence kinetics of Eu^3+^ ions (Figures S46–S51, Supporting Information). The analysis of luminescence decay profiles of Eu^3+^ ions in Na_3_Sc_2_(PO_4_)_3_:Eu^3+^ yields several intriguing observations. As the temperature increases beyond 83 K, initially minor changes in the lifetimes are observed (**Figure** **4**a). However, beyond a certain threshold temperature, a pronounced shortening of the *τ_avr_* becomes evident. This trend is clearly reflected in the thermal dependence of *τ_avr_* (Figure 4b). For Eu^3+^ ion concentrations below 0.4%, a *τ_avr_* of 3.30 ms is observed, and increasing the temperature does not significantly affect its value until ≈330 K, corresponding to the *α*→*β* phase transition. In the temperature range associated with this phase transition, a sharp reduction in *τ_avr_* to ≈2.5 ms is observed, above which further increases in temperature do not result in significant changes. For a concentration of 0.5% Eu^3+^, a similar trend is noted, though the initial lifetime value at 83 K is lower (*τ_avr_* = 2.81 ms), and further thermal shortening of the lifetime becomes apparent above ≈550 K. At higher dopant concentrations, the *τ_avr_* exhibit minimal thermal variation until ≈500 K, beyond which its thermal shortening is observed. It is worth to underline that for 0.1‐0.4%Eu^3+^ dopant concentrations, the temperature at which *τ_avr_* begins to decrease shifts to lower values as the concentration of Eu^3+^ increases, reflecting a corresponding reduction in the phase transition temperature. A comparison of *τ_avr_* values at 83 K indicates that below 0.4%Eu^3+^, *τ_avr_* remains independent of dopant ion concentration (Figure 4c). This confirms the absence of interionic interactions between dopants, consistent with the energy level diagram of Eu^3+^ ions. The reduction in *τ_avr_* observed for 0.5%Eu^3+^ may suggest additional structural changes related to the difference in ionic radii between the Eu^3+^ dopant ions and the host cations, as mentioned earlier. Interestingly, the *τ_avr_* values after the *α*→*β* phase transition are nearly independent of dopant ion concentration.

The relative sensitivity for a lifetime‐based luminescence thermometer can be calculated analogously to Equation (5) (substituting *LIR* with *τ_avr_*). The maximum *S_R_* values are observed at temperatures corresponding to the structural phase transition (Figure 4d). However, the absence of *τ_avr_* shortening for Eu^3+^ ion concentrations of 1% and 5% near 340 K results in negligible *S_R_* values in this range. The highest *S_R_* value was recorded for 0.3%Eu^3+^, with *S_R_* = 1.0% K^−1^, and this value decreases as the Eu^3+^ ion concentration changes (Figure 4e). Notably, a linear correlation was observed between the temperature at which *S_Rmax_* occurs and the increasing dopant ion concentration (Figure 4f). This result provides direct confirmation of the potential for fine‐tuning the thermometric performance of the luminescent thermometer by adjusting the dopant ion concentration. Such tunability is highly significant in meeting the requirements of various potential applications.

**Figure 5:**
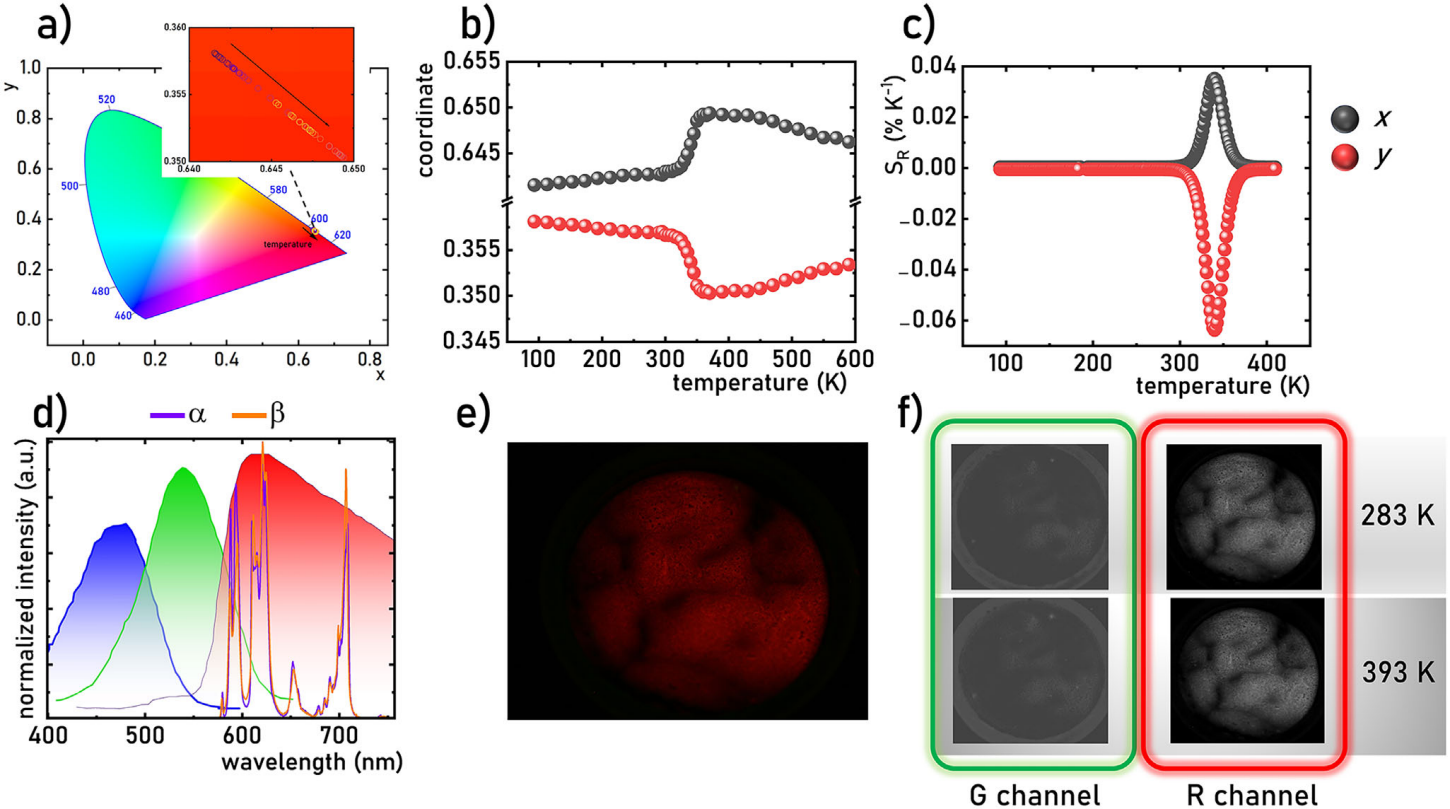
The influence of temperature on the CIE 1931 chromatic coordinates for Na_3_Sc_2_(PO_4_)_3_:0.2%Eu^3+^ a); thermal dependence of *x* (black dots) and *y* (red dots) chromatic coordinates b) and corresponding relative sensitivities c); the comparison of normalized emission spectra of low temperature (violet line) and high temperature (orange line) phase of the Na_3_Sc_2_(PO_4_)_3_:0.2%Eu^3+^ with the spectra of sensitivities of RGB channels of digital camera d); representative image of luminescence of Na_3_Sc_2_(PO_4_)_3_:0.2%Eu^3+^ measured at 283 K e) and the 2D maps of luminescence intensities registered in G and R channels for Na_3_Sc_2_(PO_4_)_3_:0.2%Eu^3+^ at 283 K and 393 K f).

As shown above, the changes induced by the structural phase transition in the local environment of Eu^3+^ ions in Na_3_Sc_2_(PO_4_)_3_:Eu^3+^ result in a change in the intensity ratio of the ^5^D_0_→^7^F_2_ to ^5^D_0_→^7^F_1_ emission bands. Since the former band falls within the red region of the spectrum and the latter within the yellow region, relative changes in their intensities lead to a variation in the color of the emitted light. However, as shown in the CIE 1931 chromaticity diagram (**Figure** **5**a), this change in color is relatively small. A detailed analysis of the chromaticity coordinates of the emitted light from Na_3_Sc_2_(PO_4_)_3_:0.2%Eu^3+^ as a function of temperature reveals that, near the phase transition temperature, *x* increases from 0.6416 to 0.6493, while *y* decreases from 0.35828 to 0.35032 (Figure 5b). These shifts enable temperature readout based on the emitted light's color. Nevertheless, the relative sensitivities calculated from the chromatic coordinates yield modest values (for *x*, *S_Rmax_* = 0.036 % K^−1^; for *y*, *S_Rmax_* = −0.064457 % K^−1^) (Figure 5c). The negative values of the *S_R_* in the case of *y* coordinate results from thermal reduction of *y* value. On the other hand, a comparison of the Eu^3+^ ion emission spectra for the low‐temperature (LT) and high‐temperature (HT) phases of Na_3_Sc_2_(PO_4_)_3_:Eu^3+^ with the spectral sensitivity ranges of RGB channels of a conventional digital camera reveals that while the R channel captures intensity contributions from both the ^5^D_0_→^7^F_2_ and ^5^D_0_→^7^F_1_ bands, the G channel is primarily sensitive to the ^5^D_0_→^7^F_1_ (Figure 5d). When an image is captured using a digital camera, the sensor simultaneously collects intensity data from all RGB channels. Based on this principle, we propose using the phase transition‐induced changes in the emission spectra of Na_3_Sc_2_(PO_4_)_3_:0.2%Eu^3+^ for temperature measurement and 2D imaging. To demonstrate this, a series of photographs of Na_3_Sc_2_(PO_4_)_3_:0.2%Eu^3+^ powder placed on a temperature‐controlled heating‐cooling stage under continuous optical excitation were taken (Figure 5e; Figure S52, Supporting Information). From the captured images, intensity maps for the R and G channels were extracted and analyzed (Figure 5f). Due to the limited sensitivity of the G channel in the spectral range corresponding to the ^5^D_0_→^7^F_1_ transition, the G channel displayed relatively low intensity values (intensity increases 20 times in Figure 5f, see also Figure S53, Supporting Information). These intensity maps were subsequently divided to calculate the R/G intensity ratio (Figure S54, Supporting Information). As expected, the R/G intensity ratio decreased with increasing temperature, directly validating the effectiveness of the proposed approach.

**Figure 6:**
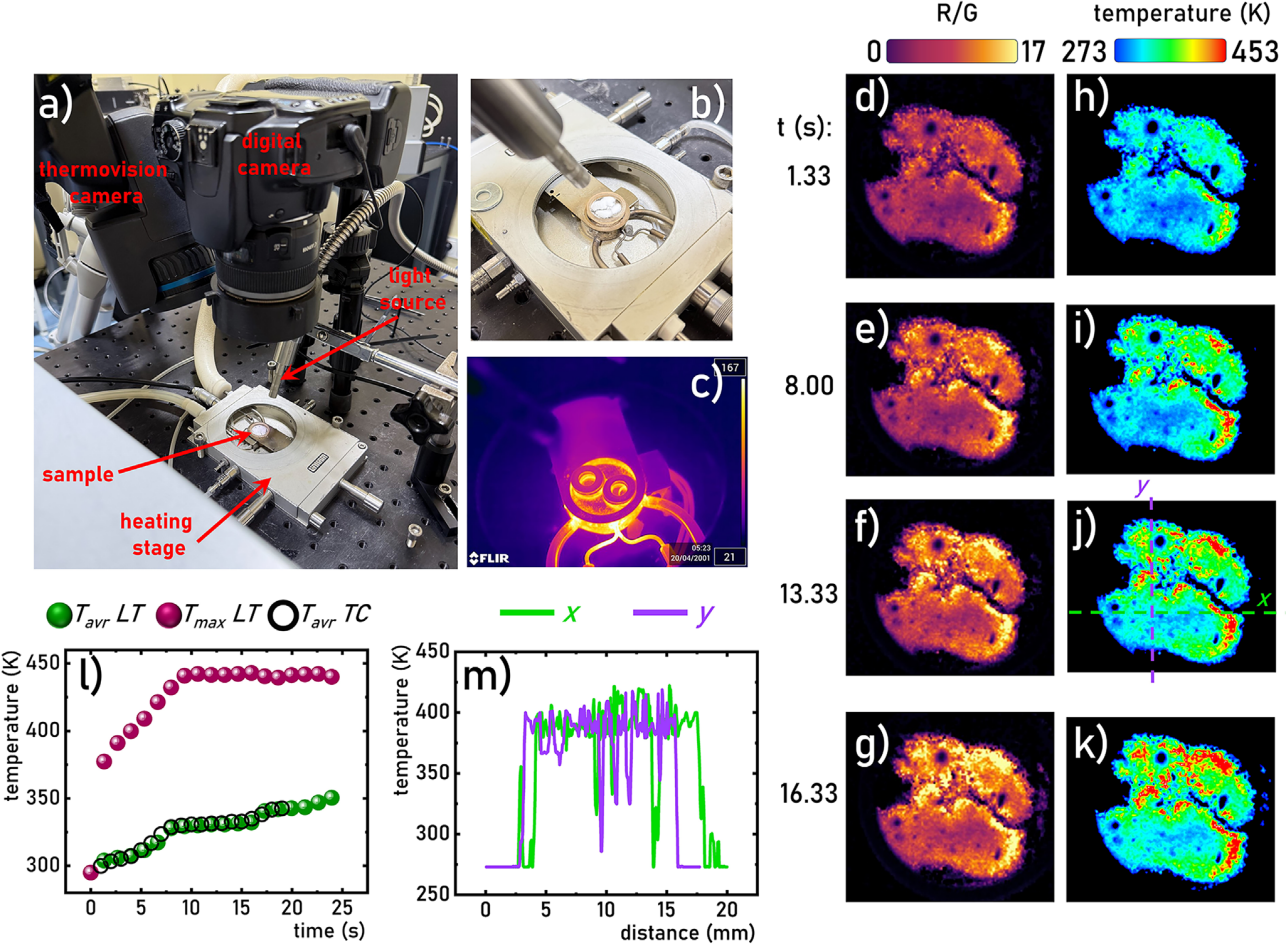
Photo of the experimental setup used for thermal imaging based on the optical response of the Na_3_Sc_2_(PO_4_)_3_:0.2%Eu^3+^ a); Na_3_Sc_2_(PO_4_)_3_:0.2%Eu^3+^ sample placed on the heating stage b) photo of the heating elements recorded using thermovision camera c); 2D maps of the R/G ratio obtained from the digital camera recorded as a function of time d–g); and corresponding thermal maps h–k); temporal dependence of the maximal (*T_max_*) and average (*T_avr_*) temperature of the phosphor determined using Na_3_Sc_2_(PO_4_)_3_:0.2%Eu^3+^ luminescence thermometer (*LT*) and thermovision camera (*TC*) l); temperature determined using luminescence thermometry across *x* and *y* axis marked in Figure 6j–m).

Given that the structural phase transition in Na_3_Sc_2_(PO_4_)_3_:0.2%Eu^3+^ induces a modification in the ratio of the emission intensities collected in the red and green channels of the digital camera, this phenomenon can be effectively exploited not only for temperature spot‐readout but also for spatially resolved temperature imaging. The uniqueness of this effect lies in the fact that, under typical conditions, each of the most intense emission bands of Eu^3+^ ions (^5^D_0_→^7^F_1_ and ^5^D_0_→^7^F_2_) originates from the same emitting level (^5^D_0_). Therefore, significant temperature‐dependent changes in the intensity ratio of these bands are generally not expected. The observation of such a dependence in the case of Na_3_Sc_2_(PO_4_)_3_:0.2%Eu^3+^ is thus particularly noteworthy. To evaluate the potential of this phenomenon for temperature imaging, an experimental setup was designed in which the Na_3_Sc_2_(PO_4_)_3_ phosphor powder was spread on a quartz substrate (**Figure** **6**a,b), which was then placed on a heating stage maintained at a constant temperature of 453 K. To inhibit direct heat transfer and induce a thermal gradient, the quartz holder was thermally insulated from the heating stage using steel rings with a diameter of 5 mm, as illustrated in Figure 6b. After confirming thermal stabilization of the heating stage using thermovision camera (Figure 6c), the sample was positioned on the stage and time‐sequenced digital images were captured at intervals of 1.33 s. From each captured image, the red and green channels were extracted, and their pixel‐wise ratio (R/G) was calculated to generate 2D R/G distribution maps (Figure 6d–f). Using a previously established calibration curve, obtained under controlled isothermal conditions (Figure S54, Supporting Information), the R/G ratio maps were converted into corresponding temperature maps. The results, presented in Figure 6h–k (see also Figure S56, Supporting Information), clearly illustrate the development of a temperature gradient across the Na_3_Sc_2_(PO_4_)_3_:0.2%Eu^3+^ sample, with an observable increase in maximum temperature over time. The temporal evolution of temperature derived from the luminescence maps shows strong agreement with values obtained from the thermal imaging camera, with a maximum deviation of just 0.5 K (Figure 6l; Figure S57, Supporting Information). Notably, the luminescence‐based thermal maps achieved a spatial resolution of 0.2 mm and allowed for detailed temperature profiling along arbitrary cross‐sections of the sample (Figure 6m). While the temperature accuracy of the luminescence‐based measurements is in the same order of magnitude to that of conventional thermovision camera, a significant advantage of the luminescence thermometry approach is its ability to image temperature through transparent media in the visible spectral range. This was demonstrated by placing a quartz lid with an optical window over the sample (Figure S57, Supporting Information). In this configuration, the thermal imaging camera could only detect the surface temperature of the quartz window, whereas luminescence thermometry successfully captured the underlying temperature gradient of the phosphor. Moreover, luminescence thermometry offers spatial specificity: the temperature information is derived exclusively from the region containing the luminescent material, allowing for selective temperature mapping of the target area. This stands in contrast to conventional infrared thermal imaging, which measures the temperature of any object within its optical path, potentially reducing specificity in complex or multilayered systems. It should also be noted that the spatial resolution of thermal imaging using a luminescent thermometer is governed primarily by two factors: the size of the phosphor crystallites and the resolution of the detection system. In this study, the crystallite size was determined to be below 5 µm; therefore, the spatial resolution is predominantly limited by the digital camera employed. In our setup, the achieved spatial resolution was ≈69 µm (14.3 lp mm^−1^ according to the standard USAF 1951). The thermal accuracy, defined as the variation in measured temperature values under isothermal conditions at 393 K, was determined to be 0.4 K. In comparison, the spatial resolution of conventional thermal imaging cameras is device‐dependent and can vary widely. For example, the temperature resolution of typical thermal cameras operating below 393 K is generally ≈2 K (as is the case with the FLIR T540 camera used in our experiments).

The topic of luminescent thermometry based on structural phase transitions has recently gained growing interest, as evidenced by an increasing number of publications exploring this approach (**Table** **1**).^[^ ^31^, ^32^, ^33^, ^34^, ^71^, ^74^, ^75^, ^76^, ^77^, ^78^ ^]^ However, the inherently localized nature of first‐order structural phase transitions limits the structural changes, and thus the associated spectroscopic modifications, to a relatively narrow temperature range. As a result, the temperature at which the maximum relative sensitivity is observed often defines the effective operating range of the luminescent thermometer. This limitation arises from the fact that, in most phase‐transition‐based systems, a monotonic change in the luminescence intensity ratio (*LIR*) is typically confined to a thermal window of ≈±50 K around the phase transition temperature. Therefore, although such systems can exhibit exceptionally high *S_R_* values, tailoring the thermometer to a specific application‐defined temperature range requires identifying host materials with appropriately tuned phase transition temperatures. To some extent, it is possible to shift the thermal operating range of such a thermometer by co‐doping the system with ionic radii varying to host material cation as in the case of LiYO_2_:Eu^3+^,Gd^3+^/Yb^3+^.^[^ ^32^ ^]^ However, usually a reduction in S_R_ accompanies such co‐doping. For instance, while LiYO_2_:Ln^3+^ materials are known for their high relative sensitivities, their operational ranges are generally centered near room temperature.^[^ ^31^, ^32^, ^33^, ^71^, ^75^, ^77^, ^78^ ^]^ To overcome this constraint, new host matrices such as LaGaO_3_^[^ ^71^ ^]^ and Na_3_Sc_2_(PO_4_)_2_^[^ ^74^ ^]^ have recently been proposed. Notably, in contrast to Na_3_Sc_2_(PO_4_)_2_:Yb^3+^, the Na_3_Sc_2_(PO_4_)_2_:Eu^3+^ system described in this work exhibits significantly higher relative sensitivity. Furthermore, this study presents, for the first time, the feasibility of utilizing a structural phase transition for 2D thermal imaging. This result not only underscores the practical potential of phase‐transition‐based luminescent thermometry but also demonstrates, crucially and for the first time, that thermometric systems relying solely on Eu^3+^ luminescence can be effectively applied for thermal imaging applications.

**Table 1:** Comparison of the thermometric performance of ratiometric luminescence thermometers based on the first‐order structural phase transition.

Thermometer	LT phase	HT phase	LIR	*S_Rmax_* [% K^−1^]	*T@S_Rmax_* [K]	Refs.
LiYO_2_:5%Yb^3+^	monoclinic	tetragonal	^2^F_5/2_→^2^F_7/2_ / ^2^F_5/2_→^2^F_7/2_	5.3	280	[76]
Na_3_Sc_2_(PO_4_)_3_:Yb^3+^	monoclinic	Trigonal	^2^F_5/2_→^2^F_7/2_ / ^2^F_5/2_→^2^F_7/2_	1.5	340	[74]
LiYO_2_:Pr^3+^	monoclinic	tetragonal	^3^P_0_→^3^H_4_ / ^1^D_2_→^3^H_4_	23.04	329	[78]
LiYO_2_:1%Eu^3+^	monoclinic	tetragonal	^5^D_0_→^7^F_2_ / ^5^D_0_→^7^F_2_	12.5	305	[34]
LiYO_2_:1%Eu^3+^,30%Yb^3+^	monoclinic	tetragonal	^5^D_0_→^7^F_2_ / ^5^D_0_→^7^F_2_	2.1	180	[32]
LiYO_2_:1%Eu^3+^,40%Gd^3+^	monoclinic	tetragonal	^5^D_0_→^7^F_2_ / ^5^D_0_→^7^F_2_	1.4	550	[32]
LiYO_2_:0.1%Nd^3+^,	monoclinic	tetragonal	^4^F_3/2_→^4^I_9/2_ / ^4^F_3/2_→^4^I_9/2_	7.9	291	[75]
LiYO_2_: 1%Er^3+^, 10%Yb^3+^	monoclinic	tetragonal	^4^S_3/2_→^4^I_15/2_ /^4^S_3/2_→^4^I_15/2_	2.5	240	[31]
LiYO_2_: Dy^3+^,	monoclinic	tetragonal	^4^F_9/2_ → ^6^H_13/2_ / ^4^F_9/2_ → ^6^H_13/2_	35.24	310	[51]
LaGaO_3_:0.1%Eu^3+^	orthorhombic	trigonal	^5^D_0_→^7^F_2_ / ^5^D_0_→^7^F_2_	5.4	456	[71]
Na_3_Sc_2_(PO_4_)_3_:Eu^3+^	monoclinic	trigonal	^5^D_0_→^7^F_2_ / ^5^D_0_→^7^F_2_	3.4	390	This work

## 3 Conclusion

In this study, the spectroscopic properties of Na_3_Sc_2_(PO_4_)_3_:Eu^3+^ were investigated as a function of temperature and Eu^3^⁺ ion concentration to develop a novel luminescent thermometer based on a structural phase transition. The results show that an increase in temperature above ≈350 K induces a phase transition in Na_3_Sc_2_(PO_4_)_3_:Eu^3+^ from a monoclinic to a trigonal structure. This transition significantly alters the point symmetry of the crystallographic site occupied by Eu^3^⁺ ions, thereby modifying their spectroscopic properties. The studies revealed that the monoclinic‐to‐trigonal phase transition reduces the number of Stark levels into which the ^4^F_J_ multiplets split, changes the luminescence intensity ratio of the ^5^D_0_→^7^F_2_ to ^5^D_0_→^7^F_1_ transitions of Eu^3+^ ions, and shortens the lifetime of the ^5^D_0_ level. These changes enabled the development of a luminescence thermometer using both ratiometric and lifetime‐based approaches. The highest sensitivities, 3.4% K^−1^ and 1.0% K^−1^, were achieved for ratiometric and lifetime‐based luminescence thermometers, respectively, in Na_3_Sc_2_(PO_4_)_3_:0.3%Eu^3+^. Additionally, it was shown that increasing the Eu^3+^ ion concentration to 0.5% resulted in a reduction of the phase transition temperature, allowing a corresponding decrease in the temperature at which *S_Rmax_* was observed. However, for Eu^3+^ concentrations above 1%, the monoclinic‐to‐trigonal phase transition was not observed, likely due to the stabilization of the trigonal phase of Na_3_Sc_2_(PO_4_)_3_:Eu^3+^ caused by the ionic radius mismatch between the host material and dopant cations. The developed thermometers exhibited relatively high relative sensitivity and a thermal operating range of ≈75 K. Moreover, for the first time, the applicative potential of first‐order phase transition‐based luminescence thermometers in thermal imaging was demonstrated here. These findings demonstrate that the synergy between the high sensitivity of Eu^3+^ spectroscopic properties and thermally induced structural phase transitions can be effectively utilized for temperature sensing and imaging.

## 4 Experimental Section

### Synthesis

A series of powder samples of Na_3_Sc_2_(PO_4_)_3_:x%Eu^3+^, (where x = 0.1, 0.2, 0.3, 0.4, 0.5, 1, 2, 5) were synthesized using a conventional high‐temperature solid‐state reaction technique. Na_2_CO_3_ (99.9% of purity, Alfa Aesar), Sc_2_O_3_ (99.9% of purity, Alfa Aesar), NH_4_H_2_PO_4_ (99.9% of purity, POL‐AURA), and Eu_2_O_3_ (99.999 % of purity, Stanford Materials Corporation)) were used as starting materials. The stoichiometric amounts of reagents were finely ground in an agate mortar with few drops of hexane and, then annealed in the alumina crucibles at 1573 K for 5 h (heating rate of 10 K min^−1^) in air. The final powders were cooled to room temperature and then ground again.

### Characterization

The obtained materials were examined using powder X‐ray diffraction technique. Powder diffraction data were obtained in Bragg–Brentano geometry  using a PANalytical X'Pert Pro diffractometer equipped with Oxford Cryosystems Phenix (low‐temperature measurements) attachment using Ni‐filtered Cu K*α* radiation (V = 40 kV, I = 30 mA). Diffraction patterns in 2θ range of 15°–90° were measured in cooling/heating sequence in the temperature range from 320 K to 80 K. ICSD database entries No. 56865 (LT phase) and 65407 (HT phase) were taken as initial models for the analysis of the obtained diffraction data.

Morphology and chemical composition of the studied samples were determined with a Field Emission Scanning Electron Microscope (FE‐SEM, FEI Nova NanoSEM 230) equipped with an energy‐dispersive X‐ray spectrometer (EDX, EDAX Apollo X Silicon Drift Correction) compatible with Genesis EDAX microanalysis Software. Before SEM imaging, the Na_3_Sc_2_(PO_4_)_3_:0.2%Eu^3+^ sample (as a representative sample in the entire study series) was dispersed in alcohol, and then a drop of suspension was placed in the carbon stub. Finally, SEM images were recorded with an accelerating voltage of 5.0 kV in a beam deceleration mode which improves imaging parameters such as resolution and contras as well as reduces contamination. In the case of EDS measurements the sample was scanned at 30 kV.

A differential scanning calorimetric (DSC) measurements were performed using Perkin–Elmer DSC 8000 calorimeter equipped with Controlled Liquid Nitrogen Accessory LN2 with a heating/cooling rate of 20 K min^−1^. The sample was sealed in the aluminum pan. The measurement was performed for the powder sample in the 100–800 K temperature range. The excitation and emission spectra were obtained using the FLS1000 Fluorescence Spectrometer from Edinburgh Instruments equipped with 450 W Xenon lamp and R928 photomultiplier tube from Hamamatsu. Luminescence decay profiles were also recorded using the FLS1000 equipped with 150 W µFlash lamp. The average lifetime (*τ_avr_*, Equation 6) of the excited levels was calculated based on fit of the luminescence decay profiles by double‐exponential function (Equation 7):

(6) $$\tau_{avr}=\frac{A_{1}{\tau_{1}}^{2}+A_{2}{\tau_{2}}^{2}}{A_{1}\tau_{1}+A_{2}\tau_{2}}$$

(7) $$I\left(t\right)=I_{0}+A_{1}\cdot \exp\left(-\frac{t}{\tau_{1}}\right)+A_{2}\cdot \exp\left(-\frac{t}{\tau_{2}}\right)$$

where *τ_1_* and *τ_2_* represent the luminescence decay parameters and *A_1_, A_2_* are the fitted amplitudes of the double‐exponential function. During the temperature‐dependent emission measurements, the temperature of the sample was controlled by a THMS600 heating–cooling stage from Linkam (0.1 K temperature stability and 0.1 K set point resolution).

The digital images were taken using a Canon EOS 400D camera with a EFS 60 mm macro lens using a 1 s integration time, 14.3 lp mm^−1^ spatial resolution and long pass 450 nm filter from Thorlabs. After capturing color images the emission maps for red and green channels (RGB) were extracted using IrfanView 64 4.51 software. The R and G intensity maps were divided by each other using ImageJ 1.8.0_172 software. This procedure was also used for phosphor placed at constant temperatures to create a calibration curve used for R/G maps into thermal maps conversion. Thermovision images were collected using a T540 camera from FLIR.

## Conflict of Interest

The authors declare no conflict of interest.

## Supporting information



Supporting Information

## Data Availability

The data that support the findings of this study are available from the corresponding author upon reasonable request.
