# Supplementary material for: Luminescent Platform for Thermal Sensing and Imaging Based on Structural Phase‐Transition
Source: Adv Sci (Weinh). 2025 Jul 4;12(37):e08920. doi: 10.1002/advs.202508920 (PMC12499454; doi:10.1002/advs.202508920)
Supplement: Supplementary file 1 — Supporting Information [file ADVS-12-e08920-s001.pdf]

ADVANCED SCIENCE
Open Access

Supporting Information

for *Adv. Sci.*, DOI 10.1002/advs.202508920

Luminescent Platform for Thermal Sensing and Imaging Based on Structural Phase-Transition

Anam Javaid, Maja Szymczak, Malgorzata Kubicka, Vasyl Kinzhybalo, Marek Drozd, Damian Szymanski and Lukasz Marciniak**

Supporting Information

Luminescent Platform for Thermal Sensing and Imaging Based on Structural Phase-Transition

Anam Javaid[1], Maja Szymczak[1], Malgorzata Kubicka[1], Vasyl Kinzhybalo[1], Marek Drozd[1], Damian Szymanski[1], L. Marciniak[1]

[1] Institute of Low Temperature and Structure Research, Polish Academy of Sciences, Okólna 2, 50-422 Wrocław, Poland

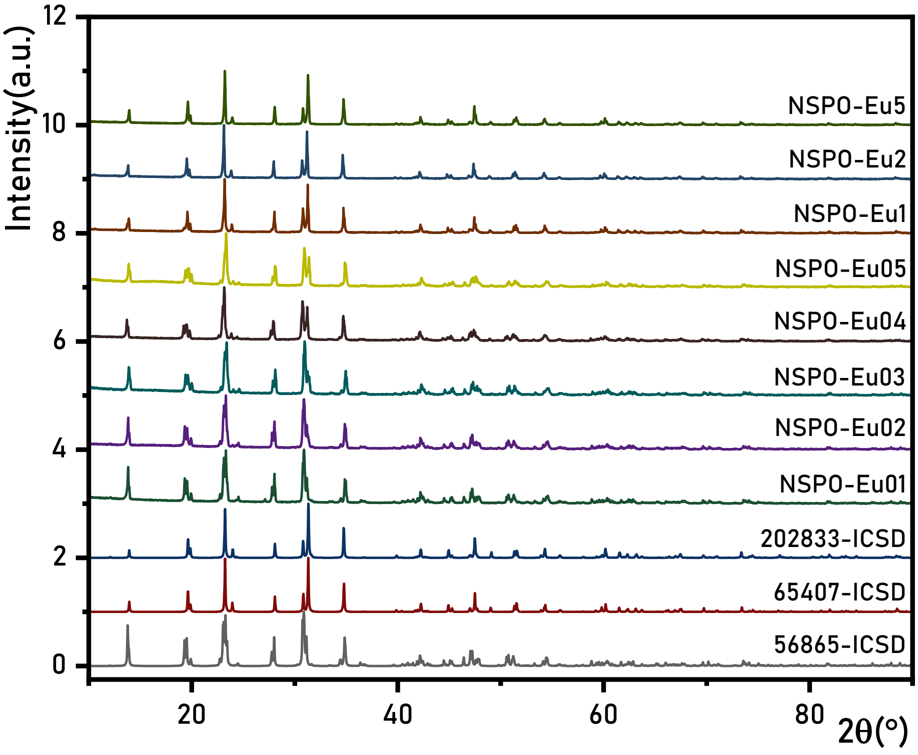

Figure S1. XRD patterns of $Na_3Sc_2(PO_4)_3:Eu^{3+}$ for different Eu^{3+} concentration.

$Na_3Sc_2(PO_4)_3$:0.2%Eu^{3+} measured at 298K

Global Parameters

Number of used phases:	2
Number of variables:	16
Number of constraints:	0
Zero shift/ °2Theta:	0,000000
Specimen displacement/ mm :	0,004(2)
Profile function:	Pseudo Voigt
Background:	Polynomial
R (expected)/ %:	2,79783
R (profile)/ %:	5,37527
R (weighted profile)/ %:	7,53147
GOF:	7,24629
d-statistic:	0,68226
U standard:	0,000000
V standard:	0,000000
W standard:	0,010000
U Left:	0,000000
V Left:	0,000000
W Left:	0,010000
U Right:	0,000000
V Right:	0,000000
W Right:	0,010000
Asymmetry Type:	No Asymmetry Function
Asymmetry 1:	0,000000
Asymmetry 2:	0,000000
Shape Type:	Shape Individual
Shape 1 Left:	0,600000
Shape 2 Left:	0,000000
Shape 3 Left:	0,000000
Shape 1 Right:	0,600000
Shape 2 Right:	0,000000
Shape 3 Right:	0,000000
K a1/a2 intensity ratio:	0,500000
K alpha/beta intensity ratio:	0,000000
Crystal Shape Factor K:	1,0000
Instrumental FWHM Curve Type:	Caglioti function
Instr. Gauss Curve Coefficient A:	0,0045(5)
Instr. Gauss Curve Coefficient B:	-0,0032(9)
Instr. Gauss Curve Coefficient C:	0,0046(3)
Instr. Lorentz Curve Coefficient A:	0,0062(7)
Instr. Lorentz Curve Coefficient B:	-0,004(1)
Instr. Lorentz Curve Coefficient C:	0,0064(5)

Relevant parameters of 65406-ICSD, Na3Sc2(PO4)3-RT

Structure and profile data:	
Formula sum:	$Na_{12,00}P_{12,00}Sc_{8,00}O_{48,00}$
Formula mass/ g/mol:	1775,1820
Density (calculated)/ g/cm^3	2,8290
F(000):	864,0000
Weight fraction/ %:	99(1)
Space group (No.):	C 1 c 1 (9)
Lattice parameters:	
a/ A:	15,3929(5)
b/ A:	8,9193(3)
c/ A:	9,0999(3)
alpha/ °:	90
beta/ °:	123,498(2)
gamma/ °:	90
V/ 10^6 pm^3	1041,84300
Overall displacement parameter:	0,000000
Extinction:	0,000000
Flat Plate Absorption Correction:	0,000000
Porosity:	0,000000
Roughness:	0,000000
Fitting mode:	Structure Fit
U Left:	0,061(6)
V Left:	-0,051(5)

W Left:	0,019(1)
Preferred orientation direction/ hkl:	0,00 0,00 1,00
Preferred orientation parameter:	1,000000
Asymmetry parameter 1:	0,000000
Asymmetry parameter 2:	0,000000
Peak shape:	
parameter 1 Left:	0,74(2)
parameter 2 Left:	0,000000
parameter 3 Left:	0,000000
R (Bragg)/ %:	5,18026

Occupancy, atomic fract. coordinates and Biso for 65406-ICSD, Na3Sc2(PO4)3-RT

Atom	Wyck.	s.o.f.	x	y	z	B/ 10^4 pm^2
Na1	4a	1,000000	0,225700	0,151300	0,704900	3,979423
Na2	4a	1,000000	0,422200	0,390700	0,021100	2,787175
Na3	4a	1,000000	0,091500	0,574900	0,453600	2,613470
P1	4a	1,000000	0,060500	0,108400	0,000399	0,813255
P2	4a	1,000000	0,275700	0,608400	0,020400	0,813255
P3	4a	1,000000	0,421700	0,045300	0,012000	0,821151
Sc1	4a	1,000000	0,318200	0,246200	0,207000	0,647446
Sc2	4a	1,000000	0,017100	0,247700	0,309700	0,647446
O1	4a	1,000000	0,272900	0,437200	0,042800	1,073812
O2	4a	1,000000	0,066300	0,061200	0,479800	1,073812
O3	4a	1,000000	0,483100	0,050400	0,676300	1,113291
O4	4a	1,000000	0,386900	0,051000	0,346000	1,113291
O5	4a	1,000000	0,163600	0,181500	0,054300	1,421222
O6	4a	1,000000	0,172000	0,323100	0,472700	1,421222
O7	4a	1,000000	0,035600	0,128600	0,138500	1,934441
O8	4a	1,000000	0,295700	0,366900	0,376300	1,934441
O9	4a	1,000000	0,473400	0,318600	0,325600	1,176456
O10	4a	1,000000	0,361000	0,679200	0,195000	1,176456
O11	4a	1,000000	0,344300	0,149500	0,020800	1,334370
O12	4a	1,000000	0,000000	0,641900	0,000000	1,334370

Relevant parameters of 74483-ICSD, ScPO4

Structure and profile data:	
Formula sum:	$Sc_{4,00}P_{4,00}O_{16,00}$
Formula mass/ g/mol:	559,7092
Density (calculated)/ g/cm^3	3,7043
F(000):	272,0000
Weight fraction/ %:	1,2(1)
Space group (No.):	I 41/a m d (141)
Lattice parameters:	
a/ A:	6,579000
b/ A:	6,579000
c/ A:	5,796000
alpha/ °:	90
beta/ °:	90
gamma/ °:	90
V/ 10^6 pm^3	250,86960
Overall displacement parameter:	0,000000
Extinction:	0,000000
Flat Plate Absorption Correction:	0,000000
Porosity:	0,000000
Roughness:	0,000000
Fitting mode:	Structure Fit
U Left:	0,000000
V Left:	0,000000
W Left:	0,010000
Preferred orientation direction/ hkl:	0,00 0,00 1,00
Preferred orientation parameter:	1,000000
Asymmetry parameter 1:	0,000000
Asymmetry parameter 2:	0,000000
Peak shape:	
parameter 1 Left:	0,600000
parameter 2 Left:	0,000000

parameter 3 Left: 0,000000
R (Bragg)/ %: 3,01960

Occupancy, atomic fract. coordinates and Biso for 74483-ICSD, ScPO4

Atom	Wyck.	s.o.f.	x	y	z	B/ 10^4 pm^2
Sc1	4a	1,000000	0,000000	0,750000	0,125000	0,000000
P1	4b	1,000000	0,000000	0,250000	0,375000	0,000000
O1	16h	1,000000	0,000000	0,068900	0,208400	0,378992

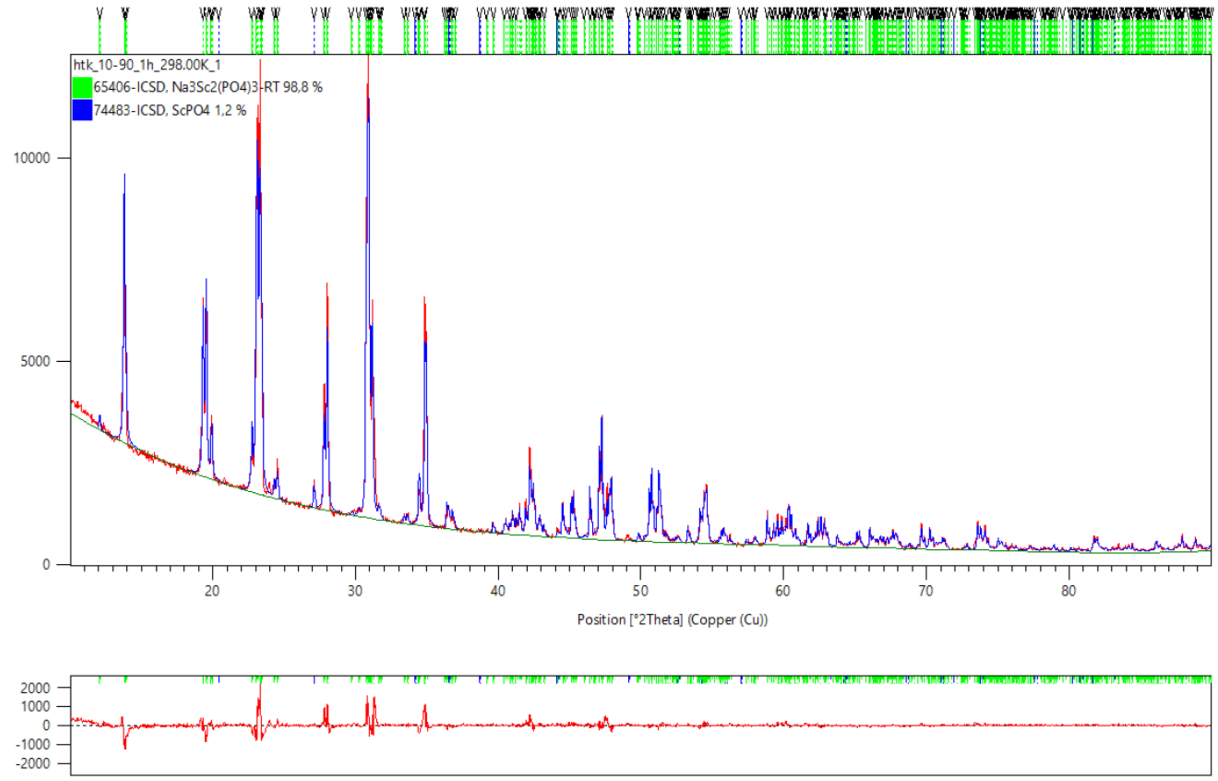

Figure S2. Rietveld refinement of XRD pattern of $Na_3Sc_2(PO_4)_3$:0.2%Eu^{3+} measured at 298K.

$Na_3Sc_2(PO_4)_3$:0.2%Eu^{3+} measured at 303K

Global Parameters

Number of used phases: 2
Number of variables: 16
Number of constraints: 0
Zero shift/ °2Theta: 0,000000
Specimen displacement/ mm : 0,004(2)
Profile function: Pseudo Voigt
Background: Polynomial
R (expected)/ %: 2,79574
R (profile)/ %: 5,27215
R (weighted profile)/ %: 7,41064
GOF: 7,02615
d-statistic: 0,65743
U standard: 0,000000
V standard: 0,000000
W standard: 0,010000

U Left:	0,000000
V Left:	0,000000
W Left:	0,010000
U Right:	0,000000
V Right:	0,000000
W Right:	0,010000
Asymmetry Type:	No Asymmetry Function
Asymmetry 1:	0,000000
Asymmetry 2:	0,000000
Shape Type:	Shape Individual
Shape 1 Left:	0,600000
Shape 2 Left:	0,000000
Shape 3 Left:	0,000000
Shape 1 Right:	0,600000
Shape 2 Right:	0,000000
Shape 3 Right:	0,000000
K a1/a2 intensity ratio:	0,500000
K alpha/beta intensity ratio:	0,000000
Crystal Shape Factor K:	1,0000
Instrumental FWHM Curve Type:	Caglioti function
Instr. Gauss Curve Coefficient A:	0,0045(5)
Instr. Gauss Curve Coefficient B:	-0,0032(9)
Instr. Gauss Curve Coefficient C:	0,0046(3)
Instr. Lorentz Curve Coefficient A:	0,0062(7)
Instr. Lorentz Curve Coefficient B:	-0,004(1)
Instr. Lorentz Curve Coefficient C:	0,0064(5)

Relevant parameters of 65406-ICSD, Na3Sc2(PO4)3-RT

Structure and profile data:	
Formula sum:	$Na_{12,00}P_{12,00}Sc_{8,00}O_{48,00}$
Formula mass/ g/mol:	1775,1820
Density (calculated)/ g/cm^3	2,8290
F(000):	864,0000
Weight fraction/ %:	98,8(5)
Space group (No.):	C 1 c 1 (9)
Lattice parameters:	
a/ A:	15,3933(5)
b/ A:	8,9194(3)
c/ A:	9,0997(3)
alpha/ °:	90
beta/ °:	123,502(2)
gamma/ °:	90
V/ 10^6 pm^3	1041,81500
Overall displacement parameter:	0,000000
Extinction:	0,000000
Flat Plate Absorption Correction:	0,000000
Porosity:	0,000000
Roughness:	0,000000
Fitting mode:	Structure Fit
U Left:	0,062(6)
V Left:	-0,052(5)
W Left:	0,020(1)
Preferred orientation direction/ hkl:	0,00 0,00 1,00
Preferred orientation parameter:	1,000000
Asymmetry parameter 1:	0,000000
Asymmetry parameter 2:	0,000000
Peak shape:	
parameter 1 Left:	0,74(2)
parameter 2 Left:	0,000000
parameter 3 Left:	0,000000
R (Bragg)/ %:	5,15189

Occupancy, atomic fract. coordinates and Biso for 65406-ICSD, Na3Sc2(PO4)3-RT

Atom	Wyck.	s.o.f.	x	y	z	B/ 10^4 pm^2
Na1	4a	1,000000	0,225700	0,151300	0,704900	3,979423
Na2	4a	1,000000	0,422200	0,390700	0,021100	2,787175
Na3	4a	1,000000	0,091500	0,574900	0,453600	2,613470
P1	4a	1,000000	0,060500	0,108400	0,000399	0,813255
P2	4a	1,000000	0,275700	0,608400	0,020400	0,813255
P3	4a	1,000000	0,421700	0,045300	0,012000	0,821151

Sc1	4a	1,000000	0,318200	0,246200	0,207000	0,647446
Sc2	4a	1,000000	0,017100	0,247700	0,309700	0,647446
O1	4a	1,000000	0,272900	0,437200	0,042800	1,073812
O2	4a	1,000000	0,066300	0,061200	0,479800	1,073812
O3	4a	1,000000	0,483100	0,050400	0,676300	1,113291
O4	4a	1,000000	0,386900	0,051000	0,346000	1,113291
O5	4a	1,000000	0,163600	0,181500	0,054300	1,421222
O6	4a	1,000000	0,172000	0,323100	0,472700	1,421222
O7	4a	1,000000	0,035600	0,128600	0,138500	1,934441
O8	4a	1,000000	0,295700	0,366900	0,376300	1,934441
O9	4a	1,000000	0,473400	0,318600	0,325600	1,176456
O10	4a	1,000000	0,361000	0,679200	0,195000	1,176456
O11	4a	1,000000	0,344300	0,149500	0,020800	1,334370
O12	4a	1,000000	0,000000	0,641900	0,000000	1,334370

Relevant parameters of 74483-ICSD, ScPO4

Structure and profile data:	
Formula sum:	$Sc_{4,00}P_{4,00}O_{16,00}$
Formula mass/ g/mol:	559,7092
Density (calculated)/ g/cm^3	3,7043
F(000):	272,0000
Weight fraction/ %:	1,2(1)
Space group (No.):	I 41/a m d (141)
Lattice parameters:	
a/ A:	6,579000
b/ A:	6,579000
c/ A:	5,796000
alpha/ °:	90
beta/ °:	90
gamma/ °:	90
V/ 10^6 pm^3	250,86960
Overall displacement parameter:	0,000000
Extinction:	0,000000
Flat Plate Absorption Correction:	0,000000
Porosity:	0,000000
Roughness:	0,000000
Fitting mode:	Structure Fit
U Left:	0,000000
V Left:	0,000000
W Left:	0,010000
Preferred orientation direction/ hkl:	0,00 0,00 1,00
Preferred orientation parameter:	1,000000
Asymmetry parameter 1:	0,000000
Asymmetry parameter 2:	0,000000
Peak shape:	
parameter 1 Left:	0,600000
parameter 2 Left:	0,000000
parameter 3 Left:	0,000000
R (Bragg)/ %:	3,04214

Occupancy, atomic fract. coordinates and Biso for 74483-ICSD, ScPO4

Atom	Wyck.	s.o.f.	x	y	z	B/ 10^4 pm^2
Sc1	4a	1,000000	0,000000	0,750000	0,125000	0,000000
P1	4b	1,000000	0,000000	0,250000	0,375000	0,000000
O1	16h	1,000000	0,000000	0,068900	0,208400	0,378992

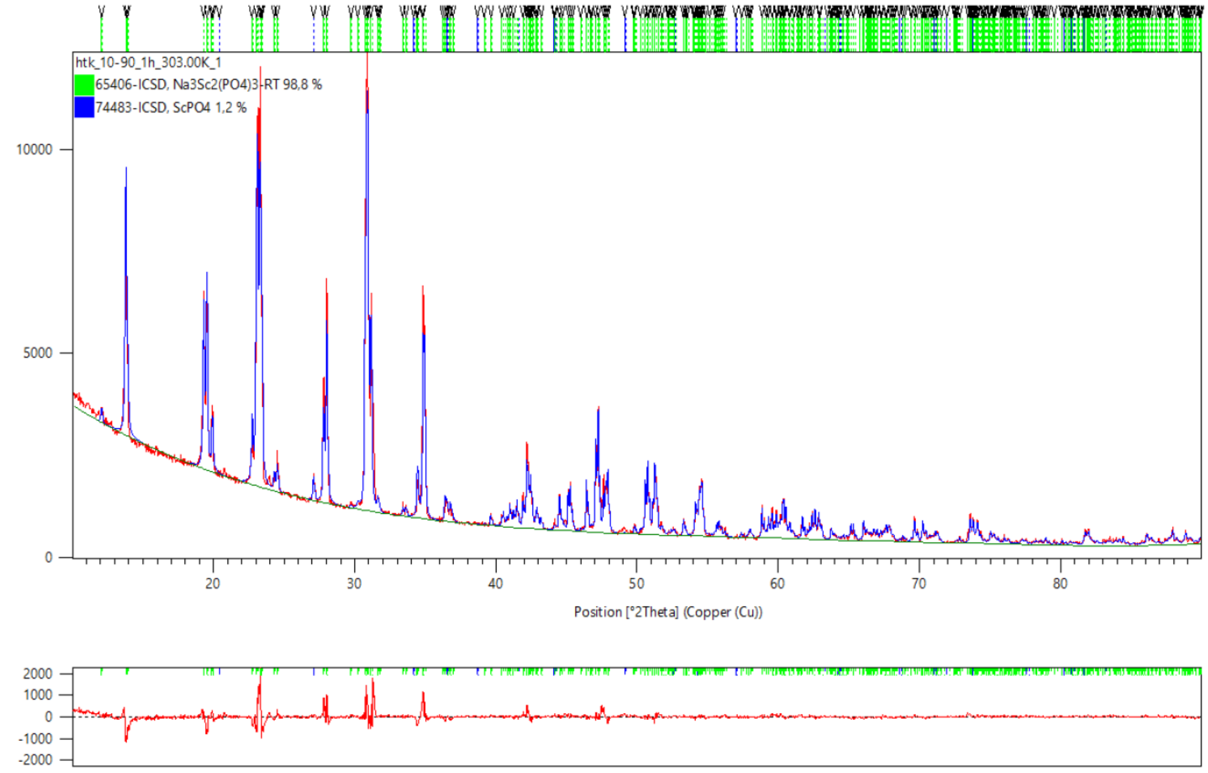

Figure S3. Rietveld refinement of XRD pattern of $Na_3Sc_2(PO_4)_3$:0.2%Eu^{3+} measured at 303K.

$Na_3Sc_2(PO_4)_3$:0.2%Eu^{3+} measured at 313K

Global Parameters

Number of used phases:	2
Number of variables:	16
Number of constraints:	0
Zero shift/ °2Theta:	0,000000
Specimen displacement/ mm :	-0,102(2)
Profile function:	Pseudo Voigt
Background:	Polynomial
R (expected)/ %:	2,80271
R (profile)/ %:	5,31236
R (weighted profile)/ %:	7,53314
GOF:	7,22428
d-statistic:	0,60032
U standard:	0,000000
V standard:	0,000000
W standard:	0,010000
U Left:	0,000000
V Left:	0,000000
W Left:	0,010000
U Right:	0,000000
V Right:	0,000000
W Right:	0,010000
Asymmetry Type:	No Asymmetry Function
Asymmetry 1:	0,000000
Asymmetry 2:	0,000000
Shape Type:	Shape Individual
Shape 1 Left:	0,600000
Shape 2 Left:	0,000000
Shape 3 Left:	0,000000
Shape 1 Right:	0,600000
Shape 2 Right:	0,000000
Shape 3 Right:	0,000000
K a1/a2 intensity ratio:	0,500000

K alpha/beta intensity ratio:	0,000000
Crystal Shape Factor K:	1,0000
Instrumental FWHM Curve Type:	Caglioti function
Instr. Gauss Curve Coefficient A:	0,0045(5)
Instr. Gauss Curve Coefficient B:	-0,0032(9)
Instr. Gauss Curve Coefficient C:	0,0046(3)
Instr. Lorentz Curve Coefficient A:	0,0062(7)
Instr. Lorentz Curve Coefficient B:	-0,004(1)
Instr. Lorentz Curve Coefficient C:	0,0064(5)

Relevant parameters of 65406-ICSD, Na3Sc2(PO4)3-RT

Structure and profile data:	
Formula sum:	$Na_{12,00}P_{12,00}Sc_{8,00}O_{48,00}$
Formula mass/ g/mol:	1775,1820
Density (calculated)/ g/cm^3	2,8303
F(000):	864,0000
Weight fraction/ %:	99(1)
Space group (No.):	C 1 c 1 (9)
Lattice parameters:	
a/ A:	15,3920(5)
b/ A:	8,9188(3)
c/ A:	9,0980(3)
alpha/ °:	90
beta/ °:	123,511(2)
gamma/ °:	90
V/ 10^6 pm^3	1041,35000
Overall displacement parameter:	0,000000
Extinction:	0,000000
Flat Plate Absorption Correction:	0,000000
Porosity:	0,000000
Roughness:	0,000000
Fitting mode:	Structure Fit
U Left:	0,059(6)
V Left:	-0,050(5)
W Left:	0,020(1)
Preferred orientation direction/ hkl:	0,00 0,00 1,00
Preferred orientation parameter:	1,000000
Asymmetry parameter 1:	0,000000
Asymmetry parameter 2:	0,000000
Peak shape:	
parameter 1 Left:	0,72(2)
parameter 2 Left:	0,000000
parameter 3 Left:	0,000000
R (Bragg)/ %:	5,23562

Occupancy, atomic fract. coordinates and Biso for 65406-ICSD, Na3Sc2(PO4)3-RT

Atom	Wyck.	s.o.f.	x	y	z	B/ 10^4 pm^2
Na1	4a	1,000000	0,225700	0,151300	0,704900	3,979423
Na2	4a	1,000000	0,422200	0,390700	0,021100	2,787175
Na3	4a	1,000000	0,091500	0,574900	0,453600	2,613470
P1	4a	1,000000	0,060500	0,108400	0,000399	0,813255
P2	4a	1,000000	0,275700	0,608400	0,020400	0,813255
P3	4a	1,000000	0,421700	0,045300	0,012000	0,821151
Sc1	4a	1,000000	0,318200	0,246200	0,207000	0,647446
Sc2	4a	1,000000	0,017100	0,247700	0,309700	0,647446
O1	4a	1,000000	0,272900	0,437200	0,042800	1,073812
O2	4a	1,000000	0,066300	0,061200	0,479800	1,073812
O3	4a	1,000000	0,483100	0,050400	0,676300	1,113291
O4	4a	1,000000	0,386900	0,051000	0,346000	1,113291
O5	4a	1,000000	0,163600	0,181500	0,054300	1,421222
O6	4a	1,000000	0,172000	0,323100	0,472700	1,421222
O7	4a	1,000000	0,035600	0,128600	0,138500	1,934441
O8	4a	1,000000	0,295700	0,366900	0,376300	1,934441
O9	4a	1,000000	0,473400	0,318600	0,325600	1,176456
O10	4a	1,000000	0,361000	0,679200	0,195000	1,176456
O11	4a	1,000000	0,344300	0,149500	0,020800	1,334370
O12	4a	1,000000	0,000000	0,641900	0,000000	1,334370

Relevant parameters of 74483-ICSD, ScPO4

Structure and profile data:	
Formula sum:	$Sc_{4,00}P_{4,00}O_{16,00}$
Formula mass/ g/mol:	559,7092
Density (calculated)/ g/cm^3	3,7043
F(000):	272,0000
Weight fraction/ %:	1,3(1)
Space group (No.):	I 41/a m d (141)
Lattice parameters:	
a/ A:	6,579000
b/ A:	6,579000
c/ A:	5,796000
alpha/ °:	90
beta/ °:	90
gamma/ °:	90
V/ 10^6 pm^3	250,86960
Overall displacement parameter:	0,000000
Extinction:	0,000000
Flat Plate Absorption Correction:	0,000000
Porosity:	0,000000
Roughness:	0,000000
Fitting mode:	Structure Fit
U Left:	0,000000
V Left:	0,000000
W Left:	0,010000
Preferred orientation direction/ hkl:	0,00 0,00 1,00
Preferred orientation parameter:	1,000000
Asymmetry parameter 1:	0,000000
Asymmetry parameter 2:	0,000000
Peak shape:	
parameter 1 Left:	0,600000
parameter 2 Left:	0,000000
parameter 3 Left:	0,000000
R (Bragg)/ %:	3,02039

Occupancy, atomic fract. coordinates and Biso for 74483-ICSD, ScPO4

Atom	Wyck.	s.o.f.	x	y	z	B/ 10^4 pm^2
Sc1	4a	1,000000	0,000000	0,750000	0,125000	0,000000
P1	4b	1,000000	0,000000	0,250000	0,375000	0,000000
O1	16h	1,000000	0,000000	0,068900	0,208400	0,378992

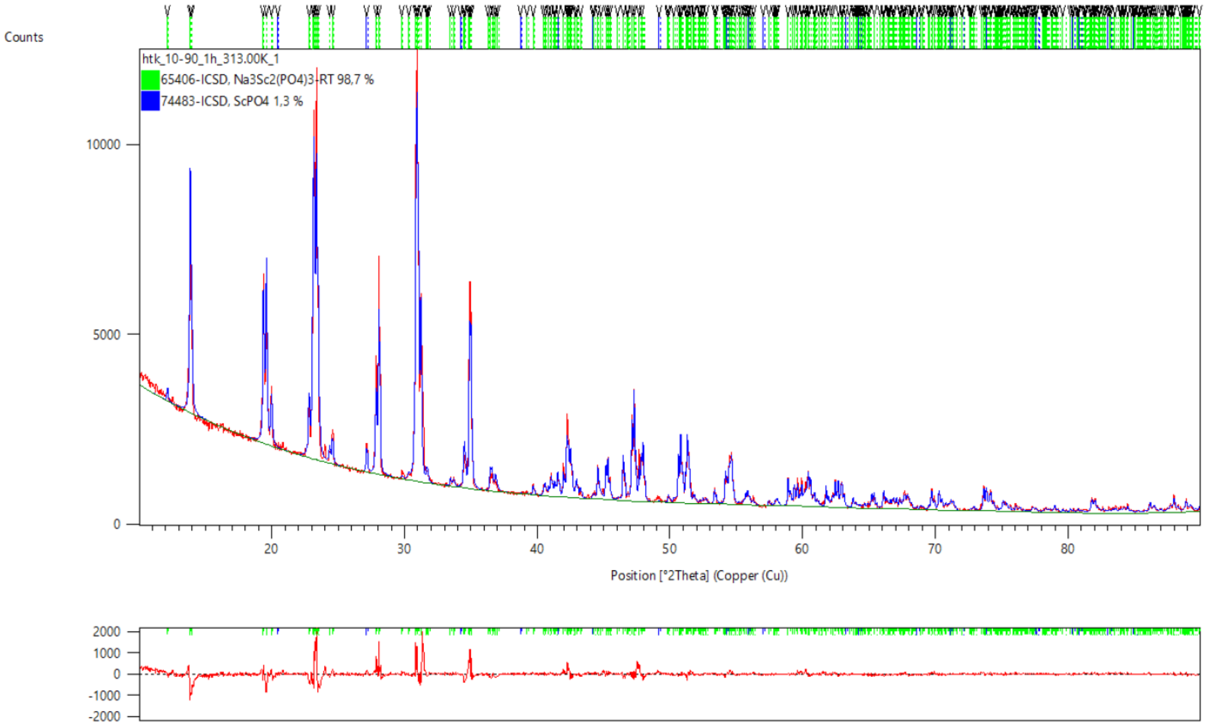

Figure S4. Rietveld refinement of XRD pattern of $Na_3Sc_2(PO_4)_3$:0.2%Eu^{3+} measured at 313K.

$Na_3Sc_2(PO_4)_3$:0.2%Eu^{3+} measured at 323K

Global Parameters

Number of used phases:	3
Number of variables:	19
Number of constraints:	1
Zero shift/ °2Theta:	0,000000
Specimen displacement/ mm :	-0,144(2)
Profile function:	Pseudo Voigt
Background:	Polynomial
R (expected)/ %:	2,80091
R (profile)/ %:	4,93608
R (weighted profile)/ %:	6,69195
GOF:	5,70831
d-statistic:	0,74964
U standard:	0,000000
V standard:	0,000000
W standard:	0,010000
U Left:	0,000000
V Left:	0,000000
W Left:	0,010000
U Right:	0,000000
V Right:	0,000000
W Right:	0,010000
Asymmetry Type:	No Asymmetry Function
Asymmetry 1:	0,000000
Asymmetry 2:	0,000000
Shape Type:	Shape Individual
Shape 1 Left:	0,600000
Shape 2 Left:	0,000000
Shape 3 Left:	0,000000
Shape 1 Right:	0,600000
Shape 2 Right:	0,000000
Shape 3 Right:	0,000000
K a1/a2 intensity ratio:	0,500000

K alpha/beta intensity ratio:	0,000000
Crystal Shape Factor K:	1,0000
Instrumental FWHM Curve Type:	Caglioti function
Instr. Gauss Curve Coefficient A:	0,0045(5)
Instr. Gauss Curve Coefficient B:	-0,0032(9)
Instr. Gauss Curve Coefficient C:	0,0046(3)
Instr. Lorentz Curve Coefficient A:	0,0062(7)
Instr. Lorentz Curve Coefficient B:	-0,004(1)
Instr. Lorentz Curve Coefficient C:	0,0064(5)

Relevant parameters of 65406-ICSD, Na3Sc2(PO4)3-RT

Structure and profile data:	
Formula sum:	$Na_{12'00}P_{12'00}Sc_{8'00}O_{48'00}$
Formula mass/ g/mol:	1775,1820
Density (calculated)/ g/cm^3	2,8314
F(000):	864,0000
Weight fraction/ %:	87(1)
Space group (No.):	C 1 c 1 (9)
Lattice parameters:	
a/ A:	15,3905(5)
b/ A:	8,9178(3)
c/ A:	9,0973(3)
alpha/ °:	90
beta/ °:	123,519(2)
gamma/ °:	90
V/ 10^6 pm^3	1040,96200
Overall displacement parameter:	0,000000
Extinction:	0,000000
Flat Plate Absorption Correction:	0,000000
Porosity:	0,000000
Roughness:	0,000000
Fitting mode:	Structure Fit
U Left:	0,062(6)
V Left:	-0,052(5)
W Left:	0,019(1)
Preferred orientation direction/ hkl:	0,00 0,00 1,00
Preferred orientation parameter:	1,000000
Asymmetry parameter 1:	0,000000
Asymmetry parameter 2:	0,000000
Peak shape:	
parameter 1 Left:	0,62(2)
parameter 2 Left:	0,000000
parameter 3 Left:	0,000000
R (Bragg)/ %:	4,99081

Occupancy, atomic fract. coordinates and Biso for 65406-ICSD, Na3Sc2(PO4)3-RT

Atom	Wyck.	s.o.f.	x	y	z	B/ 10^4 pm^2
Na1	4a	1,000000	0,225700	0,151300	0,704900	3,979423
Na2	4a	1,000000	0,422200	0,390700	0,021100	2,787175
Na3	4a	1,000000	0,091500	0,574900	0,453600	2,613470
P1	4a	1,000000	0,060500	0,108400	0,000399	0,813255
P2	4a	1,000000	0,275700	0,608400	0,020400	0,813255
P3	4a	1,000000	0,421700	0,045300	0,012000	0,821151
Sc1	4a	1,000000	0,318200	0,246200	0,207000	0,647446
Sc2	4a	1,000000	0,017100	0,247700	0,309700	0,647446
O1	4a	1,000000	0,272900	0,437200	0,042800	1,073812
O2	4a	1,000000	0,066300	0,061200	0,479800	1,073812
O3	4a	1,000000	0,483100	0,050400	0,676300	1,113291
O4	4a	1,000000	0,386900	0,051000	0,346000	1,113291
O5	4a	1,000000	0,163600	0,181500	0,054300	1,421222
O6	4a	1,000000	0,172000	0,323100	0,472700	1,421222
O7	4a	1,000000	0,035600	0,128600	0,138500	1,934441
O8	4a	1,000000	0,295700	0,366900	0,376300	1,934441
O9	4a	1,000000	0,473400	0,318600	0,325600	1,176456
O10	4a	1,000000	0,361000	0,679200	0,195000	1,176456
O11	4a	1,000000	0,344300	0,149500	0,020800	1,334370
O12	4a	1,000000	0,000000	0,641900	0,000000	1,334370

Relevant parameters of 202834-ICSD, Na3Sc2(PO4)3-HT

Structure and profile data:	
Formula sum:	$Sc_{12,00}P_{18,00}O_{72,00}Na_{7,16}$
Formula mass/ g/mol:	2413,6550
Density (calculated)/ g/cm^3	2,6027
F(000):	1176,8040
Weight fraction/ %:	11,6(3)
Space group (No.):	R -3 c (167)
Lattice parameters:	
a/ A:	8,9260(6)
b/ A:	8,9260(6)
c/ A:	22,315(2)
alpha/ °:	90
beta/ °:	90
gamma/ °:	120
V/ 10^6 pm^3	1539,72300
Overall displacement parameter:	0,000000
Extinction:	0,000000
Flat Plate Absorption Correction:	0,000000
Porosity:	0,000000
Roughness:	0,000000
Fitting mode:	Structure Fit
U Left:	0,341033
V Left:	-0,245277
W Left:	0,048326
Preferred orientation direction/ hkl:	0,00 0,00 1,00
Preferred orientation parameter:	1,000000
Asymmetry parameter 1:	0,000000
Asymmetry parameter 2:	0,000000
Peak shape:	
parameter 1 Left:	0,635570
parameter 2 Left:	0,000000
parameter 3 Left:	0,000000
R (Bragg)/ %:	6,20972

Occupancy, atomic fract. coordinates and Biso for 202834-ICSD, Na3Sc2(PO4)3-HT

Atom	Wyck.	s.o.f.	x	y	z	B/ 10^4 pm^2
Sc1	12c	1,000000	0,000000	0,000000	0,148700	0,000000
P1	18e	1,000000	0,295100	0,000000	0,250000	0,000000
O1	36f	1,000000	0,023500	0,209100	0,194600	0,000000
O2	36f	1,000000	0,192170	0,172830	0,088730	0,000000
Na1	18e	0,347000	0,637200	0,000000	0,250000	0,000000
Na2	6b	0,153000	0,000000	0,000000	0,000000	0,000000

Relevant parameters of 74483-ICSD, ScPO4

Structure and profile data:	
Formula sum:	$Sc_{4,00}P_{4,00}O_{16,00}$
Formula mass/ g/mol:	559,7092
Density (calculated)/ g/cm^3	3,7043
F(000):	272,0000
Weight fraction/ %:	1,16(9)
Space group (No.):	I 41/a m d (141)
Lattice parameters:	
a/ A:	6,579000
b/ A:	6,579000
c/ A:	5,796000
alpha/ °:	90
beta/ °:	90
gamma/ °:	90
V/ 10^6 pm^3	250,86960
Overall displacement parameter:	0,000000
Extinction:	0,000000
Flat Plate Absorption Correction:	0,000000
Porosity:	0,000000
Roughness:	0,000000
Fitting mode:	Structure Fit

U Left:	0,000000
V Left:	0,000000
W Left:	0,010000
Preferred orientation direction/ hkl:	0,00 0,00 1,00
Preferred orientation parameter:	1,000000
Asymmetry parameter 1:	0,000000
Asymmetry parameter 2:	0,000000
Peak shape:	
parameter 1 Left:	0,600000
parameter 2 Left:	0,000000
parameter 3 Left:	0,000000
R (Bragg)/ %:	4,78571

Occupancy, atomic fract. coordinates and Biso for 74483-ICSD, ScPO4

Atom	Wyck.	s.o.f.	x	y	z	B/ 10^4 pm^2
Sc1	4a	1,000000	0,000000	0,750000	0,125000	0,000000
P1	4b	1,000000	0,000000	0,250000	0,375000	0,000000
O1	16h	1,000000	0,000000	0,068900	0,208400	0,378992

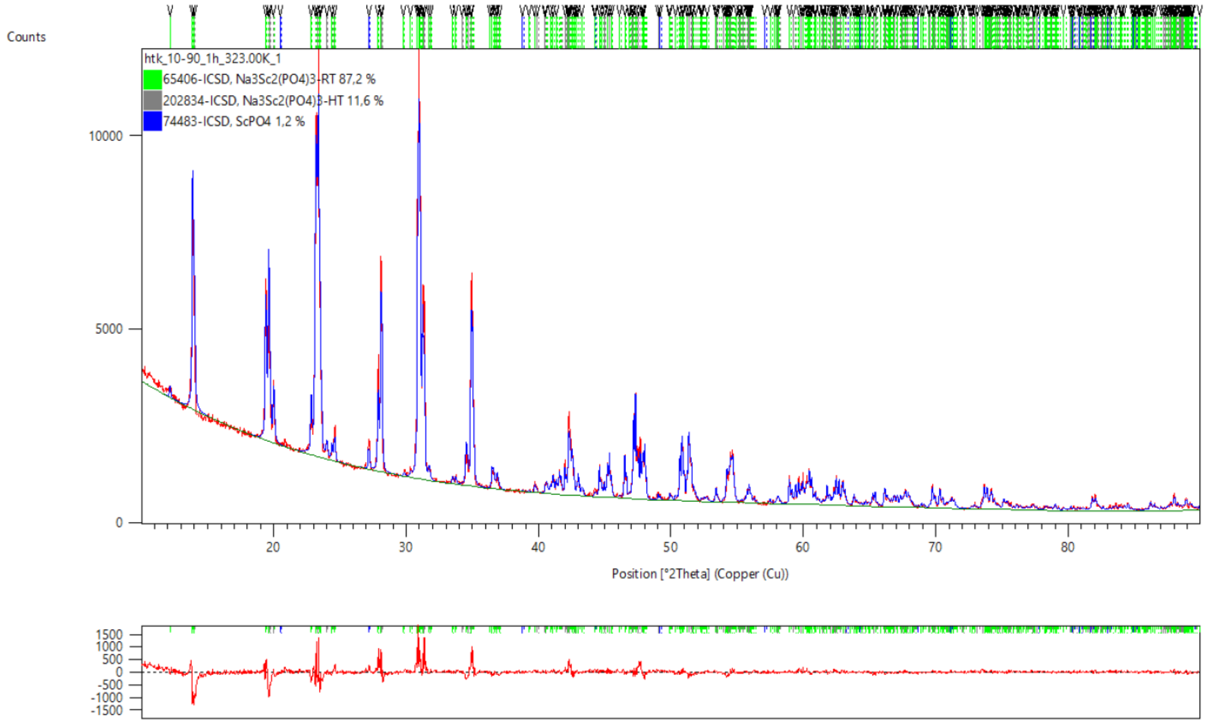

Figure S5. Rietveld refinement of XRD pattern of $Na_3Sc_2(PO_4)_3$:0.2%Eu^{3+} measured at 323K.

$Na_3Sc_2(PO_4)_3$:0.2%Eu^{3+} measured at 333K

Global Parameters

Number of used phases:	3
Number of variables:	19
Number of constraints:	1
Zero shift/ °2Theta:	0,000000
Specimen displacement/ mm :	-0,125(2)
Profile function:	Pseudo Voigt
Background:	Polynomial

R (expected)/ %:	2,79867
R (profile)/ %:	5,42409
R (weighted profile)/ %:	7,44205
GOF:	7,07101
d-statistic:	0,57147
U standard:	0,000000
V standard:	0,000000
W standard:	0,010000
U Left:	0,000000
V Left:	0,000000
W Left:	0,010000
U Right:	0,000000
V Right:	0,000000
W Right:	0,010000
Asymmetry Type:	No Asymmetry Function
Asymmetry 1:	0,000000
Asymmetry 2:	0,000000
Shape Type:	Shape Individual
Shape 1 Left:	0,600000
Shape 2 Left:	0,000000
Shape 3 Left:	0,000000
Shape 1 Right:	0,600000
Shape 2 Right:	0,000000
Shape 3 Right:	0,000000
K a1/a2 intensity ratio:	0,500000
K alpha/beta intensity ratio:	0,000000
Crystal Shape Factor K:	1,0000
Instrumental FWHM Curve Type:	Caglioti function
Instr. Gauss Curve Coefficient A:	0,0045(5)
Instr. Gauss Curve Coefficient B:	-0,0032(9)
Instr. Gauss Curve Coefficient C:	0,0046(3)
Instr. Lorentz Curve Coefficient A:	0,0062(7)
Instr. Lorentz Curve Coefficient B:	-0,004(1)
Instr. Lorentz Curve Coefficient C:	0,0064(5)

Relevant parameters of 65406-ICSD, Na3Sc2(PO4)3-RT

Structure and profile data:	
Formula sum:	$Na_{12{,}00}P_{12{,}00}Sc_{8{,}00}O_{48{,}00}$
Formula mass/ g/mol:	1775,1820
Density (calculated)/ g/cm^3	2,8317
F(000):	864,0000
Weight fraction/ %:	73(1)
Space group (No.):	C 1 c 1 (9)
Lattice parameters:	
a/ A:	15,3901(7)
b/ A:	8,9177(4)
c/ A:	9,0971(4)
alpha/ °:	90
beta/ °:	123,525(3)
gamma/ °:	90
V/ 10^6 pm^3	1040,82000
Overall displacement parameter:	0,000000
Extinction:	0,000000
Flat Plate Absorption Correction:	0,000000
Porosity:	0,000000
Roughness:	0,000000
Fitting mode:	Structure Fit
U Left:	0,068(9)
V Left:	-0,054(7)
W Left:	0,020(1)
Preferred orientation direction/ hkl:	0,00 0,00 1,00
Preferred orientation parameter:	1,000000
Asymmetry parameter 1:	0,000000
Asymmetry parameter 2:	0,000000
Peak shape:	
parameter 1 Left:	0,60(3)
parameter 2 Left:	0,000000
parameter 3 Left:	0,000000
R (Bragg)/ %:	5,89248

Occupancy, atomic fract. coordinates and Biso for 65406-ICSD, Na3Sc2(PO4)3-RT

Atom	Wyck.	s.o.f.	x	y	z	B/ 10^4 pm^2

Na1	4a	1,000000	0,225700	0,151300	0,704900	3,979423
Na2	4a	1,000000	0,422200	0,390700	0,021100	2,787175
Na3	4a	1,000000	0,091500	0,574900	0,453600	2,613470
P1	4a	1,000000	0,060500	0,108400	0,000399	0,813255
P2	4a	1,000000	0,275700	0,608400	0,020400	0,813255
P3	4a	1,000000	0,421700	0,045300	0,012000	0,821151
Sc1	4a	1,000000	0,318200	0,246200	0,207000	0,647446
Sc2	4a	1,000000	0,017100	0,247700	0,309700	0,647446
O1	4a	1,000000	0,272900	0,437200	0,042800	1,073812
O2	4a	1,000000	0,066300	0,061200	0,479800	1,073812
O3	4a	1,000000	0,483100	0,050400	0,676300	1,113291
O4	4a	1,000000	0,386900	0,051000	0,346000	1,113291
O5	4a	1,000000	0,163600	0,181500	0,054300	1,421222
O6	4a	1,000000	0,172000	0,323100	0,472700	1,421222
O7	4a	1,000000	0,035600	0,128600	0,138500	1,934441
O8	4a	1,000000	0,295700	0,366900	0,376300	1,934441
O9	4a	1,000000	0,473400	0,318600	0,325600	1,176456
O10	4a	1,000000	0,361000	0,679200	0,195000	1,176456
O11	4a	1,000000	0,344300	0,149500	0,020800	1,334370
O12	4a	1,000000	0,000000	0,641900	0,000000	1,334370

Relevant parameters of 202834-ICSD, Na3Sc2(PO4)3-HT

Structure and profile data:	
Formula sum:	$Sc_{12,00}P_{18,00}O_{72,00}Na_{7,16}$
Formula mass/ g/mol:	2413,6550
Density (calculated)/ g/cm^3	2,6022
F(000):	1176,8040
Weight fraction/ %:	25,5(3)
Space group (No.):	R -3 c (167)
Lattice parameters:	
a/ A:	8,9222(4)
b/ A:	8,9222(4)
c/ A:	22,338(1)
alpha/ °:	90
beta/ °:	90
gamma/ °:	120
V/ 10^6 pm^3	1539,99300
Overall displacement parameter:	0,000000
Extinction:	0,000000
Flat Plate Absorption Correction:	0,000000
Porosity:	0,000000
Roughness:	0,000000
Fitting mode:	Structure Fit
U Left:	0,341033
V Left:	-0,245277
W Left:	0,048326
Preferred orientation direction/ hkl:	0,00 0,00 1,00
Preferred orientation parameter:	1,000000
Asymmetry parameter 1:	0,000000
Asymmetry parameter 2:	0,000000
Peak shape:	
parameter 1 Left:	0,635570
parameter 2 Left:	0,000000
parameter 3 Left:	0,000000
R (Bragg)/ %:	7,98932

Occupancy, atomic fract. coordinates and Biso for 202834-ICSD, Na3Sc2(PO4)3-HT

Atom	Wyck.	s.o.f.	x	y	z	B/ 10^4 pm^2
Sc1	12c	1,000000	0,000000	0,000000	0,148700	0,000000
P1	18e	1,000000	0,295100	0,000000	0,250000	0,000000
O1	36f	1,000000	0,023500	0,209100	0,194600	0,000000
O2	36f	1,000000	0,192170	0,172830	0,088730	0,000000
Na1	18e	0,347000	0,637200	0,000000	0,250000	0,000000
Na2	6b	0,153000	0,000000	0,000000	0,000000	0,000000

Relevant parameters of 74483-ICSD, ScPO4

Structure and profile data:	
Formula sum:	$Sc_{4,00}P_{4,00}O_{16,00}$
Formula mass/ g/mol:	559,7092
Density (calculated)/ g/cm^3	3,7043
F(000):	272,0000
Weight fraction/ %:	1,3(1)
Space group (No.):	I 41/a m d (141)
Lattice parameters:	
a/ A:	6,579000
b/ A:	6,579000
c/ A:	5,796000
alpha/ °:	90
beta/ °:	90
gamma/ °:	90
V/ 10^6 pm^3	250,86960
Overall displacement parameter:	0,000000
Extinction:	0,000000
Flat Plate Absorption Correction:	0,000000
Porosity:	0,000000
Roughness:	0,000000
Fitting mode:	Structure Fit
U Left:	0,000000
V Left:	0,000000
W Left:	0,010000
Preferred orientation direction/ hkl:	0,00 0,00 1,00
Preferred orientation parameter:	1,000000
Asymmetry parameter 1:	0,000000
Asymmetry parameter 2:	0,000000
Peak shape:	
parameter 1 Left:	0,600000
parameter 2 Left:	0,000000
parameter 3 Left:	0,000000
R (Bragg)/ %:	4,53822

Occupancy, atomic fract. coordinates and Biso for 74483-ICSD, ScPO4

Atom	Wyck.	s.o.f.	x	y	z	B/ 10^4 pm^2
Sc1	4a	1,000000	0,000000	0,750000	0,125000	0,000000
P1	4b	1,000000	0,000000	0,250000	0,375000	0,000000
O1	16h	1,000000	0,000000	0,068900	0,208400	0,378992

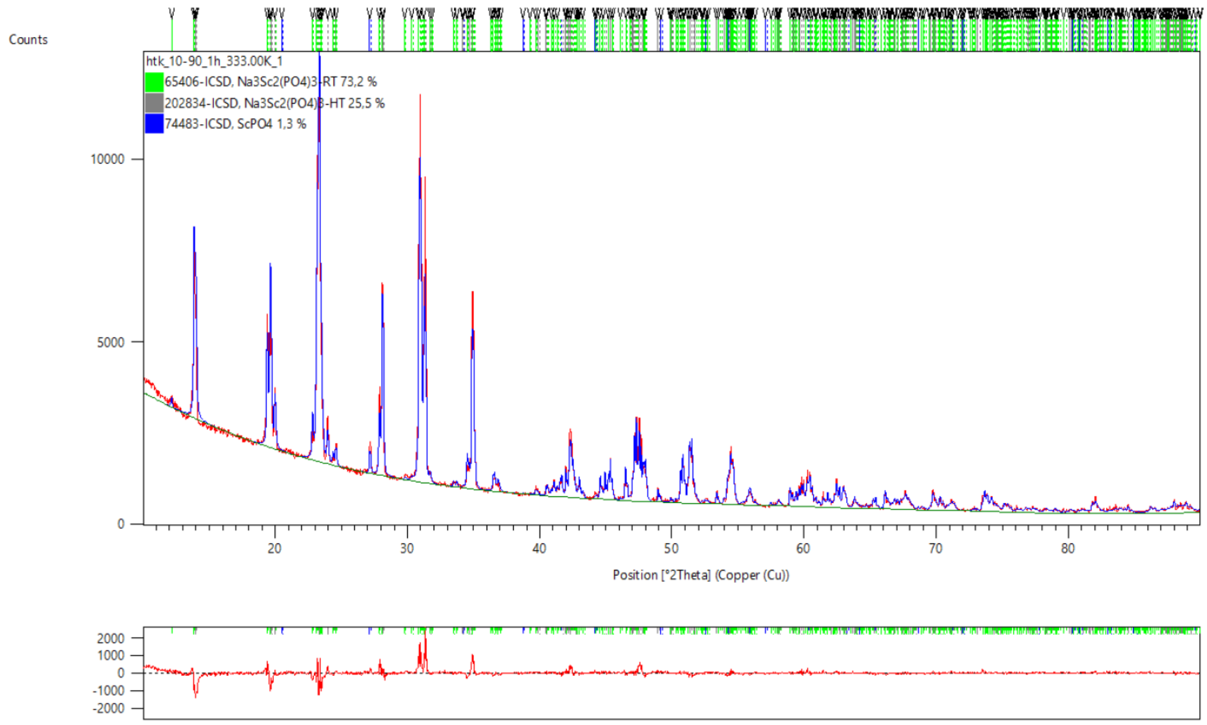

Figure S6. Rietveld refinement of XRD pattern of $Na_3Sc_2(PO_4)_3$:0.2%Eu^{3+} measured at 333K.

$Na_3Sc_2(PO_4)_3$:0.2%Eu^{3+} measured at 343K

Global Parameters

Number of used phases:	3
Number of variables:	19
Number of constraints:	1
Zero shift/ °2Theta:	0,000000
Specimen displacement/ mm :	-0,154(2)
Profile function:	Pseudo Voigt
Background:	Polynomial
R (expected)/ %:	2,81126
R (profile)/ %:	6,61390
R (weighted profile)/ %:	9,81182
GOF:	12,18137
d-statistic:	0,33717
U standard:	0,000000
V standard:	0,000000
W standard:	0,010000
U Left:	0,000000
V Left:	0,000000
W Left:	0,010000
U Right:	0,000000
V Right:	0,000000
W Right:	0,010000
Asymmetry Type:	No Asymmetry Function
Asymmetry 1:	0,000000
Asymmetry 2:	0,000000
Shape Type:	Shape Individual
Shape 1 Left:	0,600000
Shape 2 Left:	0,000000
Shape 3 Left:	0,000000
Shape 1 Right:	0,600000
Shape 2 Right:	0,000000
Shape 3 Right:	0,000000
K a1/a2 intensity ratio:	0,500000
K alpha/beta intensity ratio:	0,000000
Crystal Shape Factor K:	1,0000

Instrumental FWHM Curve Type:	Caglioti function
Instr. Gauss Curve Coefficient A:	0,0045(5)
Instr. Gauss Curve Coefficient B:	-0,0032(9)
Instr. Gauss Curve Coefficient C:	0,0046(3)
Instr. Lorentz Curve Coefficient A:	0,0062(7)
Instr. Lorentz Curve Coefficient B:	-0,004(1)
Instr. Lorentz Curve Coefficient C:	0,0064(5)

Relevant parameters of 65406-ICSD, Na3Sc2(PO4)3-RT

Structure and profile data:	
Formula sum:	$Na_{12,00}P_{12,00}Sc_{8,00}O_{48,00}$
Formula mass/ g/mol:	1775,1820
Density (calculated)/ g/cm^3	2,8300
F(000):	864,0000
Weight fraction/ %:	43(1)
Space group (No.):	C 1 c 1 (9)
Lattice parameters:	
a/ A:	15,392(2)
b/ A:	8,9194(8)
c/ A:	9,1002(8)
alpha/ °:	90
beta/ °:	123,531(6)
gamma/ °:	90
V/ 10^6 pm^3	1041,46500
Overall displacement parameter:	0,000000
Extinction:	0,000000
Flat Plate Absorption Correction:	0,000000
Porosity:	0,000000
Roughness:	0,000000
Fitting mode:	Structure Fit
U Left:	0,068212
V Left:	-0,053953
W Left:	0,019893
Preferred orientation direction/ hkl:	0,00 0,00 1,00
Preferred orientation parameter:	1,000000
Asymmetry parameter 1:	0,000000
Asymmetry parameter 2:	0,000000
Peak shape:	
parameter 1 Left:	0,601942
parameter 2 Left:	0,000000
parameter 3 Left:	0,000000
R (Bragg)/ %:	8,18913

Occupancy, atomic fract. coordinates and Biso for 65406-ICSD, Na3Sc2(PO4)3-RT

Atom	Wyck.	s.o.f.	x	y	z	B/ 10^4 pm^2
Na1	4a	1,000000	0,225700	0,151300	0,704900	3,979423
Na2	4a	1,000000	0,422200	0,390700	0,021100	2,787175
Na3	4a	1,000000	0,091500	0,574900	0,453600	2,613470
P1	4a	1,000000	0,060500	0,108400	0,000399	0,813255
P2	4a	1,000000	0,275700	0,608400	0,020400	0,813255
P3	4a	1,000000	0,421700	0,045300	0,012000	0,821151
Sc1	4a	1,000000	0,318200	0,246200	0,207000	0,647446
Sc2	4a	1,000000	0,017100	0,247700	0,309700	0,647446
O1	4a	1,000000	0,272900	0,437200	0,042800	1,073812
O2	4a	1,000000	0,066300	0,061200	0,479800	1,073812
O3	4a	1,000000	0,483100	0,050400	0,676300	1,113291
O4	4a	1,000000	0,386900	0,051000	0,346000	1,113291
O5	4a	1,000000	0,163600	0,181500	0,054300	1,421222
O6	4a	1,000000	0,172000	0,323100	0,472700	1,421222
O7	4a	1,000000	0,035600	0,128600	0,138500	1,934441
O8	4a	1,000000	0,295700	0,366900	0,376300	1,934441
O9	4a	1,000000	0,473400	0,318600	0,325600	1,176456
O10	4a	1,000000	0,361000	0,679200	0,195000	1,176456
O11	4a	1,000000	0,344300	0,149500	0,020800	1,334370
O12	4a	1,000000	0,000000	0,641900	0,000000	1,334370

Relevant parameters of 202834-ICSD, Na3Sc2(PO4)3-HT

Structure and profile data:	
Formula sum:	$Sc_{12,00}P_{18,00}O_{72,00}Na_{7,16}$
Formula mass/ g/mol:	2413,6550
Density (calculated)/ g/cm^3	2,6009
F(000):	1176,8040
Weight fraction/ %:	56(1)
Space group (No.):	R -3 c (167)
Lattice parameters:	
a/ A:	8,9206(4)
b/ A:	8,9206(4)
c/ A:	22,358(1)
alpha/ °:	90
beta/ °:	90
gamma/ °:	120
V/ 10^6 pm^3	1540,80000
Overall displacement parameter:	0,000000
Extinction:	0,000000
Flat Plate Absorption Correction:	0,000000
Porosity:	0,000000
Roughness:	0,000000
Fitting mode:	Structure Fit
U Left:	0,34(2)
V Left:	-0,25(2)
W Left:	0,048(3)
Preferred orientation direction/ hkl:	0,00 0,00 1,00
Preferred orientation parameter:	1,000000
Asymmetry parameter 1:	0,000000
Asymmetry parameter 2:	0,000000
Peak shape:	
parameter 1 Left:	0,63(4)
parameter 2 Left:	0,000000
parameter 3 Left:	0,000000
R (Bragg)/ %:	11,51947

Occupancy, atomic fract. coordinates and Biso for 202834-ICSD, Na3Sc2(PO4)3-HT

Atom	Wyck.	s.o.f.	x	y	z	B/ 10^4 pm^2
Sc1	12c	1,000000	0,000000	0,000000	0,148700	0,000000
P1	18e	1,000000	0,295100	0,000000	0,250000	0,000000
O1	36f	1,000000	0,023500	0,209100	0,194600	0,000000
O2	36f	1,000000	0,192170	0,172830	0,088730	0,000000
Na1	18e	0,347000	0,637200	0,000000	0,250000	0,000000
Na2	6b	0,153000	0,000000	0,000000	0,000000	0,000000

Relevant parameters of 74483-ICSD, ScPO4

Structure and profile data:	
Formula sum:	$Sc_{4,00}P_{4,00}O_{16,00}$
Formula mass/ g/mol:	559,7092
Density (calculated)/ g/cm^3	3,7043
F(000):	272,0000
Weight fraction/ %:	1,4(2)
Space group (No.):	I 41/a m d (141)
Lattice parameters:	
a/ A:	6,579000
b/ A:	6,579000
c/ A:	5,796000
alpha/ °:	90
beta/ °:	90
gamma/ °:	90
V/ 10^6 pm^3	250,86960
Overall displacement parameter:	0,000000
Extinction:	0,000000
Flat Plate Absorption Correction:	0,000000
Porosity:	0,000000
Roughness:	0,000000
Fitting mode:	Structure Fit
U Left:	0,000000
V Left:	0,000000

W Left:	0,010000
Preferred orientation direction/ hkl:	0,00 0,00 1,00
Preferred orientation parameter:	1,000000
Asymmetry parameter 1:	0,000000
Asymmetry parameter 2:	0,000000
Peak shape:	
parameter 1 Left:	0,600000
parameter 2 Left:	0,000000
parameter 3 Left:	0,000000
R (Bragg)/ %:	3,55768

Occupancy, atomic fract. coordinates and Biso for 74483-ICSD, ScPO4

Atom	Wyck.	s.o.f.	x	y	z	B/ 10^4 pm^2
Sc1	4a	1,000000	0,000000	0,750000	0,125000	0,000000
P1	4b	1,000000	0,000000	0,250000	0,375000	0,000000
O1	16h	1,000000	0,000000	0,068900	0,208400	0,378992

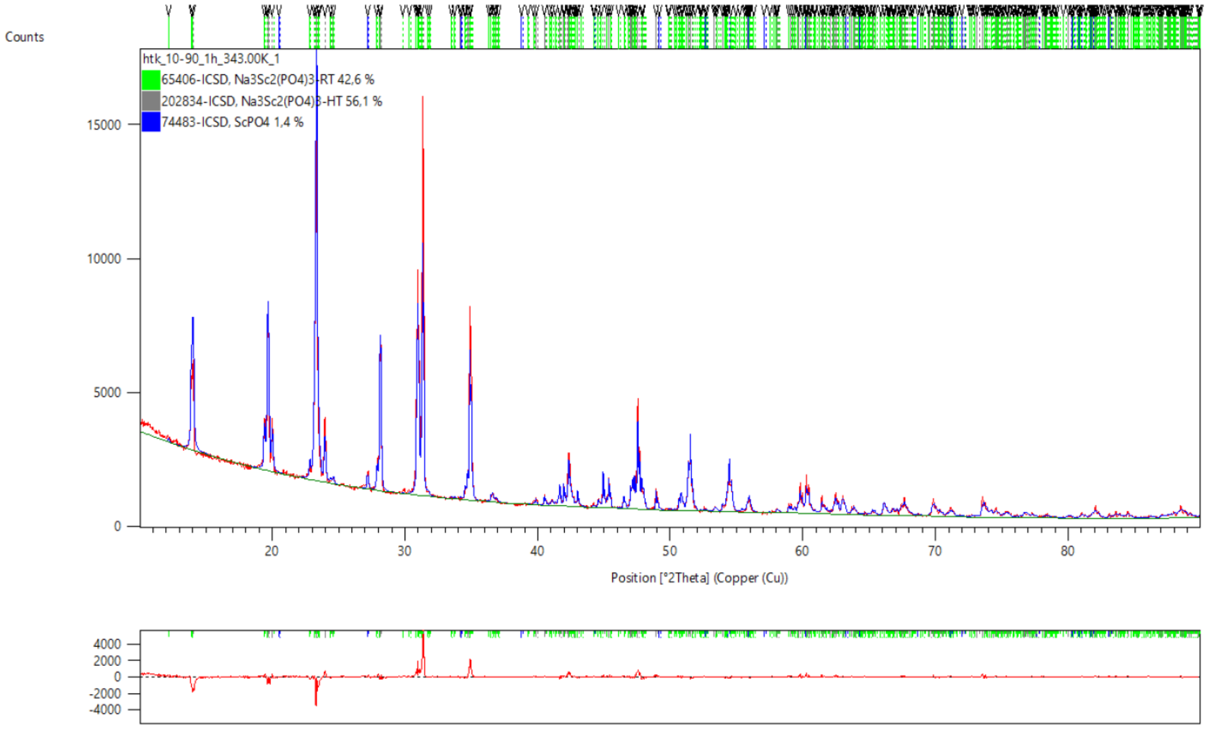

Figure S7. Rietveld refinement of XRD pattern of $Na_3Sc_2(PO_4)_3$:0.2%Eu^{3+} measured at 343K.

$Na_3Sc_2(PO_4)_3$:0.2%Eu^{3+} measured at 353K

Global Parameters

Number of used phases:	3
Number of variables:	15
Number of constraints:	1
Zero shift/ °2Theta:	0,000000
Specimen displacement/ mm :	-0,233(2)
Profile function:	Pseudo Voigt
Background:	Polynomial
R (expected)/ %:	2,81436
R (profile)/ %:	7,28744
R (weighted profile)/ %:	10,79691
GOF:	14,71766

d-statistic:	0,29008
U standard:	0,000000
V standard:	0,000000
W standard:	0,010000
U Left:	0,000000
V Left:	0,000000
W Left:	0,010000
U Right:	0,000000
V Right:	0,000000
W Right:	0,010000
Asymmetry Type:	No Asymmetry Function
Asymmetry 1:	0,000000
Asymmetry 2:	0,000000
Shape Type:	Shape Individual
Shape 1 Left:	0,600000
Shape 2 Left:	0,000000
Shape 3 Left:	0,000000
Shape 1 Right:	0,600000
Shape 2 Right:	0,000000
Shape 3 Right:	0,000000
K a1/a2 intensity ratio:	0,500000
K alpha/beta intensity ratio:	0,000000
Crystal Shape Factor K:	1,0000
Instrumental FWHM Curve Type:	Caglioti function
Instr. Gauss Curve Coefficient A:	0,0045(5)
Instr. Gauss Curve Coefficient B:	-0,0032(9)
Instr. Gauss Curve Coefficient C:	0,0046(3)
Instr. Lorentz Curve Coefficient A:	0,0062(7)
Instr. Lorentz Curve Coefficient B:	-0,004(1)
Instr. Lorentz Curve Coefficient C:	0,0064(5)

Relevant parameters of 65406-ICSD, Na3Sc2(PO4)3-RT

Structure and profile data:	
Formula sum:	$Na_{12,00}P_{12,00}Sc_{8,00}O_{48,00}$
Formula mass/ g/mol:	1775,1820
Density (calculated)/ g/cm^3	2,8300
F(000):	864,0000
Weight fraction/ %:	29(1)
Space group (No.):	C 1 c 1 (9)
Lattice parameters:	
a/ A:	15,392500
b/ A:	8,919392
c/ A:	9,100158
alpha/ °:	90
beta/ °:	123,531000
gamma/ °:	90
V/ 10^6 pm^3	1041,46400
Overall displacement parameter:	0,000000
Extinction:	0,000000
Flat Plate Absorption Correction:	0,000000
Porosity:	0,000000
Roughness:	0,000000
Fitting mode:	Structure Fit
U Left:	0,068212
V Left:	-0,053953
W Left:	0,019893
Preferred orientation direction/ hkl:	0,00 0,00 1,00
Preferred orientation parameter:	1,000000
Asymmetry parameter 1:	0,000000
Asymmetry parameter 2:	0,000000
Peak shape:	
parameter 1 Left:	0,601942
parameter 2 Left:	0,000000
parameter 3 Left:	0,000000
R (Bragg)/ %:	9,60800

Occupancy, atomic fract. coordinates and Biso for 65406-ICSD, Na3Sc2(PO4)3-RT

Atom	Wyck.	s.o.f.	x	y	z	B/ 10^4 pm^2
Na1	4a	1,000000	0,225700	0,151300	0,704900	3,979423
Na2	4a	1,000000	0,422200	0,390700	0,021100	2,787175
Na3	4a	1,000000	0,091500	0,574900	0,453600	2,613470

P1	4a	1,000000	0,060500	0,108400	0,000399	0,813255
P2	4a	1,000000	0,275700	0,608400	0,020400	0,813255
P3	4a	1,000000	0,421700	0,045300	0,012000	0,821151
Sc1	4a	1,000000	0,318200	0,246200	0,207000	0,647446
Sc2	4a	1,000000	0,017100	0,247700	0,309700	0,647446
O1	4a	1,000000	0,272900	0,437200	0,042800	1,073812
O2	4a	1,000000	0,066300	0,061200	0,479800	1,073812
O3	4a	1,000000	0,483100	0,050400	0,676300	1,113291
O4	4a	1,000000	0,386900	0,051000	0,346000	1,113291
O5	4a	1,000000	0,163600	0,181500	0,054300	1,421222
O6	4a	1,000000	0,172000	0,323100	0,472700	1,421222
O7	4a	1,000000	0,035600	0,128600	0,138500	1,934441
O8	4a	1,000000	0,295700	0,366900	0,376300	1,934441
O9	4a	1,000000	0,473400	0,318600	0,325600	1,176456
O10	4a	1,000000	0,361000	0,679200	0,195000	1,176456
O11	4a	1,000000	0,344300	0,149500	0,020800	1,334370
O12	4a	1,000000	0,000000	0,641900	0,000000	1,334370

Relevant parameters of 202834-ICSD, Na3Sc2(PO4)3-HT

Structure and profile data:	
Formula sum:	$Sc_{12,00}P_{18,00}O_{72,00}Na_{7,16}$
Formula mass/ g/mol:	2413,6550
Density (calculated)/ g/cm^3	2,6002
F(000):	1176,8040
Weight fraction/ %:	69(1)
Space group (No.):	R -3 c (167)
Lattice parameters:	
a/ A:	8,9191(4)
b/ A:	8,9191(4)
c/ A:	22,371(1)
alpha/ °:	90
beta/ °:	90
gamma/ °:	120
V/ 10^6 pm^3	1541,16400
Overall displacement parameter:	0,000000
Extinction:	0,000000
Flat Plate Absorption Correction:	0,000000
Porosity:	0,000000
Roughness:	0,000000
Fitting mode:	Structure Fit
U Left:	0,33(2)
V Left:	-0,24(1)
W Left:	0,049(3)
Preferred orientation direction/ hkl:	0,00 0,00 1,00
Preferred orientation parameter:	1,000000
Asymmetry parameter 1:	0,000000
Asymmetry parameter 2:	0,000000
Peak shape:	
parameter 1 Left:	0,54(3)
parameter 2 Left:	0,000000
parameter 3 Left:	0,000000
R (Bragg)/ %:	12,39110

Occupancy, atomic fract. coordinates and Biso for 202834-ICSD, Na3Sc2(PO4)3-HT

Atom	Wyck.	s.o.f.	x	y	z	B/ 10^4 pm^2
Sc1	12c	1,000000	0,000000	0,000000	0,148700	0,000000
P1	18e	1,000000	0,295100	0,000000	0,250000	0,000000
O1	36f	1,000000	0,023500	0,209100	0,194600	0,000000
O2	36f	1,000000	0,192170	0,172830	0,088730	0,000000
Na1	18e	0,347000	0,637200	0,000000	0,250000	0,000000
Na2	6b	0,153000	0,000000	0,000000	0,000000	0,000000

Relevant parameters of 74483-ICSD, ScPO4

Structure and profile data:	
Formula sum:	$Sc_{4,00}P_{4,00}O_{16,00}$
Formula mass/ g/mol:	559,7092
Density (calculated)/ g/cm^3	3,7043
F(000):	272,0000
Weight fraction/ %:	1,5(2)
Space group (No.):	I 41/a m d (141)
Lattice parameters:	
a/ A:	6,579000
b/ A:	6,579000
c/ A:	5,796000
alpha/ °:	90
beta/ °:	90
gamma/ °:	90
V/ 10^6 pm^3	250,86960
Overall displacement parameter:	0,000000
Extinction:	0,000000
Flat Plate Absorption Correction:	0,000000
Porosity:	0,000000
Roughness:	0,000000
Fitting mode:	Structure Fit
U Left:	0,000000
V Left:	0,000000
W Left:	0,010000
Preferred orientation direction/ hkl:	0,00 0,00 1,00
Preferred orientation parameter:	1,000000
Asymmetry parameter 1:	0,000000
Asymmetry parameter 2:	0,000000
Peak shape:	
parameter 1 Left:	0,600000
parameter 2 Left:	0,000000
parameter 3 Left:	0,000000
R (Bragg)/ %:	2,81380

Occupancy, atomic fract. coordinates and Biso for 74483-ICSD, ScPO4

Atom	Wyck.	s.o.f.	x	y	z	B/ 10^4 pm^2
Sc1	4a	1,000000	0,000000	0,750000	0,125000	0,000000
P1	4b	1,000000	0,000000	0,250000	0,375000	0,000000
O1	16h	1,000000	0,000000	0,068900	0,208400	0,378992

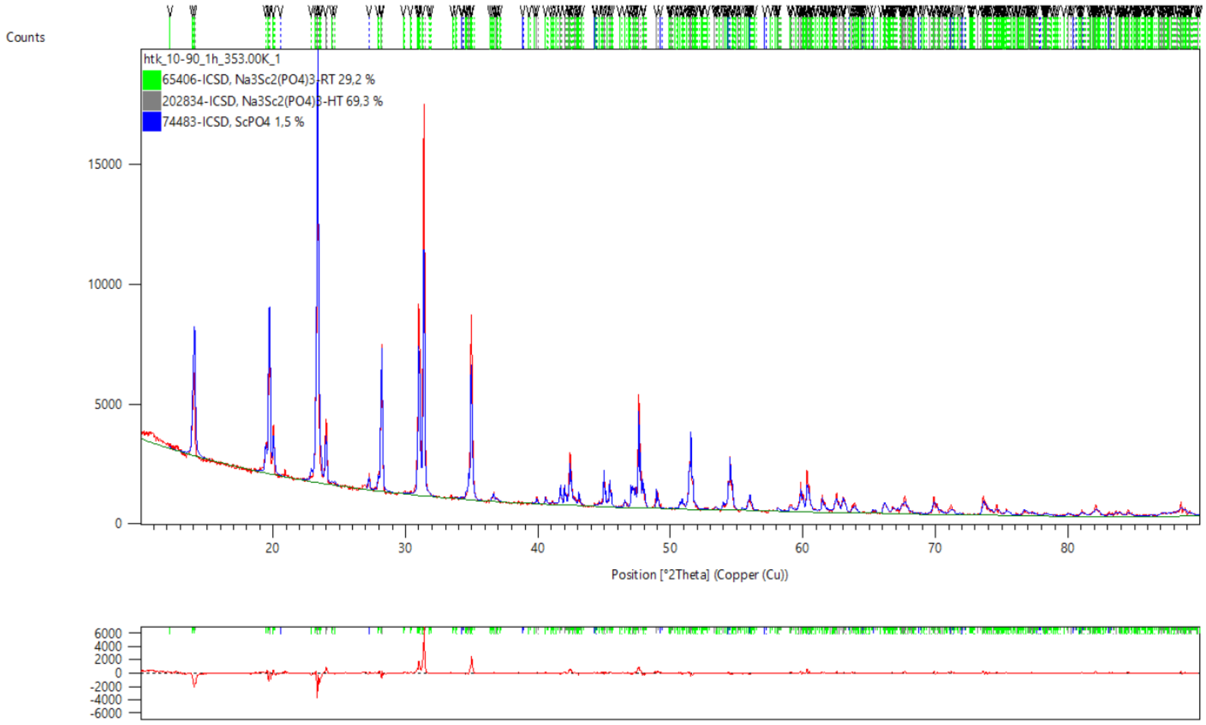

Figure S8. Rietveld refinement of XRD pattern of $Na_3Sc_2(PO_4)_3$:0.2%Eu^{3+} measured at 353K.

$Na_3Sc_2(PO_4)_3$:0.2%Eu^{3+} measured at 363K

Global Parameters

Number of used phases:	2
Number of variables:	14
Number of constraints:	1
Zero shift/ °2Theta:	0,000000
Specimen displacement/ mm :	-0,251(3)
Profile function:	Pseudo Voigt
Background:	Polynomial
R (expected)/ %:	2,82416
R (profile)/ %:	8,44395
R (weighted profile)/ %:	12,53946
GOF:	19,71425
d-statistic:	0,31087
U standard:	0,000000
V standard:	0,000000
W standard:	0,010000
U Left:	0,000000
V Left:	0,000000
W Left:	0,010000
U Right:	0,000000
V Right:	0,000000
W Right:	0,010000
Asymmetry Type:	No Asymmetry Function
Asymmetry 1:	0,000000
Asymmetry 2:	0,000000
Shape Type:	Shape Individual
Shape 1 Left:	0,600000
Shape 2 Left:	0,000000
Shape 3 Left:	0,000000
Shape 1 Right:	0,600000
Shape 2 Right:	0,000000
Shape 3 Right:	0,000000
K a1/a2 intensity ratio:	0,500000
K alpha/beta intensity ratio:	0,000000
Crystal Shape Factor K:	1,0000

Instrumental FWHM Curve Type:	Caglioti function
Instr. Gauss Curve Coefficient A:	0,0045(5)
Instr. Gauss Curve Coefficient B:	-0,0032(9)
Instr. Gauss Curve Coefficient C:	0,0046(3)
Instr. Lorentz Curve Coefficient A:	0,0062(7)
Instr. Lorentz Curve Coefficient B:	-0,004(1)
Instr. Lorentz Curve Coefficient C:	0,0064(5)

Relevant parameters of 202834-ICSD, Na3Sc2(PO4)3-HT

Structure and profile data:	
Formula sum:	$Sc_{12,00}P_{18,00}O_{72,00}Na_{7,16}$
Formula mass/ g/mol:	2413,6550
Density (calculated)/ g/cm^3	2,5985
F(000):	1176,8040
Weight fraction/ %:	98(1)
Space group (No.):	R -3 c (167)
Lattice parameters:	
a/ A:	8,9182(4)
b/ A:	8,9182(4)
c/ A:	22,390(1)
alpha/ °:	90
beta/ °:	90
gamma/ °:	120
V/ 10^6 pm^3	1542,22100
Overall displacement parameter:	0,000000
Extinction:	0,000000
Flat Plate Absorption Correction:	0,000000
Porosity:	0,000000
Roughness:	0,000000
Fitting mode:	Structure Fit
U Left:	0,32(2)
V Left:	-0,23(2)
W Left:	0,047(3)
Preferred orientation direction/ hkl:	0,00 0,00 1,00
Preferred orientation parameter:	1,000000
Asymmetry parameter 1:	0,000000
Asymmetry parameter 2:	0,000000
Peak shape:	
parameter 1 Left:	0,87(3)
parameter 2 Left:	0,000000
parameter 3 Left:	0,000000
R (Bragg)/ %:	12,43119

Occupancy, atomic fract. coordinates and Biso for 202834-ICSD, Na3Sc2(PO4)3-HT

Atom	Wyck.	s.o.f.	x	y	z	B/ 10^4 pm^2
Sc1	12c	1,000000	0,000000	0,000000	0,148700	0,000000
P1	18e	1,000000	0,295100	0,000000	0,250000	0,000000
O1	36f	1,000000	0,023500	0,209100	0,194600	0,000000
O2	36f	1,000000	0,192170	0,172830	0,088730	0,000000
Na1	18e	0,347000	0,637200	0,000000	0,250000	0,000000
Na2	6b	0,153000	0,000000	0,000000	0,000000	0,000000

Relevant parameters of 74483-ICSD, ScPO4

Structure and profile data:	
Formula sum:	$Sc_{4,00}P_{4,00}O_{16,00}$
Formula mass/ g/mol:	559,7092
Density (calculated)/ g/cm^3	3,7043
F(000):	272,0000
Weight fraction/ %:	1,6(2)
Space group (No.):	I 41/a m d (141)
Lattice parameters:	
a/ A:	6,579000
b/ A:	6,579000
c/ A:	5,796000
alpha/ °:	90
beta/ °:	90
gamma/ °:	90
V/ 10^6 pm^3	250,86960

Overall displacement parameter:	0,000000
Extinction:	0,000000
Flat Plate Absorption Correction:	0,000000
Porosity:	0,000000
Roughness:	0,000000
Fitting mode:	Structure Fit
U Left:	0,000000
V Left:	0,000000
W Left:	0,010000
Preferred orientation direction/ hkl:	0,00 0,00 1,00
Preferred orientation parameter:	1,000000
Asymmetry parameter 1:	0,000000
Asymmetry parameter 2:	0,000000
Peak shape:	
parameter 1 Left:	0,600000
parameter 2 Left:	0,000000
parameter 3 Left:	0,000000
R (Bragg)/ %:	4,84985

Occupancy, atomic fract. coordinates and Biso for 74483-ICSD, ScPO4

Atom	Wyck.	s.o.f.	x	y	z	B/ 10^4 pm^2
Sc1	4a	1,000000	0,000000	0,750000	0,125000	0,000000
P1	4b	1,000000	0,000000	0,250000	0,375000	0,000000
O1	16h	1,000000	0,000000	0,068900	0,208400	0,378992

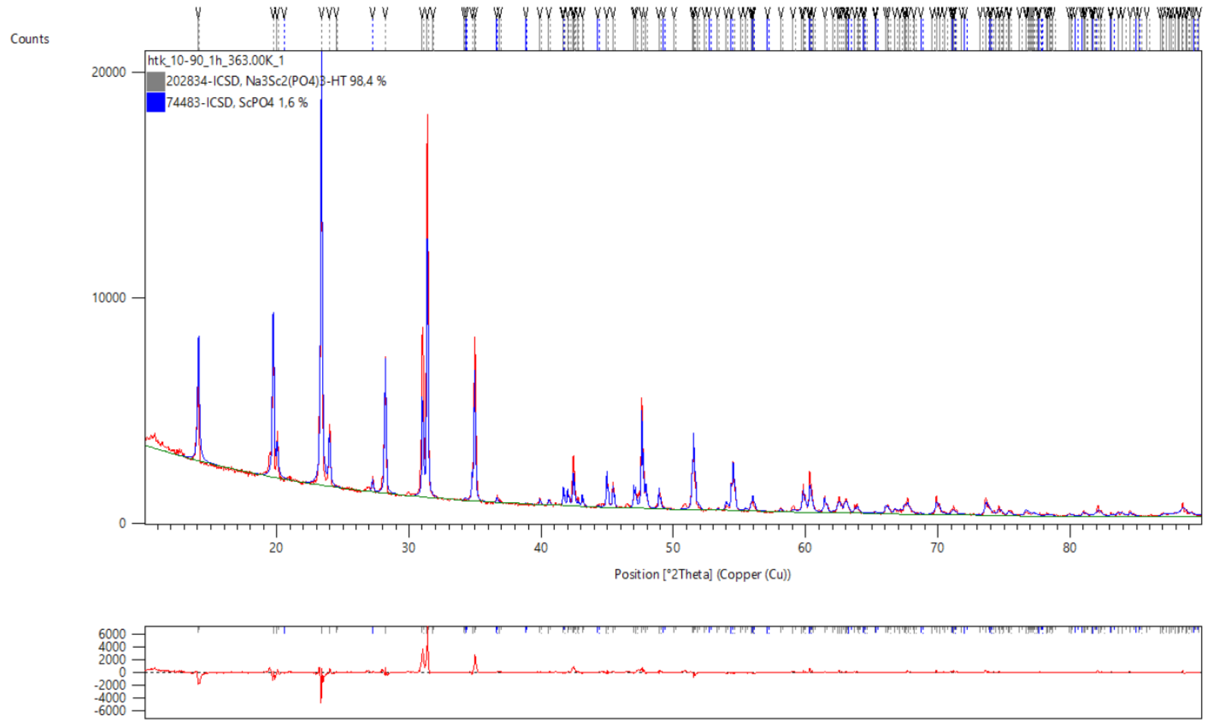

Figure S9. Rietveld refinement of XRD pattern of $Na_3Sc_2(PO_4)_3$:0.2%Eu^{3+} measured at 363K.

$Na_3Sc_2(PO_4)_3$:0.2%Eu^{3+} measured at 373K

Global Parameters

Number of used phases:	2
Number of variables:	14
Number of constraints:	1

Zero shift/ °2Theta:	0,000000
Specimen displacement/ mm :	-0,297(3)
Profile function:	Pseudo Voigt
Background:	Polynomial
R (expected)/ %:	2,82855
R (profile)/ %:	8,21991
R (weighted profile)/ %:	12,29086
GOF:	18,88151
d-statistic:	0,29032
U standard:	0,000000
V standard:	0,000000
W standard:	0,010000
U Left:	0,000000
V Left:	0,000000
W Left:	0,010000
U Right:	0,000000
V Right:	0,000000
W Right:	0,010000
Asymmetry Type:	No Asymmetry Function
Asymmetry 1:	0,000000
Asymmetry 2:	0,000000
Shape Type:	Shape Individual
Shape 1 Left:	0,600000
Shape 2 Left:	0,000000
Shape 3 Left:	0,000000
Shape 1 Right:	0,600000
Shape 2 Right:	0,000000
Shape 3 Right:	0,000000
K a1/a2 intensity ratio:	0,500000
K alpha/beta intensity ratio:	0,000000
Crystal Shape Factor K:	1,0000
Instrumental FWHM Curve Type:	Caglioti function
Instr. Gauss Curve Coefficient A:	0,0045(5)
Instr. Gauss Curve Coefficient B:	-0,0032(9)
Instr. Gauss Curve Coefficient C:	0,0046(3)
Instr. Lorentz Curve Coefficient A:	0,0062(7)
Instr. Lorentz Curve Coefficient B:	-0,004(1)
Instr. Lorentz Curve Coefficient C:	0,0064(5)

Relevant parameters of 202834-ICSD, Na3Sc2(PO4)3-HT

Structure and profile data:	
Formula sum:	$Sc_{12,00}P_{18,00}O_{72,00}Na_{7,16}$
Formula mass/ g/mol:	2413,6550
Density (calculated)/ g/cm^3	2,5969
F(000):	1176,8040
Weight fraction/ %:	98(1)
Space group (No.):	R -3 c (167)
Lattice parameters:	
a/ A:	8,9180(4)
b/ A:	8,9180(4)
c/ A:	22,404(1)
alpha/ °:	90
beta/ °:	90
gamma/ °:	120
V/ 10^6 pm^3	1543,12100
Overall displacement parameter:	0,000000
Extinction:	0,000000
Flat Plate Absorption Correction:	0,000000
Porosity:	0,000000
Roughness:	0,000000
Fitting mode:	Structure Fit
U Left:	0,29(2)
V Left:	-0,20(2)
W Left:	0,043(3)
Preferred orientation direction/ hkl:	0,00 0,00 1,00
Preferred orientation parameter:	1,000000
Asymmetry parameter 1:	0,000000
Asymmetry parameter 2:	0,000000
Peak shape:	
parameter 1 Left:	0,83(3)
parameter 2 Left:	0,000000
parameter 3 Left:	0,000000
R (Bragg)/ %:	12,76872

Occupancy, atomic fract. coordinates and Biso for 202834-ICSD, Na3Sc2(PO4)3-HT

Atom	Wyck.	s.o.f.	x	y	z	B/ 10^4 pm^2
Sc1	12c	1,000000	0,000000	0,000000	0,148700	0,000000
P1	18e	1,000000	0,295100	0,000000	0,250000	0,000000
O1	36f	1,000000	0,023500	0,209100	0,194600	0,000000
O2	36f	1,000000	0,192170	0,172830	0,088730	0,000000
Na1	18e	0,347000	0,637200	0,000000	0,250000	0,000000
Na2	6b	0,153000	0,000000	0,000000	0,000000	0,000000

Relevant parameters of 74483-ICSD, ScPO4

Structure and profile data:	
Formula sum:	$Sc_{4,00}P_{4,00}O_{16,00}$
Formula mass/ g/mol:	559,7092
Density (calculated)/ g/cm^3	3,7043
F(000):	272,0000
Weight fraction/ %:	1,6(2)
Space group (No.):	I 41/a m d (141)
Lattice parameters:	
a/ A:	6,579000
b/ A:	6,579000
c/ A:	5,796000
alpha/ °:	90
beta/ °:	90
gamma/ °:	90
V/ 10^6 pm^3	250,86960
Overall displacement parameter:	0,000000
Extinction:	0,000000
Flat Plate Absorption Correction:	0,000000
Porosity:	0,000000
Roughness:	0,000000
Fitting mode:	Structure Fit
U Left:	0,000000
V Left:	0,000000
W Left:	0,010000
Preferred orientation direction/ hkl:	0,00 0,00 1,00
Preferred orientation parameter:	1,000000
Asymmetry parameter 1:	0,000000
Asymmetry parameter 2:	0,000000
Peak shape:	
parameter 1 Left:	0,600000
parameter 2 Left:	0,000000
parameter 3 Left:	0,000000
R (Bragg)/ %:	4,43308

Occupancy, atomic fract. coordinates and Biso for 74483-ICSD, ScPO4

Atom	Wyck.	s.o.f.	x	y	z	B/ 10^4 pm^2
Sc1	4a	1,000000	0,000000	0,750000	0,125000	0,000000
P1	4b	1,000000	0,000000	0,250000	0,375000	0,000000
O1	16h	1,000000	0,000000	0,068900	0,208400	0,378992

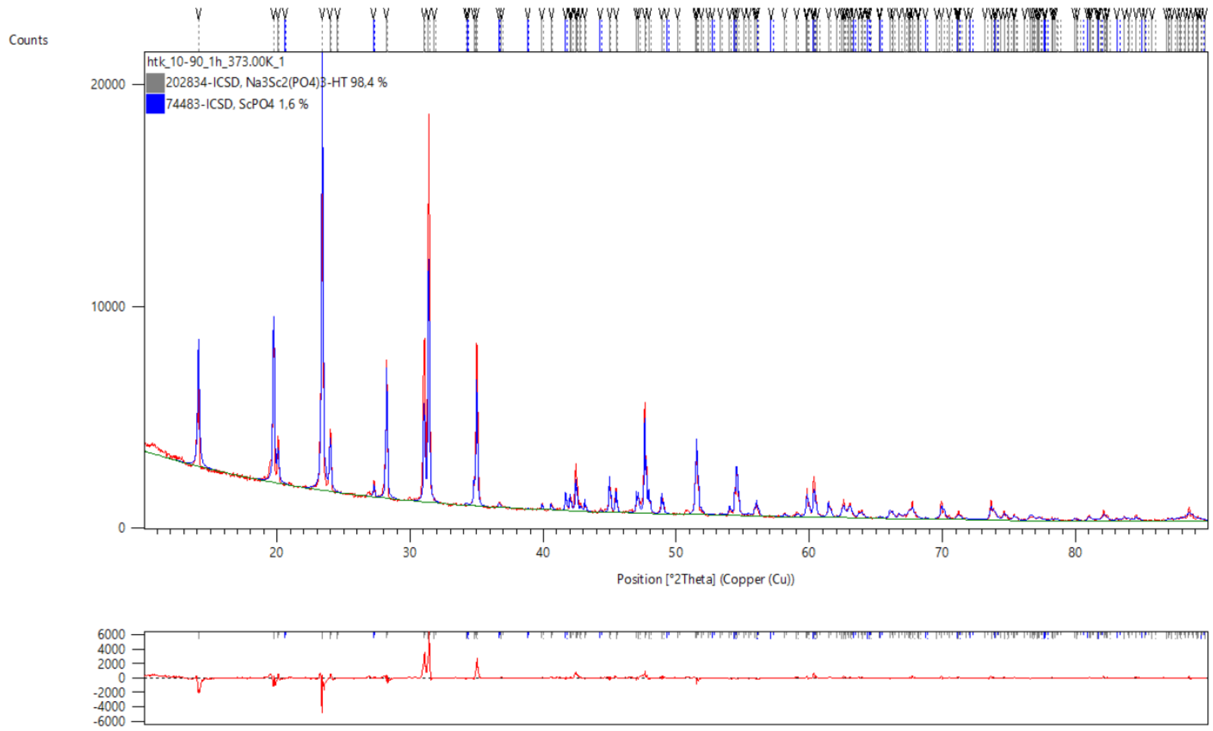

Figure S10. Rietveld refinement of XRD pattern of $Na_3Sc_2(PO_4)_3$:0.2%Eu^{3+} measured at 373K.

$Na_3Sc_2(PO_4)_3$:0.2%Eu^{3+} measured at 383K

Global Parameters

Number of used phases:	2
Number of variables:	14
Number of constraints:	1
Zero shift/ °2Theta:	0,000000
Specimen displacement/ mm :	-0,318(2)
Profile function:	Pseudo Voigt
Background:	Polynomial
R (expected)/ %:	2,82198
R (profile)/ %:	8,12226
R (weighted profile)/ %:	12,30849
GOF:	19,02405
d-statistic:	0,26308
U standard:	0,000000
V standard:	0,000000
W standard:	0,010000
U Left:	0,000000
V Left:	0,000000
W Left:	0,010000
U Right:	0,000000
V Right:	0,000000
W Right:	0,010000
Asymmetry Type:	No Asymmetry Function
Asymmetry 1:	0,000000
Asymmetry 2:	0,000000
Shape Type:	Shape Individual
Shape 1 Left:	0,600000
Shape 2 Left:	0,000000
Shape 3 Left:	0,000000
Shape 1 Right:	0,600000
Shape 2 Right:	0,000000
Shape 3 Right:	0,000000
K a1/a2 intensity ratio:	0,500000
K alpha/beta intensity ratio:	0,000000

Crystal Shape Factor K:	1,0000
Instrumental FWHM Curve Type:	Caglioti function
Instr. Gauss Curve Coefficient A:	0,0045(5)
Instr. Gauss Curve Coefficient B:	-0,0032(9)
Instr. Gauss Curve Coefficient C:	0,0046(3)
Instr. Lorentz Curve Coefficient A:	0,0062(7)
Instr. Lorentz Curve Coefficient B:	-0,004(1)
Instr. Lorentz Curve Coefficient C:	0,0064(5)

Relevant parameters of 202834-ICSD, Na3Sc2(PO4)3-HT

Structure and profile data:	
Formula sum:	$Sc_{12{,}00}P_{18{,}00}O_{72{,}00}Na_{7{,}16}$
Formula mass/ g/mol:	2413,6550
Density (calculated)/ g/cm^3	2,5951
F(000):	1176,8040
Weight fraction/ %:	98(1)
Space group (No.):	R -3 c (167)
Lattice parameters:	
a/ A:	8,9180(4)
b/ A:	8,9180(4)
c/ A:	22,421(1)
alpha/ °:	90
beta/ °:	90
gamma/ °:	120
V/ 10^6 pm^3	1544,23200
Overall displacement parameter:	0,000000
Extinction:	0,000000
Flat Plate Absorption Correction:	0,000000
Porosity:	0,000000
Roughness:	0,000000
Fitting mode:	Structure Fit
U Left:	0,28(2)
V Left:	-0,20(1)
W Left:	0,041(3)
Preferred orientation direction/ hkl:	0,00 0,00 1,00
Preferred orientation parameter:	1,000000
Asymmetry parameter 1:	0,000000
Asymmetry parameter 2:	0,000000
Peak shape:	
parameter 1 Left:	0,79(3)
parameter 2 Left:	0,000000
parameter 3 Left:	0,000000
R (Bragg)/ %:	13,17541

Occupancy, atomic fract. coordinates and Biso for 202834-ICSD, Na3Sc2(PO4)3-HT

Atom	Wyck.	s.o.f.	x	y	z	B/ 10^4 pm^2
Sc1	12c	1,000000	0,000000	0,000000	0,148700	0,000000
P1	18e	1,000000	0,295100	0,000000	0,250000	0,000000
O1	36f	1,000000	0,023500	0,209100	0,194600	0,000000
O2	36f	1,000000	0,192170	0,172830	0,088730	0,000000
Na1	18e	0,347000	0,637200	0,000000	0,250000	0,000000
Na2	6b	0,153000	0,000000	0,000000	0,000000	0,000000

Relevant parameters of 74483-ICSD, ScPO4

Structure and profile data:	
Formula sum:	$Sc_{4{,}00}P_{4{,}00}O_{16{,}00}$
Formula mass/ g/mol:	559,7092
Density (calculated)/ g/cm^3	3,7043
F(000):	272,0000
Weight fraction/ %:	1,6(2)
Space group (No.):	I 41/a m d (141)
Lattice parameters:	
a/ A:	6,579000
b/ A:	6,579000
c/ A:	5,796000
alpha/ °:	90
beta/ °:	90
gamma/ °:	90

V/ 10^6 pm^3	250,86960
Overall displacement parameter:	0,000000
Extinction:	0,000000
Flat Plate Absorption Correction:	0,000000
Porosity:	0,000000
Roughness:	0,000000
Fitting mode:	Structure Fit
U Left:	0,000000
V Left:	0,000000
W Left:	0,010000
Preferred orientation direction/ hkl:	0,00 0,00 1,00
Preferred orientation parameter:	1,000000
Asymmetry parameter 1:	0,000000
Asymmetry parameter 2:	0,000000
Peak shape:	
parameter 1 Left:	0,600000
parameter 2 Left:	0,000000
parameter 3 Left:	0,000000
R (Bragg)/ %:	4,51169

Occupancy, atomic fract. coordinates and Biso for 74483-ICSD, ScPO4

Atom	Wyck.	s.o.f.	x	y	z	B/ 10^4 pm^2
Sc1	4a	1,000000	0,000000	0,750000	0,125000	0,000000
P1	4b	1,000000	0,000000	0,250000	0,375000	0,000000
O1	16h	1,000000	0,000000	0,068900	0,208400	0,378992

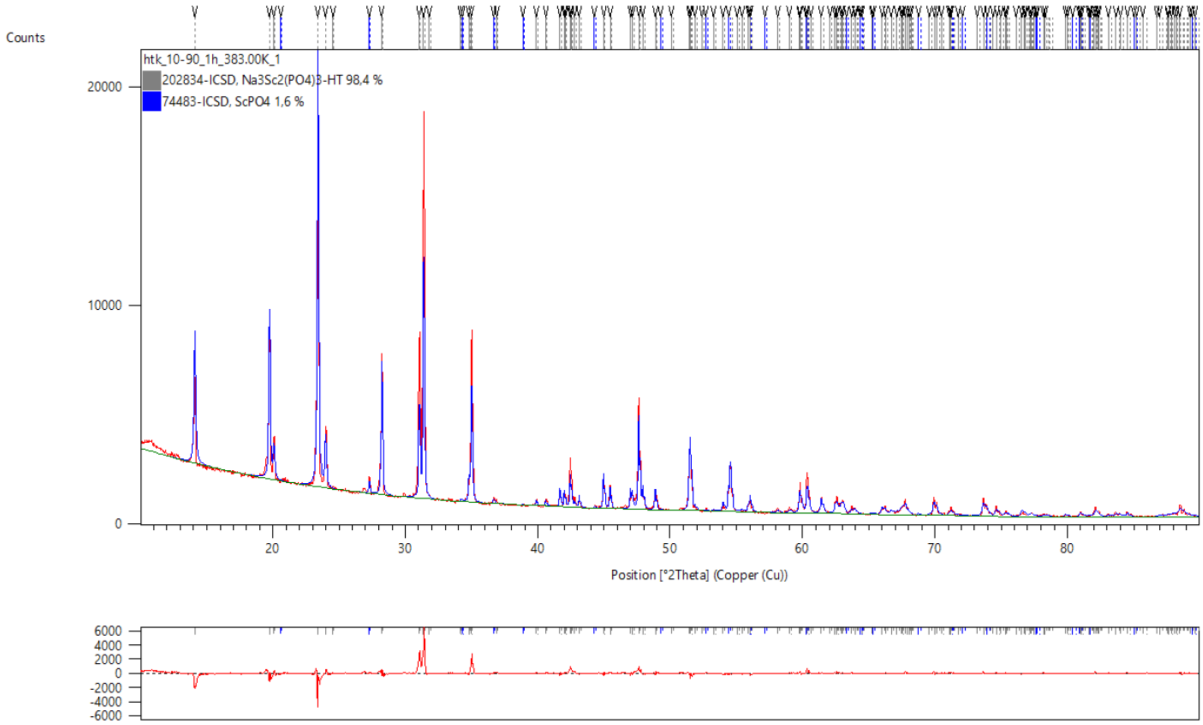

Figure S11. Rietveld refinement of XRD pattern of $Na_3Sc_2(PO_4)_3$:0.2%Eu^{3+} measured at 383K.

$Na_3Sc_2(PO_4)_3$:0.2%Eu^{3+} measured at 393K

Global Parameters

Number of used phases:	2
Number of variables:	14

Number of constraints:	1
Zero shift/ °2Theta:	0,000000
Specimen displacement/ mm :	-0,285(2)
Profile function:	Pseudo Voigt
Background:	Polynomial
R (expected)/ %:	2,83281
R (profile)/ %:	8,05365
R (weighted profile)/ %:	12,20767
GOF:	18,57075
d-statistic:	0,26190
U standard:	0,000000
V standard:	0,000000
W standard:	0,010000
U Left:	0,000000
V Left:	0,000000
W Left:	0,010000
U Right:	0,000000
V Right:	0,000000
W Right:	0,010000
Asymmetry Type:	No Asymmetry Function
Asymmetry 1:	0,000000
Asymmetry 2:	0,000000
Shape Type:	Shape Individual
Shape 1 Left:	0,600000
Shape 2 Left:	0,000000
Shape 3 Left:	0,000000
Shape 1 Right:	0,600000
Shape 2 Right:	0,000000
Shape 3 Right:	0,000000
K a1/a2 intensity ratio:	0,500000
K alpha/beta intensity ratio:	0,000000
Crystal Shape Factor K:	1,0000
Instrumental FWHM Curve Type:	Caglioti function
Instr. Gauss Curve Coefficient A:	0,0045(5)
Instr. Gauss Curve Coefficient B:	-0,0032(9)
Instr. Gauss Curve Coefficient C:	0,0046(3)
Instr. Lorentz Curve Coefficient A:	0,0062(7)
Instr. Lorentz Curve Coefficient B:	-0,004(1)
Instr. Lorentz Curve Coefficient C:	0,0064(5)

Relevant parameters of 202834-ICSD, Na3Sc2(PO4)3-HT

Structure and profile data:	
Formula sum:	$Sc_{12,00}P_{18,00}O_{72,00}Na_{7,16}$
Formula mass/ g/mol:	2413,6550
Density (calculated)/ g/cm^3	2,5927
F(000):	1176,8040
Weight fraction/ %:	98(1)
Space group (No.):	R -3 c (167)
Lattice parameters:	
a/ A:	8,9186(4)
b/ A:	8,9186(4)
c/ A:	22,438(1)
alpha/ °:	90
beta/ °:	90
gamma/ °:	120
V/ 10^6 pm^3	1545,66000
Overall displacement parameter:	0,000000
Extinction:	0,000000
Flat Plate Absorption Correction:	0,000000
Porosity:	0,000000
Roughness:	0,000000
Fitting mode:	Structure Fit
U Left:	0,27(2)
V Left:	-0,19(1)
W Left:	0,040(2)
Preferred orientation direction/ hkl:	0,00 0,00 1,00
Preferred orientation parameter:	1,000000
Asymmetry parameter 1:	0,000000
Asymmetry parameter 2:	0,000000
Peak shape:	
parameter 1 Left:	0,76(3)
parameter 2 Left:	0,000000
parameter 3 Left:	0,000000
R (Bragg)/ %:	13,21702

Occupancy, atomic fract. coordinates and Biso for 202834-ICSD, Na3Sc2(PO4)3-HT

Atom	Wyck.	s.o.f.	x	y	z	B/ 10^4 pm^2
Sc1	12c	1,000000	0,000000	0,000000	0,148700	0,000000
P1	18e	1,000000	0,295100	0,000000	0,250000	0,000000
O1	36f	1,000000	0,023500	0,209100	0,194600	0,000000
O2	36f	1,000000	0,192170	0,172830	0,088730	0,000000
Na1	18e	0,347000	0,637200	0,000000	0,250000	0,000000
Na2	6b	0,153000	0,000000	0,000000	0,000000	0,000000

Relevant parameters of 74483-ICSD, ScPO4

Structure and profile data:	
Formula sum:	$Sc_{4,00}P_{4,00}O_{16,00}$
Formula mass/ g/mol:	559,7092
Density (calculated)/ g/cm^3	3,7043
F(000):	272,0000
Weight fraction/ %:	1,7(2)
Space group (No.):	I 41/a m d (141)
Lattice parameters:	
a/ A:	6,579000
b/ A:	6,579000
c/ A:	5,796000
alpha/ °:	90
beta/ °:	90
gamma/ °:	90
V/ 10^6 pm^3	250,86960
Overall displacement parameter:	0,000000
Extinction:	0,000000
Flat Plate Absorption Correction:	0,000000
Porosity:	0,000000
Roughness:	0,000000
Fitting mode:	Structure Fit
U Left:	0,000000
V Left:	0,000000
W Left:	0,010000
Preferred orientation direction/ hkl:	0,00 0,00 1,00
Preferred orientation parameter:	1,000000
Asymmetry parameter 1:	0,000000
Asymmetry parameter 2:	0,000000
Peak shape:	
parameter 1 Left:	0,600000
parameter 2 Left:	0,000000
parameter 3 Left:	0,000000
R (Bragg)/ %:	3,90381

Occupancy, atomic fract. coordinates and Biso for 74483-ICSD, ScPO4

Atom	Wyck.	s.o.f.	x	y	z	B/ 10^4 pm^2
Sc1	4a	1,000000	0,000000	0,750000	0,125000	0,000000
P1	4b	1,000000	0,000000	0,250000	0,375000	0,000000
O1	16h	1,000000	0,000000	0,068900	0,208400	0,378992

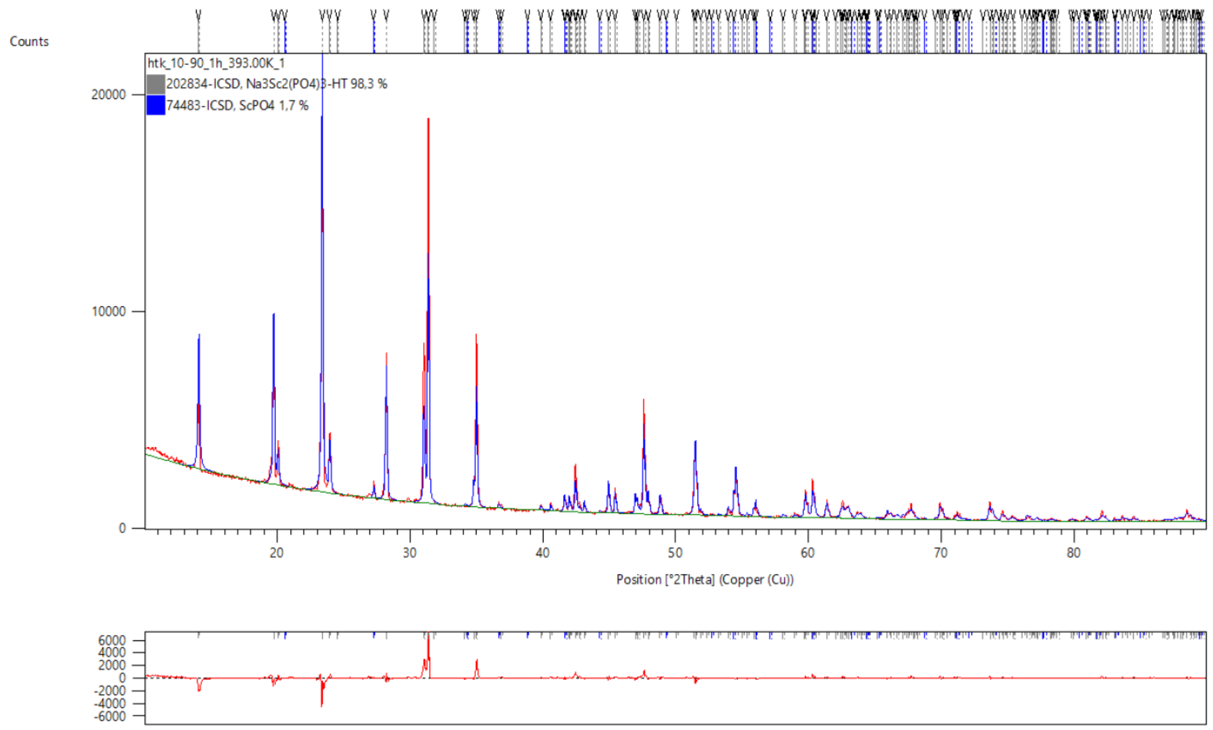

Figure S12. Rietveld refinement of XRD pattern of $Na_3Sc_2(PO_4)_3$:0.2%Eu^{3+} measured at 393K.

$Na_3Sc_2(PO_4)_3$:0.2%Eu^{3+} measured at 403K

Global Parameters

Number of used phases:	2
Number of variables:	14
Number of constraints:	1
Zero shift/ °2Theta:	0,000000
Specimen displacement/ mm :	-0,314(2)
Profile function:	Pseudo Voigt
Background:	Polynomial
R (expected)/ %:	2,83942
R (profile)/ %:	8,07131
R (weighted profile)/ %:	12,15341
GOF:	18,32054
d-statistic:	0,27266
U standard:	0,000000
V standard:	0,000000
W standard:	0,010000
U Left:	0,000000
V Left:	0,000000
W Left:	0,010000
U Right:	0,000000
V Right:	0,000000
W Right:	0,010000
Asymmetry Type:	No Asymmetry Function
Asymmetry 1:	0,000000
Asymmetry 2:	0,000000
Shape Type:	Shape Individual
Shape 1 Left:	0,600000
Shape 2 Left:	0,000000
Shape 3 Left:	0,000000
Shape 1 Right:	0,600000
Shape 2 Right:	0,000000
Shape 3 Right:	0,000000
K a1/a2 intensity ratio:	0,500000
K alpha/beta intensity ratio:	0,000000
Crystal Shape Factor K:	1,0000

Instrumental FWHM Curve Type:	Caglioti function
Instr. Gauss Curve Coefficient A:	0,0045(5)
Instr. Gauss Curve Coefficient B:	-0,0032(9)
Instr. Gauss Curve Coefficient C:	0,0046(3)
Instr. Lorentz Curve Coefficient A:	0,0062(7)
Instr. Lorentz Curve Coefficient B:	-0,004(1)
Instr. Lorentz Curve Coefficient C:	0,0064(5)

Relevant parameters of 202834-ICSD, Na3Sc2(PO4)3-HT

Structure and profile data:	
Formula sum:	$Sc_{12,00}P_{18,00}O_{72,00}Na_{7,16}$
Formula mass/ g/mol:	2413,6550
Density (calculated)/ g/cm^3	2,5912
F(000):	1176,8040
Weight fraction/ %:	98(1)
Space group (No.):	R -3 c (167)
Lattice parameters:	
a/ A:	8,9181(4)
b/ A:	8,9181(4)
c/ A:	22,454(1)
alpha/ °:	90
beta/ °:	90
gamma/ °:	120
V/ 10^6 pm^3	1546,55900
Overall displacement parameter:	0,000000
Extinction:	0,000000
Flat Plate Absorption Correction:	0,000000
Porosity:	0,000000
Roughness:	0,000000
Fitting mode:	Structure Fit
U Left:	0,26(2)
V Left:	-0,18(1)
W Left:	0,039(2)
Preferred orientation direction/ hkl:	0,00 0,00 1,00
Preferred orientation parameter:	1,000000
Asymmetry parameter 1:	0,000000
Asymmetry parameter 2:	0,000000
Peak shape:	
parameter 1 Left:	0,73(3)
parameter 2 Left:	0,000000
parameter 3 Left:	0,000000
R (Bragg)/ %:	13,13214

Occupancy, atomic fract. coordinates and Biso for 202834-ICSD, Na3Sc2(PO4)3-HT

Atom	Wyck.	s.o.f.	x	y	z	B/ 10^4 pm^2
Sc1	12c	1,000000	0,000000	0,000000	0,148700	0,000000
P1	18e	1,000000	0,295100	0,000000	0,250000	0,000000
O1	36f	1,000000	0,023500	0,209100	0,194600	0,000000
O2	36f	1,000000	0,192170	0,172830	0,088730	0,000000
Na1	18e	0,347000	0,637200	0,000000	0,250000	0,000000
Na2	6b	0,153000	0,000000	0,000000	0,000000	0,000000

Relevant parameters of 74483-ICSD, ScPO4

Structure and profile data:	
Formula sum:	$Sc_{4,00}P_{4,00}O_{16,00}$
Formula mass/ g/mol:	559,7092
Density (calculated)/ g/cm^3	3,7043
F(000):	272,0000
Weight fraction/ %:	1,6(2)
Space group (No.):	I 41/a m d (141)
Lattice parameters:	
a/ A:	6,579000
b/ A:	6,579000
c/ A:	5,796000
alpha/ °:	90
beta/ °:	90
gamma/ °:	90
V/ 10^6 pm^3	250,86960

Overall displacement parameter:	0,000000
Extinction:	0,000000
Flat Plate Absorption Correction:	0,000000
Porosity:	0,000000
Roughness:	0,000000
Fitting mode:	Structure Fit
U Left:	0,000000
V Left:	0,000000
W Left:	0,010000
Preferred orientation direction/ hkl:	0,00 0,00 1,00
Preferred orientation parameter:	1,000000
Asymmetry parameter 1:	0,000000
Asymmetry parameter 2:	0,000000
Peak shape:	
parameter 1 Left:	0,600000
parameter 2 Left:	0,000000
parameter 3 Left:	0,000000
R (Bragg)/ %:	4,02681

Occupancy, atomic fract. coordinates and Biso for 74483-ICSD, ScPO4

Atom	Wyck.	s.o.f.	x	y	z	B/ 10^4 pm^2
Sc1	4a	1,000000	0,000000	0,750000	0,125000	0,000000
P1	4b	1,000000	0,000000	0,250000	0,375000	0,000000
O1	16h	1,000000	0,000000	0,068900	0,208400	0,378992

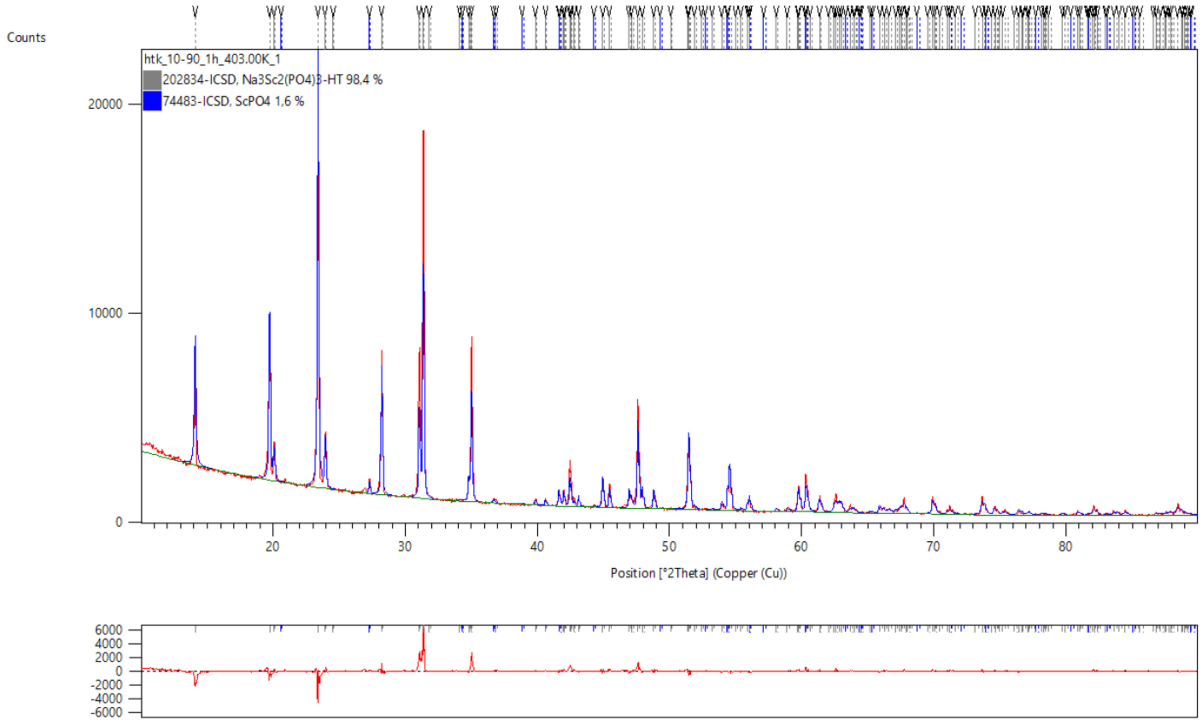

Figure S13. Rietveld refinement of XRD pattern of $Na_3Sc_2(PO_4)_3$:0.2%Eu^{3+} measured at 403K.

$Na_3Sc_2(PO_4)_3$:0.2%Eu^{3+} measured at 413K

Global Parameters

Number of used phases:	2
Number of variables:	14
Number of constraints:	1

Zero shift/ °2Theta:	0,000000
Specimen displacement/ mm :	-0,034(2)
Profile function:	Pseudo Voigt
Background:	Polynomial
R (expected)/ %:	2,84579
R (profile)/ %:	8,13413
R (weighted profile)/ %:	12,38800
GOF:	18,94941
d-statistic:	0,31022
U standard:	0,000000
V standard:	0,000000
W standard:	0,010000
U Left:	0,000000
V Left:	0,000000
W Left:	0,010000
U Right:	0,000000
V Right:	0,000000
W Right:	0,010000
Asymmetry Type:	No Asymmetry Function
Asymmetry 1:	0,000000
Asymmetry 2:	0,000000
Shape Type:	Shape Individual
Shape 1 Left:	0,600000
Shape 2 Left:	0,000000
Shape 3 Left:	0,000000
Shape 1 Right:	0,600000
Shape 2 Right:	0,000000
Shape 3 Right:	0,000000
K a1/a2 intensity ratio:	0,500000
K alpha/beta intensity ratio:	0,000000
Crystal Shape Factor K:	1,0000
Instrumental FWHM Curve Type:	Caglioti function
Instr. Gauss Curve Coefficient A:	0,0045(5)
Instr. Gauss Curve Coefficient B:	-0,0032(9)
Instr. Gauss Curve Coefficient C:	0,0046(3)
Instr. Lorentz Curve Coefficient A:	0,0062(7)
Instr. Lorentz Curve Coefficient B:	-0,004(1)
Instr. Lorentz Curve Coefficient C:	0,0064(5)

Relevant parameters of 202834-ICSD, Na3Sc2(PO4)3-HT

Structure and profile data:	
Formula sum:	$Sc_{12,00}P_{18,00}O_{72,00}Na_{7,16}$
Formula mass/ g/mol:	2413,6550
Density (calculated)/ g/cm^3	2,5861
F(000):	1176,8040
Weight fraction/ %:	98(1)
Space group (No.):	R -3 c (167)
Lattice parameters:	
a/ A:	8,9215(4)
b/ A:	8,9215(4)
c/ A:	22,481(1)
alpha/ °:	90
beta/ °:	90
gamma/ °:	120
V/ 10^6 pm^3	1549,59000
Overall displacement parameter:	0,000000
Extinction:	0,000000
Flat Plate Absorption Correction:	0,000000
Porosity:	0,000000
Roughness:	0,000000
Fitting mode:	Structure Fit
U Left:	0,29(2)
V Left:	-0,20(1)
W Left:	0,040(2)
Preferred orientation direction/ hkl:	0,00 0,00 1,00
Preferred orientation parameter:	1,000000
Asymmetry parameter 1:	0,000000
Asymmetry parameter 2:	0,000000
Peak shape:	
parameter 1 Left:	0,72(3)
parameter 2 Left:	0,000000
parameter 3 Left:	0,000000
R (Bragg)/ %:	13,08202

Occupancy, atomic fract. coordinates and Biso for 202834-ICSD, Na3Sc2(PO4)3-HT

Atom	Wyck.	s.o.f.	x	y	z	B/ 10^4 pm^2
Sc1	12c	1,000000	0,000000	0,000000	0,148700	0,000000
P1	18e	1,000000	0,295100	0,000000	0,250000	0,000000
O1	36f	1,000000	0,023500	0,209100	0,194600	0,000000
O2	36f	1,000000	0,192170	0,172830	0,088730	0,000000
Na1	18e	0,347000	0,637200	0,000000	0,250000	0,000000
Na2	6b	0,153000	0,000000	0,000000	0,000000	0,000000

Relevant parameters of 74483-ICSD, ScPO4

Structure and profile data:	
Formula sum:	$Sc_{4,00}P_{4,00}O_{16,00}$
Formula mass/ g/mol:	559,7092
Density (calculated)/ g/cm^3	3,7043
F(000):	272,0000
Weight fraction/ %:	1,8(2)
Space group (No.):	I 41/a m d (141)
Lattice parameters:	
a/ A:	6,579000
b/ A:	6,579000
c/ A:	5,796000
alpha/ °:	90
beta/ °:	90
gamma/ °:	90
V/ 10^6 pm^3	250,86960
Overall displacement parameter:	0,000000
Extinction:	0,000000
Flat Plate Absorption Correction:	0,000000
Porosity:	0,000000
Roughness:	0,000000
Fitting mode:	Structure Fit
U Left:	0,000000
V Left:	0,000000
W Left:	0,010000
Preferred orientation direction/ hkl:	0,00 0,00 1,00
Preferred orientation parameter:	1,000000
Asymmetry parameter 1:	0,000000
Asymmetry parameter 2:	0,000000
Peak shape:	
parameter 1 Left:	0,600000
parameter 2 Left:	0,000000
parameter 3 Left:	0,000000
R (Bragg)/ %:	3,77083

Occupancy, atomic fract. coordinates and Biso for 74483-ICSD, ScPO4

Atom	Wyck.	s.o.f.	x	y	z	B/ 10^4 pm^2
Sc1	4a	1,000000	0,000000	0,750000	0,125000	0,000000
P1	4b	1,000000	0,000000	0,250000	0,375000	0,000000
O1	16h	1,000000	0,000000	0,068900	0,208400	0,378992

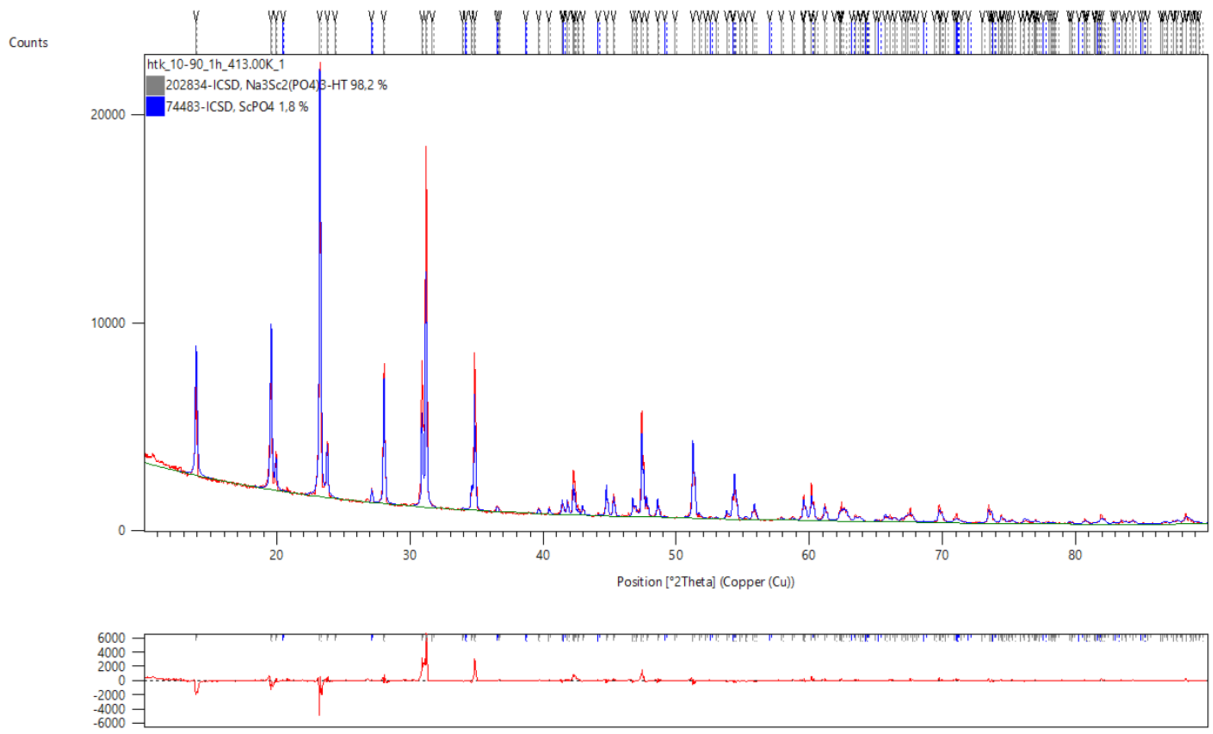

Figure S14. Rietveld refinement of XRD pattern of $Na_3Sc_2(PO_4)_3$:0.2%Eu^{3+} measured at 413K.

$Na_3Sc_2(PO_4)_3$:0.2%Eu^{3+} measured at 423K

Global Parameters

Number of used phases:	2
Number of variables:	14
Number of constraints:	1
Zero shift/ °2Theta:	0,000000
Specimen displacement/ mm :	-0,144(2)
Profile function:	Pseudo Voigt
Background:	Polynomial
R (expected)/ %:	2,84034
R (profile)/ %:	8,20263
R (weighted profile)/ %:	12,53729
GOF:	19,48355
d-statistic:	0,26485
U standard:	0,000000
V standard:	0,000000
W standard:	0,010000
U Left:	0,000000
V Left:	0,000000
W Left:	0,010000
U Right:	0,000000
V Right:	0,000000
W Right:	0,010000
Asymmetry Type:	No Asymmetry Function
Asymmetry 1:	0,000000
Asymmetry 2:	0,000000
Shape Type:	Shape Individual
Shape 1 Left:	0,600000
Shape 2 Left:	0,000000
Shape 3 Left:	0,000000
Shape 1 Right:	0,600000
Shape 2 Right:	0,000000
Shape 3 Right:	0,000000
K a1/a2 intensity ratio:	0,500000
K alpha/beta intensity ratio:	0,000000

Crystal Shape Factor K:	1,0000
Instrumental FWHM Curve Type:	Caglioti function
Instr. Gauss Curve Coefficient A:	0,0045(5)
Instr. Gauss Curve Coefficient B:	-0,0032(9)
Instr. Gauss Curve Coefficient C:	0,0046(3)
Instr. Lorentz Curve Coefficient A:	0,0062(7)
Instr. Lorentz Curve Coefficient B:	-0,004(1)
Instr. Lorentz Curve Coefficient C:	0,0064(5)

Relevant parameters of 202834-ICSD, Na3Sc2(PO4)3-HT

Structure and profile data:	
Formula sum:	$Sc_{12,00}P_{18,00}O_{72,00}Na_{7,16}$
Formula mass/ g/mol:	2413,6550
Density (calculated)/ g/cm^3	2,5843
F(000):	1176,8040
Weight fraction/ %:	98(1)
Space group (No.):	R -3 c (167)
Lattice parameters:	
a/ A:	8,9199(4)
b/ A:	8,9199(4)
c/ A:	22,504(1)
alpha/ °:	90
beta/ °:	90
gamma/ °:	120
V/ 10^6 pm^3	1550,66500
Overall displacement parameter:	0,000000
Extinction:	0,000000
Flat Plate Absorption Correction:	0,000000
Porosity:	0,000000
Roughness:	0,000000
Fitting mode:	Structure Fit
U Left:	0,29(2)
V Left:	-0,19(2)
W Left:	0,041(3)
Preferred orientation direction/ hkl:	0,00 0,00 1,00
Preferred orientation parameter:	1,000000
Asymmetry parameter 1:	0,000000
Asymmetry parameter 2:	0,000000
Peak shape:	
parameter 1 Left:	0,68(3)
parameter 2 Left:	0,000000
parameter 3 Left:	0,000000
R (Bragg)/ %:	13,31618

Occupancy, atomic fract. coordinates and Biso for 202834-ICSD, Na3Sc2(PO4)3-HT

Atom	Wyck.	s.o.f.	x	y	z	B/ 10^4 pm^2
Sc1	12c	1,000000	0,000000	0,000000	0,148700	0,000000
P1	18e	1,000000	0,295100	0,000000	0,250000	0,000000
O1	36f	1,000000	0,023500	0,209100	0,194600	0,000000
O2	36f	1,000000	0,192170	0,172830	0,088730	0,000000
Na1	18e	0,347000	0,637200	0,000000	0,250000	0,000000
Na2	6b	0,153000	0,000000	0,000000	0,000000	0,000000

Relevant parameters of 74483-ICSD, ScPO4

Structure and profile data:	
Formula sum:	$Sc_{4,00}P_{4,00}O_{16,00}$
Formula mass/ g/mol:	559,7092
Density (calculated)/ g/cm^3	3,7043
F(000):	272,0000
Weight fraction/ %:	1,8(2)
Space group (No.):	I 41/a m d (141)
Lattice parameters:	
a/ A:	6,579000
b/ A:	6,579000
c/ A:	5,796000
alpha/ °:	90
beta/ °:	90
gamma/ °:	90

V/ 10^6 pm^3	250,86960
Overall displacement parameter:	0,000000
Extinction:	0,000000
Flat Plate Absorption Correction:	0,000000
Porosity:	0,000000
Roughness:	0,000000
Fitting mode:	Structure Fit
U Left:	0,000000
V Left:	0,000000
W Left:	0,010000
Preferred orientation direction/ hkl:	0,00 0,00 1,00
Preferred orientation parameter:	1,000000
Asymmetry parameter 1:	0,000000
Asymmetry parameter 2:	0,000000
Peak shape:	
parameter 1 Left:	0,600000
parameter 2 Left:	0,000000
parameter 3 Left:	0,000000
R (Bragg)/ %:	3,69443

Occupancy, atomic fract. coordinates and Biso for 74483-ICSD, ScPO4

Atom	Wyck.	s.o.f.	x	y	z	B/ 10^4 pm^2
Sc1	4a	1,000000	0,000000	0,750000	0,125000	0,000000
P1	4b	1,000000	0,000000	0,250000	0,375000	0,000000
O1	16h	1,000000	0,000000	0,068900	0,208400	0,378992

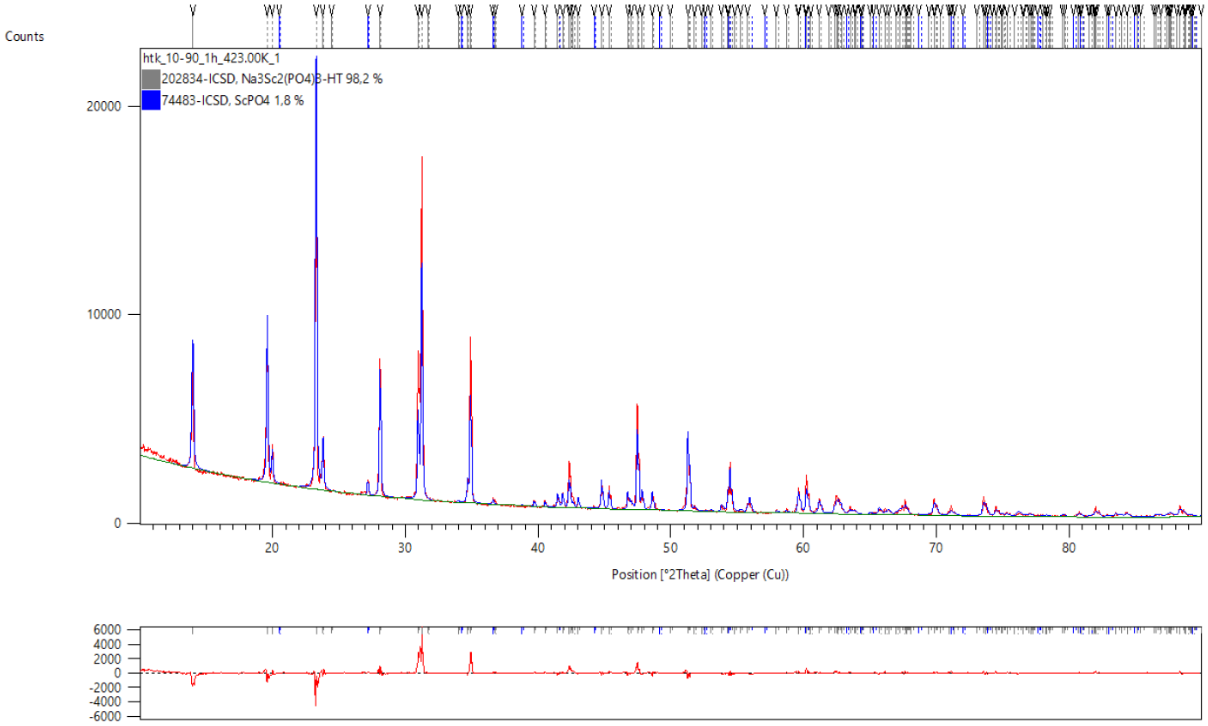

Figure S15. Rietveld refinement of XRD pattern of $Na_3Sc_2(PO_4)_3$:0.2%Eu^{3+} measured at 423K.

$Na_3Sc_2(PO_4)_3$:0.2%Eu^{3+} measured at 433K

Global Parameters

Number of used phases:	2
Number of variables:	14

Number of constraints:	1
Zero shift/ °2Theta:	0,000000
Specimen displacement/ mm :	-0,196(2)
Profile function:	Pseudo Voigt
Background:	Polynomial
R (expected)/ %:	2,84459
R (profile)/ %:	7,91001
R (weighted profile)/ %:	12,18764
GOF:	18,35693
d-statistic:	0,23455
U standard:	0,000000
V standard:	0,000000
W standard:	0,010000
U Left:	0,000000
V Left:	0,000000
W Left:	0,010000
U Right:	0,000000
V Right:	0,000000
W Right:	0,010000
Asymmetry Type:	No Asymmetry Function
Asymmetry 1:	0,000000
Asymmetry 2:	0,000000
Shape Type:	Shape Individual
Shape 1 Left:	0,600000
Shape 2 Left:	0,000000
Shape 3 Left:	0,000000
Shape 1 Right:	0,600000
Shape 2 Right:	0,000000
Shape 3 Right:	0,000000
K a1/a2 intensity ratio:	0,500000
K alpha/beta intensity ratio:	0,000000
Crystal Shape Factor K:	1,0000
Instrumental FWHM Curve Type:	Caglioti function
Instr. Gauss Curve Coefficient A:	0,0045(5)
Instr. Gauss Curve Coefficient B:	-0,0032(9)
Instr. Gauss Curve Coefficient C:	0,0046(3)
Instr. Lorentz Curve Coefficient A:	0,0062(7)
Instr. Lorentz Curve Coefficient B:	-0,004(1)
Instr. Lorentz Curve Coefficient C:	0,0064(5)

Relevant parameters of 202834-ICSD, Na3Sc2(PO4)3-HT

Structure and profile data:	
Formula sum:	$Sc_{12{,}00}P_{18{,}00}O_{72{,}00}Na_{7{,}16}$
Formula mass/ g/mol:	2413,6550
Density (calculated)/ g/cm^3	2,5793
F(000):	1176,8040
Weight fraction/ %:	98(1)
Space group (No.):	R -3 c (167)
Lattice parameters:	
a/ A:	8,9196(4)
b/ A:	8,9196(4)
c/ A:	22,549(1)
alpha/ °:	90
beta/ °:	90
gamma/ °:	120
V/ 10^6 pm^3	1553,66900
Overall displacement parameter:	0,000000
Extinction:	0,000000
Flat Plate Absorption Correction:	0,000000
Porosity:	0,000000
Roughness:	0,000000
Fitting mode:	Structure Fit
U Left:	0,25(2)
V Left:	-0,17(1)
W Left:	0,036(2)
Preferred orientation direction/ hkl:	0,00 0,00 1,00
Preferred orientation parameter:	1,000000
Asymmetry parameter 1:	0,000000
Asymmetry parameter 2:	0,000000
Peak shape:	
parameter 1 Left:	0,68(3)
parameter 2 Left:	0,000000
parameter 3 Left:	0,000000
R (Bragg)/ %:	13,49347

Occupancy, atomic fract. coordinates and Biso for 202834-ICSD, Na3Sc2(PO4)3-HT

Atom	Wyck.	s.o.f.	x	y	z	B/ 10^4 pm^2
Sc1	12c	1,000000	0,000000	0,000000	0,148700	0,000000
P1	18e	1,000000	0,295100	0,000000	0,250000	0,000000
O1	36f	1,000000	0,023500	0,209100	0,194600	0,000000
O2	36f	1,000000	0,192170	0,172830	0,088730	0,000000
Na1	18e	0,347000	0,637200	0,000000	0,250000	0,000000
Na2	6b	0,153000	0,000000	0,000000	0,000000	0,000000

Relevant parameters of 74483-ICSD, ScPO4

Structure and profile data:	
Formula sum:	$Sc_{4,00}P_{4,00}O_{16,00}$
Formula mass/ g/mol:	559,7092
Density (calculated)/ g/cm^3	3,7043
F(000):	272,0000
Weight fraction/ %:	1,8(2)
Space group (No.):	I 41/a m d (141)
Lattice parameters:	
a/ A:	6,579000
b/ A:	6,579000
c/ A:	5,796000
alpha/ °:	90
beta/ °:	90
gamma/ °:	90
V/ 10^6 pm^3	250,86960
Overall displacement parameter:	0,000000
Extinction:	0,000000
Flat Plate Absorption Correction:	0,000000
Porosity:	0,000000
Roughness:	0,000000
Fitting mode:	Structure Fit
U Left:	0,000000
V Left:	0,000000
W Left:	0,010000
Preferred orientation direction/ hkl:	0,00 0,00 1,00
Preferred orientation parameter:	1,000000
Asymmetry parameter 1:	0,000000
Asymmetry parameter 2:	0,000000
Peak shape:	
parameter 1 Left:	0,600000
parameter 2 Left:	0,000000
parameter 3 Left:	0,000000
R (Bragg)/ %:	3,66073

Occupancy, atomic fract. coordinates and Biso for 74483-ICSD, ScPO4

Atom	Wyck.	s.o.f.	x	y	z	B/ 10^4 pm^2
Sc1	4a	1,000000	0,000000	0,750000	0,125000	0,000000
P1	4b	1,000000	0,000000	0,250000	0,375000	0,000000
O1	16h	1,000000	0,000000	0,068900	0,208400	0,378992

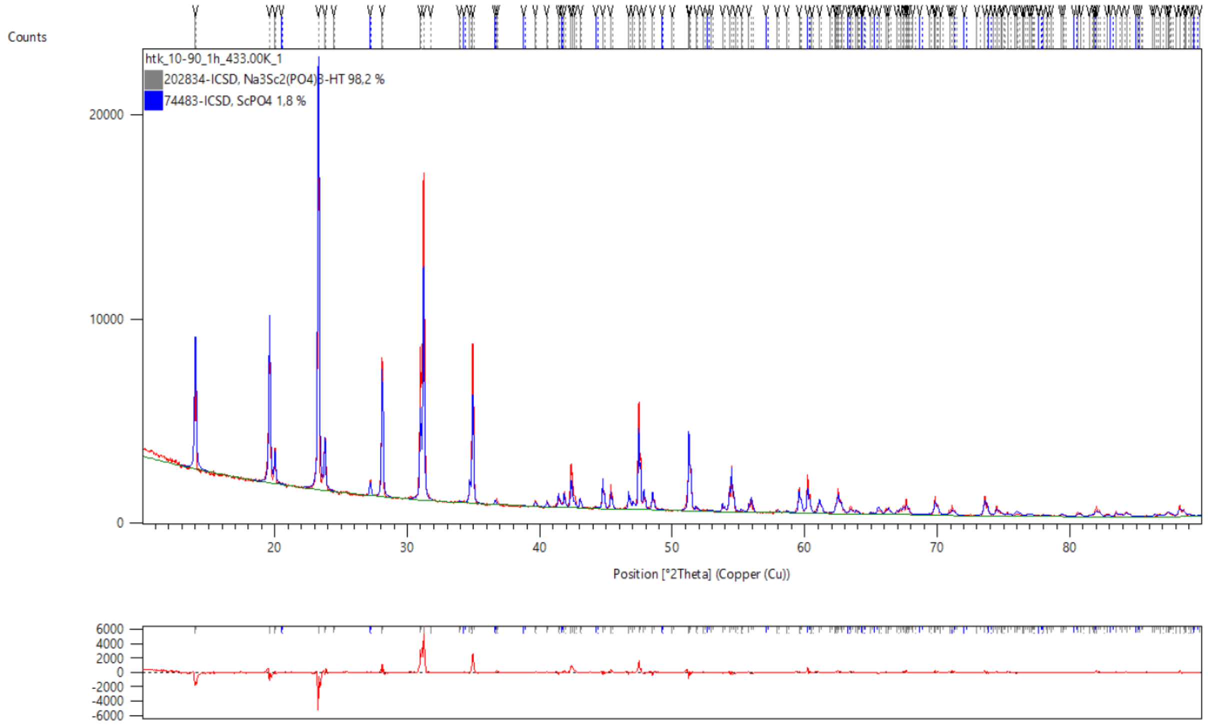

Figure S16. Rietveld refinement of XRD pattern of $Na_3Sc_2(PO_4)_3$:0.2%Eu^{3+} measured at 433K.

$Na_3Sc_2(PO_4)_3$:0.2%Eu^{3+} measured at 443K

Global Parameters

Number of used phases:	2
Number of variables:	14
Number of constraints:	1
Zero shift/ °2Theta:	0,000000
Specimen displacement/ mm :	-0,208(2)
Profile function:	Pseudo Voigt
Background:	Polynomial
R (expected)/ %:	2,84203
R (profile)/ %:	7,99763
R (weighted profile)/ %:	12,33102
GOF:	18,82527
d-statistic:	0,28452
U standard:	0,000000
V standard:	0,000000
W standard:	0,010000
U Left:	0,000000
V Left:	0,000000
W Left:	0,010000
U Right:	0,000000
V Right:	0,000000
W Right:	0,010000
Asymmetry Type:	No Asymmetry Function
Asymmetry 1:	0,000000
Asymmetry 2:	0,000000
Shape Type:	Shape Individual
Shape 1 Left:	0,600000
Shape 2 Left:	0,000000
Shape 3 Left:	0,000000
Shape 1 Right:	0,600000
Shape 2 Right:	0,000000
Shape 3 Right:	0,000000
K a1/a2 intensity ratio:	0,500000
K alpha/beta intensity ratio:	0,000000
Crystal Shape Factor K:	1,0000

Instrumental FWHM Curve Type:	Caglioti function
Instr. Gauss Curve Coefficient A:	0,0045(5)
Instr. Gauss Curve Coefficient B:	-0,0032(9)
Instr. Gauss Curve Coefficient C:	0,0046(3)
Instr. Lorentz Curve Coefficient A:	0,0062(7)
Instr. Lorentz Curve Coefficient B:	-0,004(1)
Instr. Lorentz Curve Coefficient C:	0,0064(5)

Relevant parameters of 202834-ICSD, Na3Sc2(PO4)3-HT

Structure and profile data:	
Formula sum:	$Sc_{12,00}P_{18,00}O_{72,00}Na_{7,16}$
Formula mass/ g/mol:	2413,6550
Density (calculated)/ g/cm^3	2,5776
F(000):	1176,8040
Weight fraction/ %:	98(1)
Space group (No.):	R -3 c (167)
Lattice parameters:	
a/ A:	8,9190(4)
b/ A:	8,9190(4)
c/ A:	22,568(1)
alpha/ °:	90
beta/ °:	90
gamma/ °:	120
V/ 10^6 pm^3	1554,73100
Overall displacement parameter:	0,000000
Extinction:	0,000000
Flat Plate Absorption Correction:	0,000000
Porosity:	0,000000
Roughness:	0,000000
Fitting mode:	Structure Fit
U Left:	0,23(2)
V Left:	-0,16(1)
W Left:	0,035(2)
Preferred orientation direction/ hkl:	0,00 0,00 1,00
Preferred orientation parameter:	1,000000
Asymmetry parameter 1:	0,000000
Asymmetry parameter 2:	0,000000
Peak shape:	
parameter 1 Left:	0,65(3)
parameter 2 Left:	0,000000
parameter 3 Left:	0,000000
R (Bragg)/ %:	13,78730

Occupancy, atomic fract. coordinates and Biso for 202834-ICSD, Na3Sc2(PO4)3-HT

Atom	Wyck.	s.o.f.	x	y	z	B/ 10^4 pm^2
Sc1	12c	1,000000	0,000000	0,000000	0,148700	0,000000
P1	18e	1,000000	0,295100	0,000000	0,250000	0,000000
O1	36f	1,000000	0,023500	0,209100	0,194600	0,000000
O2	36f	1,000000	0,192170	0,172830	0,088730	0,000000
Na1	18e	0,347000	0,637200	0,000000	0,250000	0,000000
Na2	6b	0,153000	0,000000	0,000000	0,000000	0,000000

Relevant parameters of 74483-ICSD, ScPO4

Structure and profile data:	
Formula sum:	$Sc_{4,00}P_{4,00}O_{16,00}$
Formula mass/ g/mol:	559,7092
Density (calculated)/ g/cm^3	3,7043
F(000):	272,0000
Weight fraction/ %:	1,8(2)
Space group (No.):	I 41/a m d (141)
Lattice parameters:	
a/ A:	6,579000
b/ A:	6,579000
c/ A:	5,796000
alpha/ °:	90
beta/ °:	90
gamma/ °:	90
V/ 10^6 pm^3	250,86960

Overall displacement parameter:	0,000000
Extinction:	0,000000
Flat Plate Absorption Correction:	0,000000
Porosity:	0,000000
Roughness:	0,000000
Fitting mode:	Structure Fit
U Left:	0,000000
V Left:	0,000000
W Left:	0,010000
Preferred orientation direction/ hkl:	0,00 0,00 1,00
Preferred orientation parameter:	1,000000
Asymmetry parameter 1:	0,000000
Asymmetry parameter 2:	0,000000
Peak shape:	
parameter 1 Left:	0,600000
parameter 2 Left:	0,000000
parameter 3 Left:	0,000000
R (Bragg)/ %:	3,95353

Occupancy, atomic fract. coordinates and Biso for 74483-ICSD, ScPO4

Atom	Wyck.	s.o.f.	x	y	z	B/ 10^4 pm^2
Sc1	4a	1,000000	0,000000	0,750000	0,125000	0,000000
P1	4b	1,000000	0,000000	0,250000	0,375000	0,000000
O1	16h	1,000000	0,000000	0,068900	0,208400	0,378992

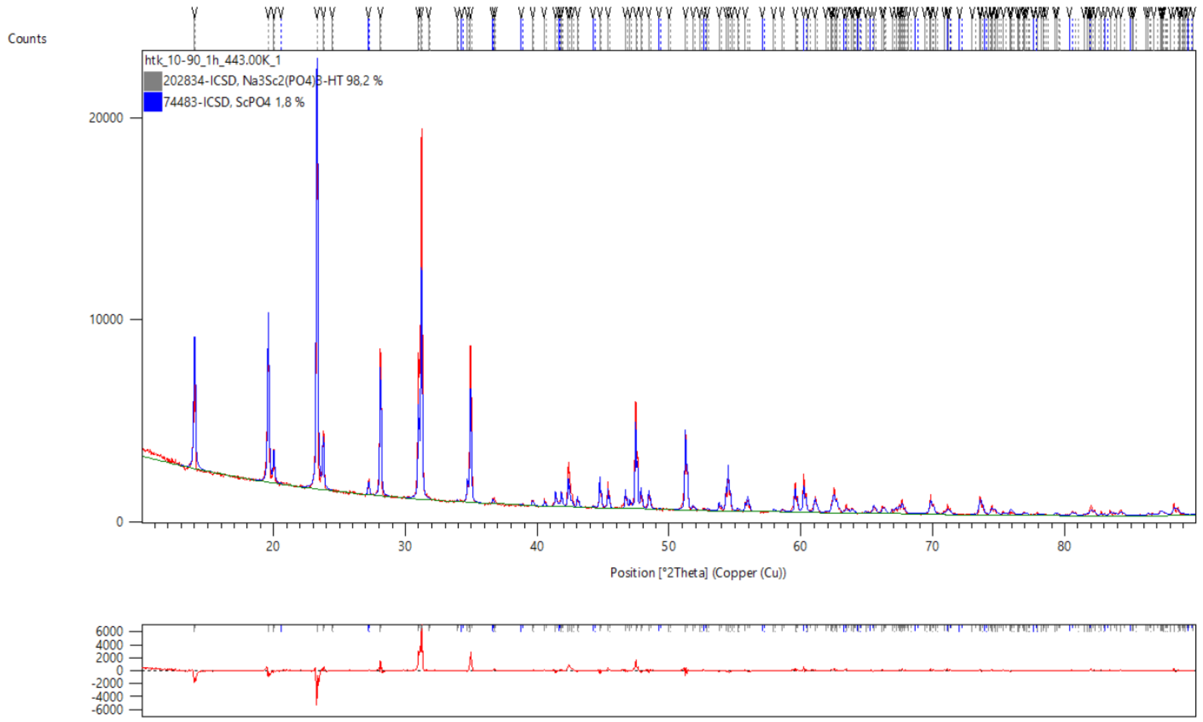

Figure S17. Rietveld refinement of XRD pattern of $Na_3Sc_2(PO_4)_3$:0.2%Eu^{3+} measured at 443K.

$Na_3Sc_2(PO_4)_3$:0.2%Eu^{3+} measured at 453K

Global Parameters

Number of used phases:	2
Number of variables:	14
Number of constraints:	1
Zero shift/ °2Theta:	0,000000

Specimen displacement/ mm :	-0,259(2)
Profile function:	Pseudo Voigt
Background:	Polynomial
R (expected)/ %:	2,83235
R (profile)/ %:	7,97738
R (weighted profile)/ %:	12,29427
GOF:	18,84136
d-statistic:	0,28418
U standard:	0,000000
V standard:	0,000000
W standard:	0,010000
U Left:	0,000000
V Left:	0,000000
W Left:	0,010000
U Right:	0,000000
V Right:	0,000000
W Right:	0,010000
Asymmetry Type:	No Asymmetry Function
Asymmetry 1:	0,000000
Asymmetry 2:	0,000000
Shape Type:	Shape Individual
Shape 1 Left:	0,600000
Shape 2 Left:	0,000000
Shape 3 Left:	0,000000
Shape 1 Right:	0,600000
Shape 2 Right:	0,000000
Shape 3 Right:	0,000000
K a1/a2 intensity ratio:	0,500000
K alpha/beta intensity ratio:	0,000000
Crystal Shape Factor K:	1,0000
Instrumental FWHM Curve Type:	Caglioti function
Instr. Gauss Curve Coefficient A:	0,0045(5)
Instr. Gauss Curve Coefficient B:	-0,0032(9)
Instr. Gauss Curve Coefficient C:	0,0046(3)
Instr. Lorentz Curve Coefficient A:	0,0062(7)
Instr. Lorentz Curve Coefficient B:	-0,004(1)
Instr. Lorentz Curve Coefficient C:	0,0064(5)

Relevant parameters of 202834-ICSD, Na3Sc2(PO4)3-HT

Structure and profile data:	
Formula sum:	$Sc_{12,00}P_{18,00}O_{72,00}Na_{7,16}$
Formula mass/ g/mol:	2413,6550
Density (calculated)/ g/cm^3	2,5763
F(000):	1176,8040
Weight fraction/ %:	98(1)
Space group (No.):	R -3 c (167)
Lattice parameters:	
a/ A:	8,9188(3)
b/ A:	8,9188(3)
c/ A:	22,579(1)
alpha/ °:	90
beta/ °:	90
gamma/ °:	120
V/ 10^6 pm^3	1555,46900
Overall displacement parameter:	0,000000
Extinction:	0,000000
Flat Plate Absorption Correction:	0,000000
Porosity:	0,000000
Roughness:	0,000000
Fitting mode:	Structure Fit
U Left:	0,19(2)
V Left:	-0,13(1)
W Left:	0,031(2)
Preferred orientation direction/ hkl:	0,00 0,00 1,00
Preferred orientation parameter:	1,000000
Asymmetry parameter 1:	0,000000
Asymmetry parameter 2:	0,000000
Peak shape:	
parameter 1 Left:	0,64(3)
parameter 2 Left:	0,000000
parameter 3 Left:	0,000000
R (Bragg)/ %:	14,10084

Occupancy, atomic fract. coordinates and Biso for 202834-ICSD, Na3Sc2(PO4)3-HT

Atom	Wyck.	s.o.f.	x	y	z	B/ 10^4 pm^2
Sc1	12c	1,000000	0,000000	0,000000	0,148700	0,000000
P1	18e	1,000000	0,295100	0,000000	0,250000	0,000000
O1	36f	1,000000	0,023500	0,209100	0,194600	0,000000
O2	36f	1,000000	0,192170	0,172830	0,088730	0,000000
Na1	18e	0,347000	0,637200	0,000000	0,250000	0,000000
Na2	6b	0,153000	0,000000	0,000000	0,000000	0,000000

Relevant parameters of 74483-ICSD, ScPO4

Structure and profile data:	
Formula sum:	$Sc_{4,00}P_{4,00}O_{16,00}$
Formula mass/ g/mol:	559,7092
Density (calculated)/ g/cm^3	3,7043
F(000):	272,0000
Weight fraction/ %:	2,0(2)
Space group (No.):	I 41/a m d (141)
Lattice parameters:	
a/ A:	6,579000
b/ A:	6,579000
c/ A:	5,796000
alpha/ °:	90
beta/ °:	90
gamma/ °:	90
V/ 10^6 pm^3	250,86960
Overall displacement parameter:	0,000000
Extinction:	0,000000
Flat Plate Absorption Correction:	0,000000
Porosity:	0,000000
Roughness:	0,000000
Fitting mode:	Structure Fit
U Left:	0,000000
V Left:	0,000000
W Left:	0,010000
Preferred orientation direction/ hkl:	0,00 0,00 1,00
Preferred orientation parameter:	1,000000
Asymmetry parameter 1:	0,000000
Asymmetry parameter 2:	0,000000
Peak shape:	
parameter 1 Left:	0,600000
parameter 2 Left:	0,000000
parameter 3 Left:	0,000000
R (Bragg)/ %:	3,86710

Occupancy, atomic fract. coordinates and Biso for 74483-ICSD, ScPO4

Atom	Wyck.	s.o.f.	x	y	z	B/ 10^4 pm^2
Sc1	4a	1,000000	0,000000	0,750000	0,125000	0,000000
P1	4b	1,000000	0,000000	0,250000	0,375000	0,000000
O1	16h	1,000000	0,000000	0,068900	0,208400	0,378992

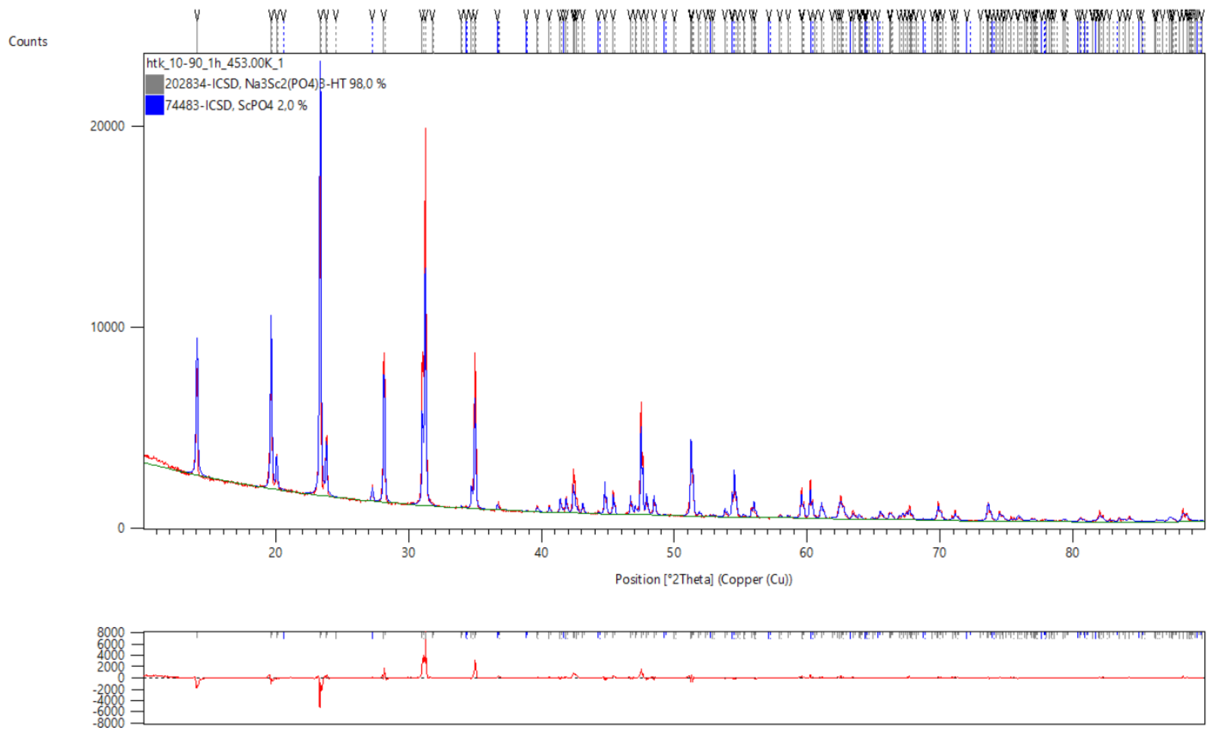

Figure S18. Rietveld refinement of XRD pattern of $Na_3Sc_2(PO_4)_3$:0.2%Eu^{3+} measured at 453K.

<u>$Na_3Sc_2(PO_4)_3$:0.2%Eu^{3+} measured at 463K</u>

<u>Global Parameters</u>

Number of used phases:	2
Number of variables:	14
Number of constraints:	1
Zero shift/ °2Theta:	0,000000
Specimen displacement/ mm :	-0,259(2)
Profile function:	Pseudo Voigt
Background:	Polynomial
R (expected)/ %:	2,83200
R (profile)/ %:	7,85961
R (weighted profile)/ %:	12,09280
GOF:	18,23339
d-statistic:	0,29663
U standard:	0,000000
V standard:	0,000000
W standard:	0,010000
U Left:	0,000000
V Left:	0,000000
W Left:	0,010000
U Right:	0,000000
V Right:	0,000000
W Right:	0,010000
Asymmetry Type:	No Asymmetry Function
Asymmetry 1:	0,000000
Asymmetry 2:	0,000000
Shape Type:	Shape Individual
Shape 1 Left:	0,600000
Shape 2 Left:	0,000000
Shape 3 Left:	0,000000
Shape 1 Right:	0,600000
Shape 2 Right:	0,000000
Shape 3 Right:	0,000000
K a1/a2 intensity ratio:	0,500000
K alpha/beta intensity ratio:	0,000000
Crystal Shape Factor K:	1,0000

Instrumental FWHM Curve Type:	Caglioti function
Instr. Gauss Curve Coefficient A:	0,0045(5)
Instr. Gauss Curve Coefficient B:	-0,0032(9)
Instr. Gauss Curve Coefficient C:	0,0046(3)
Instr. Lorentz Curve Coefficient A:	0,0062(7)
Instr. Lorentz Curve Coefficient B:	-0,004(1)
Instr. Lorentz Curve Coefficient C:	0,0064(5)

Relevant parameters of 202834-ICSD, Na3Sc2(PO4)3-HT

Structure and profile data:	
Formula sum:	$Sc_{12,00}P_{18,00}O_{72,00}Na_{7,16}$
Formula mass/ g/mol:	2413,6550
Density (calculated)/ g/cm^3	2,5750
F(000):	1176,8040
Weight fraction/ %:	98(1)
Space group (No.):	R -3 c (167)
Lattice parameters:	
a/ A:	8,9188(3)
b/ A:	8,9188(3)
c/ A:	22,591(1)
alpha/ °:	90
beta/ °:	90
gamma/ °:	120
V/ 10^6 pm^3	1556,26100
Overall displacement parameter:	0,000000
Extinction:	0,000000
Flat Plate Absorption Correction:	0,000000
Porosity:	0,000000
Roughness:	0,000000
Fitting mode:	Structure Fit
U Left:	0,19(1)
V Left:	-0,13(1)
W Left:	0,031(2)
Preferred orientation direction/ hkl:	0,00 0,00 1,00
Preferred orientation parameter:	1,000000
Asymmetry parameter 1:	0,000000
Asymmetry parameter 2:	0,000000
Peak shape:	
parameter 1 Left:	0,64(3)
parameter 2 Left:	0,000000
parameter 3 Left:	0,000000
R (Bragg)/ %:	13,72073

Occupancy, atomic fract. coordinates and Biso for 202834-ICSD, Na3Sc2(PO4)3-HT

Atom	Wyck.	s.o.f.	x	y	z	B/ 10^4 pm^2
Sc1	12c	1,000000	0,000000	0,000000	0,148700	0,000000
P1	18e	1,000000	0,295100	0,000000	0,250000	0,000000
O1	36f	1,000000	0,023500	0,209100	0,194600	0,000000
O2	36f	1,000000	0,192170	0,172830	0,088730	0,000000
Na1	18e	0,347000	0,637200	0,000000	0,250000	0,000000
Na2	6b	0,153000	0,000000	0,000000	0,000000	0,000000

Relevant parameters of 74483-ICSD, ScPO4

Structure and profile data:	
Formula sum:	$Sc_{4,00}P_{4,00}O_{16,00}$
Formula mass/ g/mol:	559,7092
Density (calculated)/ g/cm^3	3,7043
F(000):	272,0000
Weight fraction/ %:	1,9(2)
Space group (No.):	I 41/a m d (141)
Lattice parameters:	
a/ A:	6,579000
b/ A:	6,579000
c/ A:	5,796000
alpha/ °:	90
beta/ °:	90
gamma/ °:	90
V/ 10^6 pm^3	250,86960

Overall displacement parameter:	0,000000
Extinction:	0,000000
Flat Plate Absorption Correction:	0,000000
Porosity:	0,000000
Roughness:	0,000000
Fitting mode:	Structure Fit
U Left:	0,000000
V Left:	0,000000
W Left:	0,010000
Preferred orientation direction/ hkl:	0,00 0,00 1,00
Preferred orientation parameter:	1,000000
Asymmetry parameter 1:	0,000000
Asymmetry parameter 2:	0,000000
Peak shape:	
parameter 1 Left:	0,600000
parameter 2 Left:	0,000000
parameter 3 Left:	0,000000
R (Bragg)/ %:	3,78962

Occupancy, atomic fract. coordinates and Biso for 74483-ICSD, ScPO4

Atom	Wyck.	s.o.f.	x	y	z	B/ 10^4 pm^2
Sc1	4a	1,000000	0,000000	0,750000	0,125000	0,000000
P1	4b	1,000000	0,000000	0,250000	0,375000	0,000000
O1	16h	1,000000	0,000000	0,068900	0,208400	0,378992

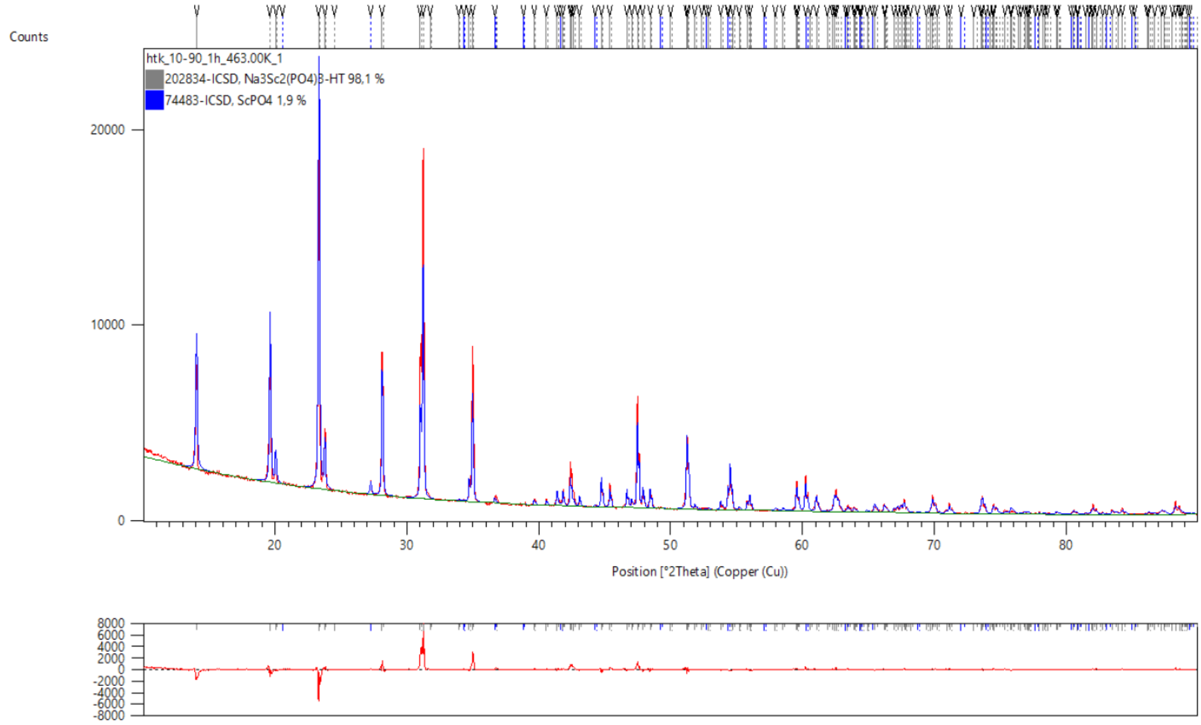

Figure S19. Rietveld refinement of XRD pattern of $Na_3Sc_2(PO_4)_3$:0.2%Eu^{3+} measured at 463K.

$Na_3Sc_2(PO_4)_3$:0.2%Eu^{3+} measured at 473K

Global Parameters

Number of used phases:	2
Number of variables:	14
Number of constraints:	1

Zero shift/ °2Theta:	0,000000
Specimen displacement/ mm :	-0,239(2)
Profile function:	Pseudo Voigt
Background:	Polynomial
R (expected)/ %:	2,84203
R (profile)/ %:	8,03280
R (weighted profile)/ %:	12,29875
GOF:	18,72680
d-statistic:	0,27875
U standard:	0,000000
V standard:	0,000000
W standard:	0,010000
U Left:	0,000000
V Left:	0,000000
W Left:	0,010000
U Right:	0,000000
V Right:	0,000000
W Right:	0,010000
Asymmetry Type:	No Asymmetry Function
Asymmetry 1:	0,000000
Asymmetry 2:	0,000000
Shape Type:	Shape Individual
Shape 1 Left:	0,600000
Shape 2 Left:	0,000000
Shape 3 Left:	0,000000
Shape 1 Right:	0,600000
Shape 2 Right:	0,000000
Shape 3 Right:	0,000000
K a1/a2 intensity ratio:	0,500000
K alpha/beta intensity ratio:	0,000000
Crystal Shape Factor K:	1,0000
Instrumental FWHM Curve Type:	Caglioti function
Instr. Gauss Curve Coefficient A:	0,0045(5)
Instr. Gauss Curve Coefficient B:	-0,0032(9)
Instr. Gauss Curve Coefficient C:	0,0046(3)
Instr. Lorentz Curve Coefficient A:	0,0062(7)
Instr. Lorentz Curve Coefficient B:	-0,004(1)
Instr. Lorentz Curve Coefficient C:	0,0064(5)

Relevant parameters of 202834-ICSD, Na3Sc2(PO4)3-HT

Structure and profile data:	
Formula sum:	$Sc_{12,00}P_{18,00}O_{72,00}Na_{7,16}$
Formula mass/ g/mol:	2413,6550
Density (calculated)/ g/cm^3	2,5732
F(000):	1176,8040
Weight fraction/ %:	98(1)
Space group (No.):	R -3 c (167)
Lattice parameters:	
a/ A:	8,9194(3)
b/ A:	8,9194(3)
c/ A:	22,604(1)
alpha/ °:	90
beta/ °:	90
gamma/ °:	120
V/ 10^6 pm^3	1557,34700
Overall displacement parameter:	0,000000
Extinction:	0,000000
Flat Plate Absorption Correction:	0,000000
Porosity:	0,000000
Roughness:	0,000000
Fitting mode:	Structure Fit
U Left:	0,18(1)
V Left:	-0,13(1)
W Left:	0,029(2)
Preferred orientation direction/ hkl:	0,00 0,00 1,00
Preferred orientation parameter:	1,000000
Asymmetry parameter 1:	0,000000
Asymmetry parameter 2:	0,000000
Peak shape:	
parameter 1 Left:	0,65(3)
parameter 2 Left:	0,000000
parameter 3 Left:	0,000000
R (Bragg)/ %:	14,14118

Occupancy, atomic fract. coordinates and Biso for 202834-ICSD, Na3Sc2(PO4)3-HT

Atom	Wyck.	s.o.f.	x	y	z	B/ 10^4 pm^2
Sc1	12c	1,000000	0,000000	0,000000	0,148700	0,000000
P1	18e	1,000000	0,295100	0,000000	0,250000	0,000000
O1	36f	1,000000	0,023500	0,209100	0,194600	0,000000
O2	36f	1,000000	0,192170	0,172830	0,088730	0,000000
Na1	18e	0,347000	0,637200	0,000000	0,250000	0,000000
Na2	6b	0,153000	0,000000	0,000000	0,000000	0,000000

Relevant parameters of 74483-ICSD, ScPO4

Structure and profile data:	
Formula sum:	$Sc_{4,00}P_{4,00}O_{16,00}$
Formula mass/ g/mol:	559,7092
Density (calculated)/ g/cm^3	3,7043
F(000):	272,0000
Weight fraction/ %:	1,9(2)
Space group (No.):	I 41/a m d (141)
Lattice parameters:	
a/ A:	6,579000
b/ A:	6,579000
c/ A:	5,796000
alpha/ °:	90
beta/ °:	90
gamma/ °:	90
V/ 10^6 pm^3	250,86960
Overall displacement parameter:	0,000000
Extinction:	0,000000
Flat Plate Absorption Correction:	0,000000
Porosity:	0,000000
Roughness:	0,000000
Fitting mode:	Structure Fit
U Left:	0,000000
V Left:	0,000000
W Left:	0,010000
Preferred orientation direction/ hkl:	0,00 0,00 1,00
Preferred orientation parameter:	1,000000
Asymmetry parameter 1:	0,000000
Asymmetry parameter 2:	0,000000
Peak shape:	
parameter 1 Left:	0,600000
parameter 2 Left:	0,000000
parameter 3 Left:	0,000000
R (Bragg)/ %:	4,59120

Occupancy, atomic fract. coordinates and Biso for 74483-ICSD, ScPO4

Atom	Wyck.	s.o.f.	x	y	z	B/ 10^4 pm^2
Sc1	4a	1,000000	0,000000	0,750000	0,125000	0,000000
P1	4b	1,000000	0,000000	0,250000	0,375000	0,000000
O1	16h	1,000000	0,000000	0,068900	0,208400	0,378992

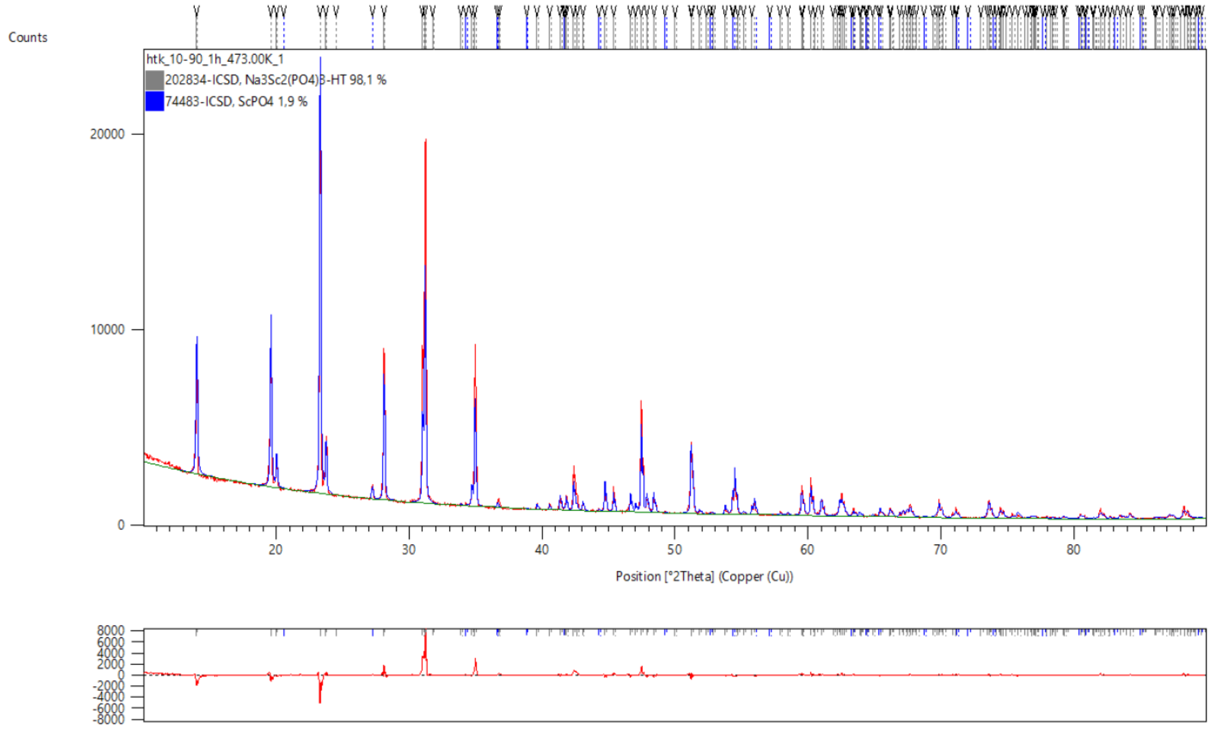

Figure S20. Rietveld refinement of XRD pattern of $Na_3Sc_2(PO_4)_3$:0.2%Eu^{3+} measured at 473K.

$Na_3Sc_2(PO_4)_3$:0.2%Eu^{3+} measured at 503K

Global Parameters

Number of used phases:	2
Number of variables:	14
Number of constraints:	1
Zero shift/ °2Theta:	0,000000
Specimen displacement/ mm :	-0,264(2)
Profile function:	Pseudo Voigt
Background:	Polynomial
R (expected)/ %:	2,84874
R (profile)/ %:	8,02656
R (weighted profile)/ %:	12,26684
GOF:	18,54220
d-statistic:	0,29379
U standard:	0,000000
V standard:	0,000000
W standard:	0,010000
U Left:	0,000000
V Left:	0,000000
W Left:	0,010000
U Right:	0,000000
V Right:	0,000000
W Right:	0,010000
Asymmetry Type:	No Asymmetry Function
Asymmetry 1:	0,000000
Asymmetry 2:	0,000000
Shape Type:	Shape Individual
Shape 1 Left:	0,600000
Shape 2 Left:	0,000000
Shape 3 Left:	0,000000
Shape 1 Right:	0,600000
Shape 2 Right:	0,000000
Shape 3 Right:	0,000000
K a1/a2 intensity ratio:	0,500000
K alpha/beta intensity ratio:	0,000000
Crystal Shape Factor K:	1,0000

Instrumental FWHM Curve Type:	Caglioti function
Instr. Gauss Curve Coefficient A:	0,0045(5)
Instr. Gauss Curve Coefficient B:	-0,0032(9)
Instr. Gauss Curve Coefficient C:	0,0046(3)
Instr. Lorentz Curve Coefficient A:	0,0062(7)
Instr. Lorentz Curve Coefficient B:	-0,004(1)
Instr. Lorentz Curve Coefficient C:	0,0064(5)

Relevant parameters of 202834-ICSD, Na3Sc2(PO4)3-HT

Structure and profile data:	
Formula sum:	$Sc_{12,00}P_{18,00}O_{72,00}Na_{7,16}$
Formula mass/ g/mol:	2413,6550
Density (calculated)/ g/cm^3	2,5699
F(000):	1176,8040
Weight fraction/ %:	98(1)
Space group (No.):	R -3 c (167)
Lattice parameters:	
a/ A:	8,9195(3)
b/ A:	8,9195(3)
c/ A:	22,633(1)
alpha/ °:	90
beta/ °:	90
gamma/ °:	120
V/ 10^6 pm^3	1559,36700
Overall displacement parameter:	0,000000
Extinction:	0,000000
Flat Plate Absorption Correction:	0,000000
Porosity:	0,000000
Roughness:	0,000000
Fitting mode:	Structure Fit
U Left:	0,17(1)
V Left:	-0,12(1)
W Left:	0,028(2)
Preferred orientation direction/ hkl:	0,00 0,00 1,00
Preferred orientation parameter:	1,000000
Asymmetry parameter 1:	0,000000
Asymmetry parameter 2:	0,000000
Peak shape:	
parameter 1 Left:	0,63(3)
parameter 2 Left:	0,000000
parameter 3 Left:	0,000000
R (Bragg)/ %:	14,12145

Occupancy, atomic fract. coordinates and Biso for 202834-ICSD, Na3Sc2(PO4)3-HT

Atom	Wyck.	s.o.f.	x	y	z	B/ 10^4 pm^2
Sc1	12c	1,000000	0,000000	0,000000	0,148700	0,000000
P1	18e	1,000000	0,295100	0,000000	0,250000	0,000000
O1	36f	1,000000	0,023500	0,209100	0,194600	0,000000
O2	36f	1,000000	0,192170	0,172830	0,088730	0,000000
Na1	18e	0,347000	0,637200	0,000000	0,250000	0,000000
Na2	6b	0,153000	0,000000	0,000000	0,000000	0,000000

Relevant parameters of 74483-ICSD, ScPO4

Structure and profile data:	
Formula sum:	$Sc_{4,00}P_{4,00}O_{16,00}$
Formula mass/ g/mol:	559,7092
Density (calculated)/ g/cm^3	3,7043
F(000):	272,0000
Weight fraction/ %:	1,9(2)
Space group (No.):	I 41/a m d (141)
Lattice parameters:	
a/ A:	6,579000
b/ A:	6,579000
c/ A:	5,796000
alpha/ °:	90
beta/ °:	90
gamma/ °:	90
V/ 10^6 pm^3	250,86960

Overall displacement parameter:	0,000000
Extinction:	0,000000
Flat Plate Absorption Correction:	0,000000
Porosity:	0,000000
Roughness:	0,000000
Fitting mode:	Structure Fit
U Left:	0,000000
V Left:	0,000000
W Left:	0,010000
Preferred orientation direction/ hkl:	0,00 0,00 1,00
Preferred orientation parameter:	1,000000
Asymmetry parameter 1:	0,000000
Asymmetry parameter 2:	0,000000
Peak shape:	
parameter 1 Left:	0,600000
parameter 2 Left:	0,000000
parameter 3 Left:	0,000000
R (Bragg)/ %:	4,57713

Occupancy, atomic fract. coordinates and Biso for 74483-ICSD, ScPO4

Atom	Wyck.	s.o.f.	x	y	z	B/ 10^4 pm^2
Sc1	4a	1,000000	0,000000	0,750000	0,125000	0,000000
P1	4b	1,000000	0,000000	0,250000	0,375000	0,000000
O1	16h	1,000000	0,000000	0,068900	0,208400	0,378992

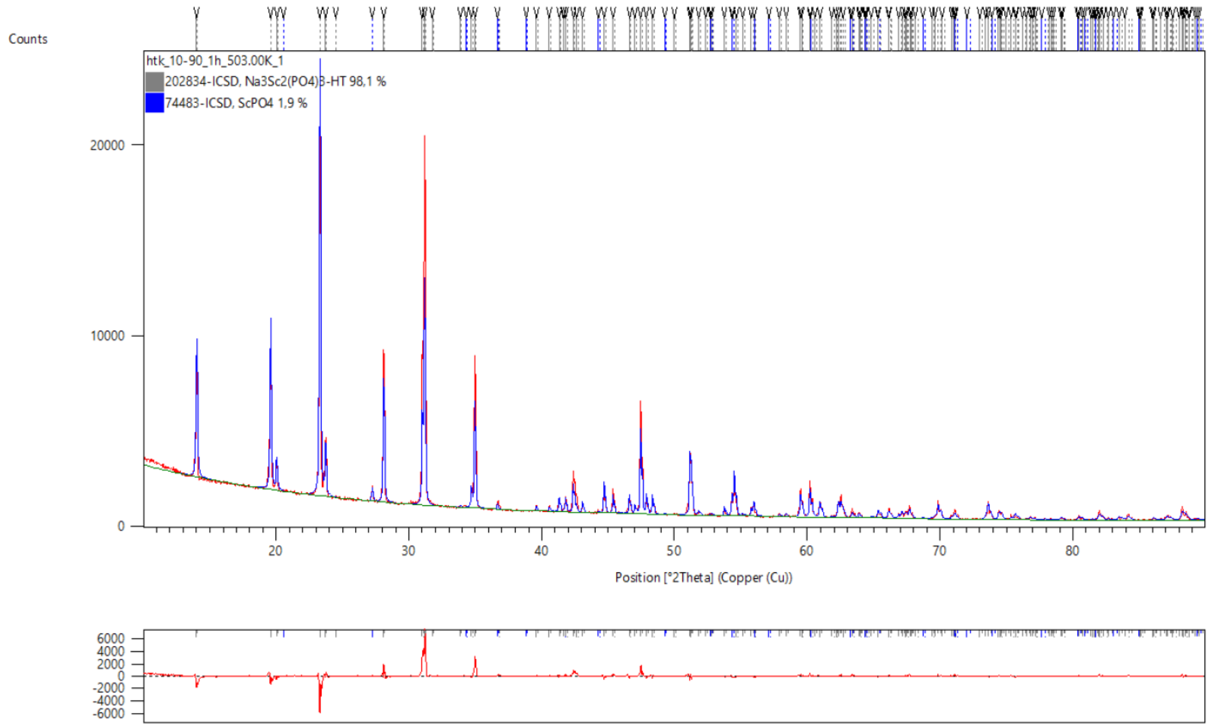

Figure S21. Rietveld refinement of XRD pattern of $Na_3Sc_2(PO_4)_3$:0.2%Eu^{3+} measured at 503K.

$Na_3Sc_2(PO_4)_3$:0.2%Eu^{3+} measured at 533K

Global Parameters

Number of used phases:	2
Number of variables:	14
Number of constraints:	1

Zero shift/ °2Theta:	0,000000
Specimen displacement/ mm :	-0,308(2)
Profile function:	Pseudo Voigt
Background:	Polynomial
R (expected)/ %:	2,84970
R (profile)/ %:	8,03144
R (weighted profile)/ %:	12,21415
GOF:	18,37082
d-statistic:	0,27730
U standard:	0,000000
V standard:	0,000000
W standard:	0,010000
U Left:	0,000000
V Left:	0,000000
W Left:	0,010000
U Right:	0,000000
V Right:	0,000000
W Right:	0,010000
Asymmetry Type:	No Asymmetry Function
Asymmetry 1:	0,000000
Asymmetry 2:	0,000000
Shape Type:	Shape Individual
Shape 1 Left:	0,600000
Shape 2 Left:	0,000000
Shape 3 Left:	0,000000
Shape 1 Right:	0,600000
Shape 2 Right:	0,000000
Shape 3 Right:	0,000000
K a1/a2 intensity ratio:	0,500000
K alpha/beta intensity ratio:	0,000000
Crystal Shape Factor K:	1,0000
Instrumental FWHM Curve Type:	Caglioti function
Instr. Gauss Curve Coefficient A:	0,0045(5)
Instr. Gauss Curve Coefficient B:	-0,0032(9)
Instr. Gauss Curve Coefficient C:	0,0046(3)
Instr. Lorentz Curve Coefficient A:	0,0062(7)
Instr. Lorentz Curve Coefficient B:	-0,004(1)
Instr. Lorentz Curve Coefficient C:	0,0064(5)

Relevant parameters of 202834-ICSD, Na3Sc2(PO4)3-HT

Structure and profile data:	
Formula sum:	$Sc_{12,00}P_{18,00}O_{72,00}Na_{7,16}$
Formula mass/ g/mol:	2413,6550
Density (calculated)/ g/cm^3	2,5673
F(000):	1176,8040
Weight fraction/ %:	98(1)
Space group (No.):	R -3 c (167)
Lattice parameters:	
a/ A:	8,9193(3)
b/ A:	8,9193(3)
c/ A:	22,656(1)
alpha/ °:	90
beta/ °:	90
gamma/ °:	120
V/ 10^6 pm^3	1560,92600
Overall displacement parameter:	0,000000
Extinction:	0,000000
Flat Plate Absorption Correction:	0,000000
Porosity:	0,000000
Roughness:	0,000000
Fitting mode:	Structure Fit
U Left:	0,16(1)
V Left:	-0,112(9)
W Left:	0,027(2)
Preferred orientation direction/ hkl:	0,00 0,00 1,00
Preferred orientation parameter:	1,000000
Asymmetry parameter 1:	0,000000
Asymmetry parameter 2:	0,000000
Peak shape:	
parameter 1 Left:	0,62(3)
parameter 2 Left:	0,000000
parameter 3 Left:	0,000000
R (Bragg)/ %:	14,33927

Occupancy, atomic fract. coordinates and Biso for 202834-ICSD, Na3Sc2(PO4)3-HT

Atom	Wyck.	s.o.f.	x	y	z	B/ 10^4 pm^2
Sc1	12c	1,000000	0,000000	0,000000	0,148700	0,000000
P1	18e	1,000000	0,295100	0,000000	0,250000	0,000000
O1	36f	1,000000	0,023500	0,209100	0,194600	0,000000
O2	36f	1,000000	0,192170	0,172830	0,088730	0,000000
Na1	18e	0,347000	0,637200	0,000000	0,250000	0,000000
Na2	6b	0,153000	0,000000	0,000000	0,000000	0,000000

Relevant parameters of 74483-ICSD, ScPO4

Structure and profile data:	
Formula sum:	$Sc_{4,00}P_{4,00}O_{16,00}$
Formula mass/ g/mol:	559,7092
Density (calculated)/ g/cm^3	3,7043
F(000):	272,0000
Weight fraction/ %:	1,9(2)
Space group (No.):	I 41/a m d (141)
Lattice parameters:	
a/ A:	6,579000
b/ A:	6,579000
c/ A:	5,796000
alpha/ °:	90
beta/ °:	90
gamma/ °:	90
V/ 10^6 pm^3	250,86960
Overall displacement parameter:	0,000000
Extinction:	0,000000
Flat Plate Absorption Correction:	0,000000
Porosity:	0,000000
Roughness:	0,000000
Fitting mode:	Structure Fit
U Left:	0,000000
V Left:	0,000000
W Left:	0,010000
Preferred orientation direction/ hkl:	0,00 0,00 1,00
Preferred orientation parameter:	1,000000
Asymmetry parameter 1:	0,000000
Asymmetry parameter 2:	0,000000
Peak shape:	
parameter 1 Left:	0,600000
parameter 2 Left:	0,000000
parameter 3 Left:	0,000000
R (Bragg)/ %:	5,61107

Occupancy, atomic fract. coordinates and Biso for 74483-ICSD, ScPO4

Atom	Wyck.	s.o.f.	x	y	z	B/ 10^4 pm^2
Sc1	4a	1,000000	0,000000	0,750000	0,125000	0,000000
P1	4b	1,000000	0,000000	0,250000	0,375000	0,000000
O1	16h	1,000000	0,000000	0,068900	0,208400	0,378992

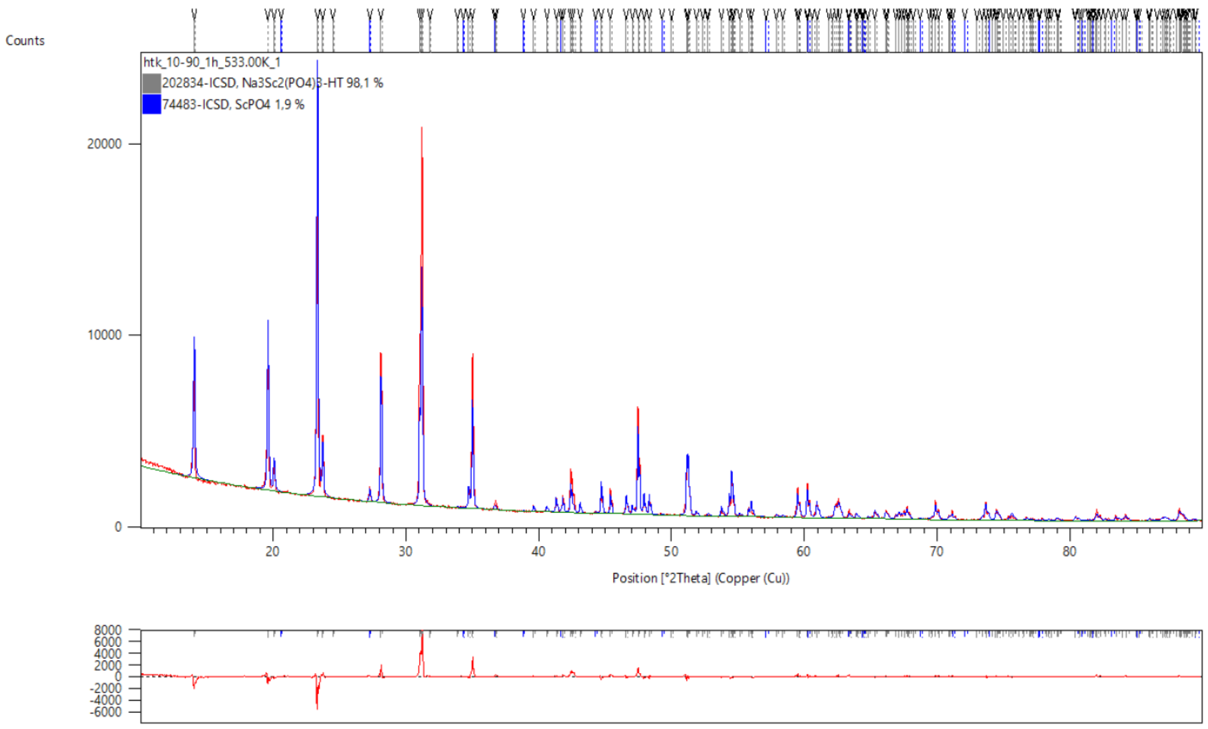

Figure S22. Rietveld refinement of XRD pattern of $Na_3Sc_2(PO_4)_3$:0.2%Eu^{3+} measured at 533K.

$Na_3Sc_2(PO_4)_3$:0.2%Eu^{3+} measured at 563K

Global Parameters

Number of used phases:	2
Number of variables:	14
Number of constraints:	1
Zero shift/ °2Theta:	0,000000
Specimen displacement/ mm :	-0,318(2)
Profile function:	Pseudo Voigt
Background:	Polynomial
R (expected)/ %:	2,84901
R (profile)/ %:	8,05492
R (weighted profile)/ %:	12,20750
GOF:	18,35964
d-statistic:	0,30398
U standard:	0,000000
V standard:	0,000000
W standard:	0,010000
U Left:	0,000000
V Left:	0,000000
W Left:	0,010000
U Right:	0,000000
V Right:	0,000000
W Right:	0,010000
Asymmetry Type:	No Asymmetry Function
Asymmetry 1:	0,000000
Asymmetry 2:	0,000000
Shape Type:	Shape Individual
Shape 1 Left:	0,600000
Shape 2 Left:	0,000000
Shape 3 Left:	0,000000
Shape 1 Right:	0,600000
Shape 2 Right:	0,000000
Shape 3 Right:	0,000000
K a1/a2 intensity ratio:	0,500000
K alpha/beta intensity ratio:	0,000000
Crystal Shape Factor K:	1,0000

Instrumental FWHM Curve Type:	Caglioti function
Instr. Gauss Curve Coefficient A:	0,0045(5)
Instr. Gauss Curve Coefficient B:	-0,0032(9)
Instr. Gauss Curve Coefficient C:	0,0046(3)
Instr. Lorentz Curve Coefficient A:	0,0062(7)
Instr. Lorentz Curve Coefficient B:	-0,004(1)
Instr. Lorentz Curve Coefficient C:	0,0064(5)

Relevant parameters of 202834-ICSD, Na3Sc2(PO4)3-HT

Structure and profile data:	
Formula sum:	$Sc_{12,00}P_{18,00}O_{72,00}Na_{7,16}$
Formula mass/ g/mol:	2413,6550
Density (calculated)/ g/cm^3	2,5642
F(000):	1176,8040
Weight fraction/ %:	98(1)
Space group (No.):	R -3 c (167)
Lattice parameters:	
a/ A:	8,9201(3)
b/ A:	8,9201(3)
c/ A:	22,680(1)
alpha/ °:	90
beta/ °:	90
gamma/ °:	120
V/ 10^6 pm^3	1562,80900
Overall displacement parameter:	0,000000
Extinction:	0,000000
Flat Plate Absorption Correction:	0,000000
Porosity:	0,000000
Roughness:	0,000000
Fitting mode:	Structure Fit
U Left:	0,15(1)
V Left:	-0,107(9)
W Left:	0,026(1)
Preferred orientation direction/ hkl:	0,00 0,00 1,00
Preferred orientation parameter:	1,000000
Asymmetry parameter 1:	0,000000
Asymmetry parameter 2:	0,000000
Peak shape:	
parameter 1 Left:	0,62(3)
parameter 2 Left:	0,000000
parameter 3 Left:	0,000000
R (Bragg)/ %:	14,10284

Occupancy, atomic fract. coordinates and Biso for 202834-ICSD, Na3Sc2(PO4)3-HT

Atom	Wyck.	s.o.f.	x	y	z	B/ 10^4 pm^2	
Sc1	12c	1,000000	0,000000	0,000000	0,148700	0,000000	
P1	18e	1,000000	0,295100	0,000000	0,250000	0,000000	
O1	36f	1,000000	0,023500	0,209100	0,194600	0,000000	
O2	36f	1,000000	0,192170	0,172830	0,088730	0,000000	
Na1	18e	0,347000	0,637200	0,000000	0,250000	0,000000	
Na2	6b	0,153000	0,000000	0,000000	0,000000	0,000000	

Relevant parameters of 74483-ICSD, ScPO4

Structure and profile data:	
Formula sum:	$Sc_{4,00}P_{4,00}O_{16,00}$
Formula mass/ g/mol:	559,7092
Density (calculated)/ g/cm^3	3,7043
F(000):	272,0000
Weight fraction/ %:	1,9(2)
Space group (No.):	I 41/a m d (141)
Lattice parameters:	
a/ A:	6,579000
b/ A:	6,579000
c/ A:	5,796000
alpha/ °:	90
beta/ °:	90
gamma/ °:	90
V/ 10^6 pm^3	250,86960

Overall displacement parameter:	0,000000
Extinction:	0,000000
Flat Plate Absorption Correction:	0,000000
Porosity:	0,000000
Roughness:	0,000000
Fitting mode:	Structure Fit
U Left:	0,000000
V Left:	0,000000
W Left:	0,010000
Preferred orientation direction/ hkl:	0,00 0,00 1,00
Preferred orientation parameter:	1,000000
Asymmetry parameter 1:	0,000000
Asymmetry parameter 2:	0,000000
Peak shape:	
parameter 1 Left:	0,600000
parameter 2 Left:	0,000000
parameter 3 Left:	0,000000
R (Bragg)/ %:	6,25379

Occupancy, atomic fract. coordinates and Biso for 74483-ICSD, ScPO4

Atom	Wyck.	s.o.f.	x	y	z	B/ 10^4 pm^2
Sc1	4a	1,000000	0,000000	0,750000	0,125000	0,000000
P1	4b	1,000000	0,000000	0,250000	0,375000	0,000000
O1	16h	1,000000	0,000000	0,068900	0,208400	0,378992

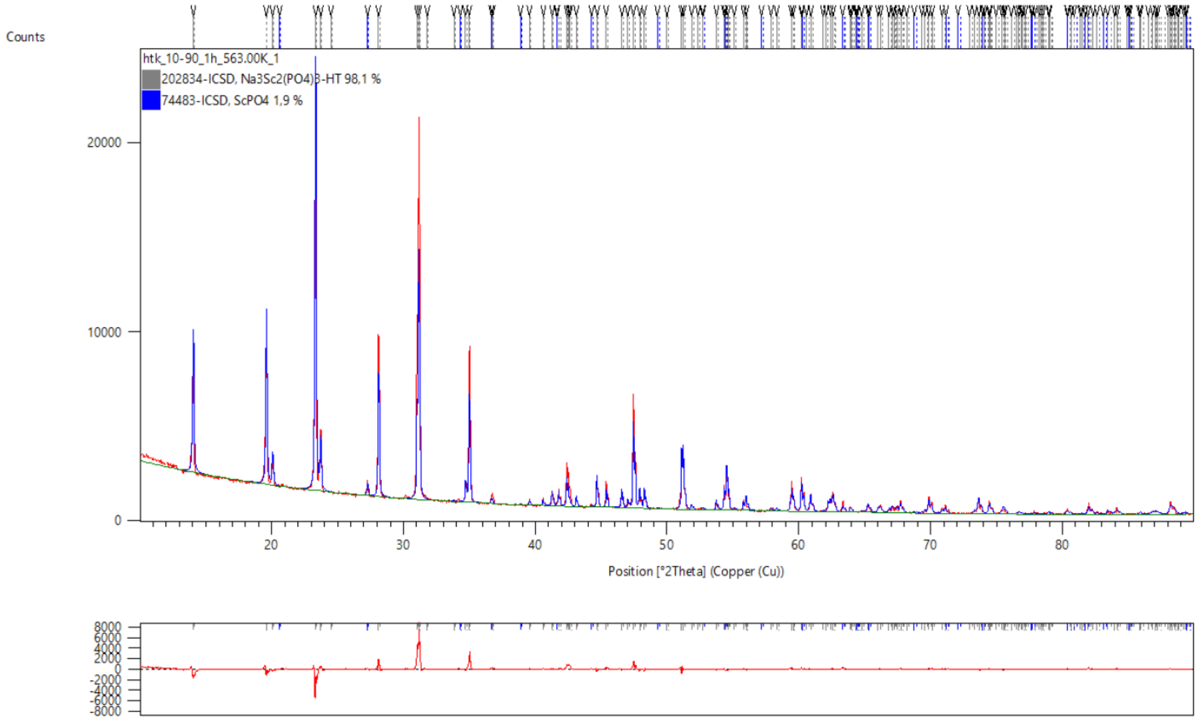

Figure S23. Rietveld refinement of XRD pattern of $Na_3Sc_2(PO_4)_3$:0.2%Eu^{3+} measured at 563K.

$Na_3Sc_2(PO_4)_3$:0.2%Eu^{3+} measured at 593K

Global Parameters

Number of used phases:	2
Number of variables:	14
Number of constraints:	1

Zero shift/ °2Theta:	0,000000
Specimen displacement/ mm :	-0,399(2)
Profile function:	Pseudo Voigt
Background:	Polynomial
R (expected)/ %:	2,85967
R (profile)/ %:	8,16002
R (weighted profile)/ %:	12,21345
GOF:	18,24088
d-statistic:	0,28490
U standard:	0,000000
V standard:	0,000000
W standard:	0,010000
U Left:	0,000000
V Left:	0,000000
W Left:	0,010000
U Right:	0,000000
V Right:	0,000000
W Right:	0,010000
Asymmetry Type:	No Asymmetry Function
Asymmetry 1:	0,000000
Asymmetry 2:	0,000000
Shape Type:	Shape Individual
Shape 1 Left:	0,600000
Shape 2 Left:	0,000000
Shape 3 Left:	0,000000
Shape 1 Right:	0,600000
Shape 2 Right:	0,000000
Shape 3 Right:	0,000000
K a1/a2 intensity ratio:	0,500000
K alpha/beta intensity ratio:	0,000000
Crystal Shape Factor K:	1,0000
Instrumental FWHM Curve Type:	Caglioti function
Instr. Gauss Curve Coefficient A:	0,0045(5)
Instr. Gauss Curve Coefficient B:	-0,0032(9)
Instr. Gauss Curve Coefficient C:	0,0046(3)
Instr. Lorentz Curve Coefficient A:	0,0062(7)
Instr. Lorentz Curve Coefficient B:	-0,004(1)
Instr. Lorentz Curve Coefficient C:	0,0064(5)

Relevant parameters of 202834-ICSD, Na3Sc2(PO4)3-HT

Structure and profile data:	
Formula sum:	$Sc_{12,00}P_{18,00}O_{72,00}Na_{7,16}$
Formula mass/ g/mol:	2413,6550
Density (calculated)/ g/cm^3	2,5625
F(000):	1176,8040
Weight fraction/ %:	98(1)
Space group (No.):	R -3 c (167)
Lattice parameters:	
a/ A:	8,9197(3)
b/ A:	8,9197(3)
c/ A:	22,697(1)
alpha/ °:	90
beta/ °:	90
gamma/ °:	120
V/ 10^6 pm^3	1563,83200
Overall displacement parameter:	0,000000
Extinction:	0,000000
Flat Plate Absorption Correction:	0,000000
Porosity:	0,000000
Roughness:	0,000000
Fitting mode:	Structure Fit
U Left:	0,15(1)
V Left:	-0,104(8)
W Left:	0,026(1)
Preferred orientation direction/ hkl:	0,00 0,00 1,00
Preferred orientation parameter:	1,000000
Asymmetry parameter 1:	0,000000
Asymmetry parameter 2:	0,000000
Peak shape:	
parameter 1 Left:	0,61(3)
parameter 2 Left:	0,000000
parameter 3 Left:	0,000000
R (Bragg)/ %:	14,35137

Occupancy, atomic fract. coordinates and Biso for 202834-ICSD, Na3Sc2(PO4)3-HT

Atom	Wyck.	s.o.f.	x	y	z	B/ 10^4 pm^2
Sc1	12c	1,000000	0,000000	0,000000	0,148700	0,000000
P1	18e	1,000000	0,295100	0,000000	0,250000	0,000000
O1	36f	1,000000	0,023500	0,209100	0,194600	0,000000
O2	36f	1,000000	0,192170	0,172830	0,088730	0,000000
Na1	18e	0,347000	0,637200	0,000000	0,250000	0,000000
Na2	6b	0,153000	0,000000	0,000000	0,000000	0,000000

Relevant parameters of 74483-ICSD, ScPO4

Structure and profile data:	
Formula sum:	$Sc_{4,00}P_{4,00}O_{16,00}$
Formula mass/ g/mol:	559,7092
Density (calculated)/ g/cm^3	3,7043
F(000):	272,0000
Weight fraction/ %:	1,9(2)
Space group (No.):	I 41/a m d (141)
Lattice parameters:	
a/ A:	6,579000
b/ A:	6,579000
c/ A:	5,796000
alpha/ °:	90
beta/ °:	90
gamma/ °:	90
V/ 10^6 pm^3	250,86960
Overall displacement parameter:	0,000000
Extinction:	0,000000
Flat Plate Absorption Correction:	0,000000
Porosity:	0,000000
Roughness:	0,000000
Fitting mode:	Structure Fit
U Left:	0,000000
V Left:	0,000000
W Left:	0,010000
Preferred orientation direction/ hkl:	0,00 0,00 1,00
Preferred orientation parameter:	1,000000
Asymmetry parameter 1:	0,000000
Asymmetry parameter 2:	0,000000
Peak shape:	
parameter 1 Left:	0,600000
parameter 2 Left:	0,000000
parameter 3 Left:	0,000000
R (Bragg)/ %:	6,68891

Occupancy, atomic fract. coordinates and Biso for 74483-ICSD, ScPO4

Atom	Wyck.	s.o.f.	x	y	z	B/ 10^4 pm^2
Sc1	4a	1,000000	0,000000	0,750000	0,125000	0,000000
P1	4b	1,000000	0,000000	0,250000	0,375000	0,000000
O1	16h	1,000000	0,000000	0,068900	0,208400	0,378992

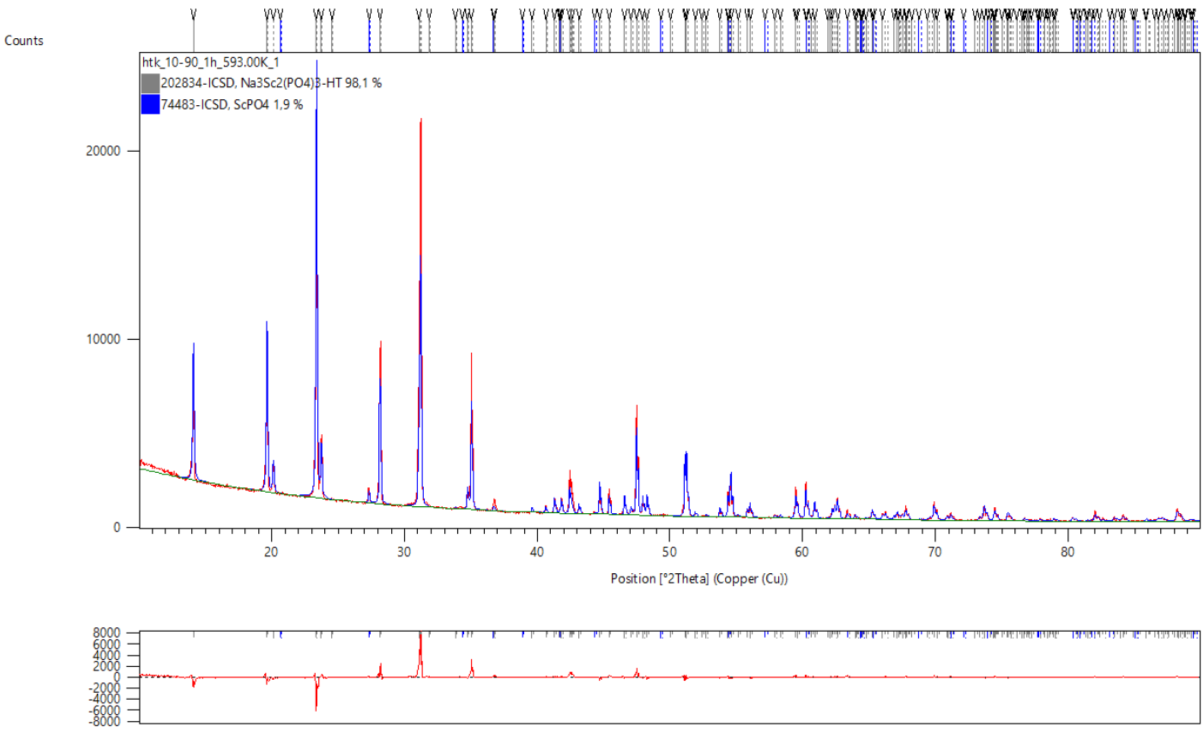

Figure S24. Rietveld refinement of XRD pattern of $Na_3Sc_2(PO_4)_3$:0.2%Eu^{3+} measured at 593K.

$Na_3Sc_2(PO_4)_3$:0.2%Eu^{3+} measured at 623K

Global Parameters

Number of used phases:	2
Number of variables:	13
Number of constraints:	1
Zero shift/ °2Theta:	0,000000
Specimen displacement/ mm :	-0,401549
Profile function:	Pseudo Voigt
Background:	Polynomial
R (expected)/ %:	2,86087
R (profile)/ %:	8,09988
R (weighted profile)/ %:	12,11122
GOF:	17,92177
d-statistic:	0,31299
U standard:	0,000000
V standard:	0,000000
W standard:	0,010000
U Left:	0,000000
V Left:	0,000000
W Left:	0,010000
U Right:	0,000000
V Right:	0,000000
W Right:	0,010000
Asymmetry Type:	No Asymmetry Function
Asymmetry 1:	0,000000
Asymmetry 2:	0,000000
Shape Type:	Shape Individual
Shape 1 Left:	0,600000
Shape 2 Left:	0,000000
Shape 3 Left:	0,000000
Shape 1 Right:	0,600000
Shape 2 Right:	0,000000
Shape 3 Right:	0,000000
K a1/a2 intensity ratio:	0,500000
K alpha/beta intensity ratio:	0,000000
Crystal Shape Factor K:	1,0000

Instrumental FWHM Curve Type:	Caglioti function
Instr. Gauss Curve Coefficient A:	0,0045(5)
Instr. Gauss Curve Coefficient B:	-0,0032(9)
Instr. Gauss Curve Coefficient C:	0,0046(3)
Instr. Lorentz Curve Coefficient A:	0,0062(7)
Instr. Lorentz Curve Coefficient B:	-0,004(1)
Instr. Lorentz Curve Coefficient C:	0,0064(5)

Relevant parameters of 202834-ICSD, Na3Sc2(PO4)3-HT

Structure and profile data:	
Formula sum:	$Sc_{12,00}P_{18,00}O_{72,00}Na_{7,16}$
Formula mass/ g/mol:	2413,6550
Density (calculated)/ g/cm^3	2,5604
F(000):	1176,8040
Weight fraction/ %:	98(1)
Space group (No.):	R -3 c (167)
Lattice parameters:	
a/ A:	8,9202(2)
b/ A:	8,9202(2)
c/ A:	22,7128(8)
alpha/ °:	90
beta/ °:	90
gamma/ °:	120
V/ 10^6 pm^3	1565,12400
Overall displacement parameter:	0,000000
Extinction:	0,000000
Flat Plate Absorption Correction:	0,000000
Porosity:	0,000000
Roughness:	0,000000
Fitting mode:	Structure Fit
U Left:	0,14(1)
V Left:	-0,104(8)
W Left:	0,026(1)
Preferred orientation direction/ hkl:	0,00 0,00 1,00
Preferred orientation parameter:	1,000000
Asymmetry parameter 1:	0,000000
Asymmetry parameter 2:	0,000000
Peak shape:	
parameter 1 Left:	0,61(3)
parameter 2 Left:	0,000000
parameter 3 Left:	0,000000
R (Bragg)/ %:	14,07819

Occupancy, atomic fract. coordinates and Biso for 202834-ICSD, Na3Sc2(PO4)3-HT

Atom	Wyck.	s.o.f.	x	y	z	B/ 10^4 pm^2
Sc1	12c	1,000000	0,000000	0,000000	0,148700	0,000000
P1	18e	1,000000	0,295100	0,000000	0,250000	0,000000
O1	36f	1,000000	0,023500	0,209100	0,194600	0,000000
O2	36f	1,000000	0,192170	0,172830	0,088730	0,000000
Na1	18e	0,347000	0,637200	0,000000	0,250000	0,000000
Na2	6b	0,153000	0,000000	0,000000	0,000000	0,000000

Relevant parameters of 74483-ICSD, ScPO4

Structure and profile data:	
Formula sum:	$Sc_{4,00}P_{4,00}O_{16,00}$
Formula mass/ g/mol:	559,7092
Density (calculated)/ g/cm^3	3,7043
F(000):	272,0000
Weight fraction/ %:	1,9(2)
Space group (No.):	I 41/a m d (141)
Lattice parameters:	
a/ A:	6,579000
b/ A:	6,579000
c/ A:	5,796000
alpha/ °:	90
beta/ °:	90
gamma/ °:	90
V/ 10^6 pm^3	250,86960

Overall displacement parameter:	0,000000
Extinction:	0,000000
Flat Plate Absorption Correction:	0,000000
Porosity:	0,000000
Roughness:	0,000000
Fitting mode:	Structure Fit
U Left:	0,000000
V Left:	0,000000
W Left:	0,010000
Preferred orientation direction/ hkl:	0,00 0,00 1,00
Preferred orientation parameter:	1,000000
Asymmetry parameter 1:	0,000000
Asymmetry parameter 2:	0,000000
Peak shape:	
parameter 1 Left:	0,600000
parameter 2 Left:	0,000000
parameter 3 Left:	0,000000
R (Bragg)/ %:	6,42435

Occupancy, atomic fract. coordinates and Biso for 74483-ICSD, ScPO4

Atom	Wyck.	s.o.f.	x	y	z	B/ 10^4 pm^2
Sc1	4a	1,000000	0,000000	0,750000	0,125000	0,000000
P1	4b	1,000000	0,000000	0,250000	0,375000	0,000000
O1	16h	1,000000	0,000000	0,068900	0,208400	0,378992

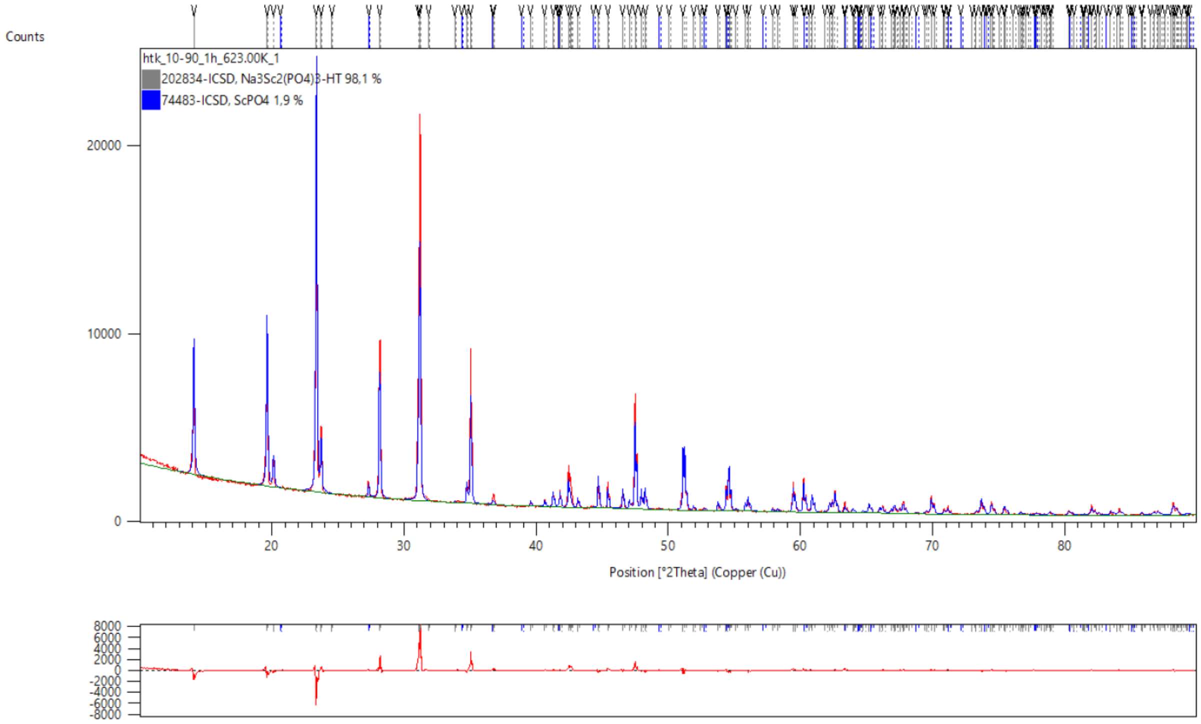

Figure S25. Rietveld refinement of XRD pattern of $Na_3Sc_2(PO_4)_3$:0.2%Eu^{3+} measured at 623K.

$Na_3Sc_2(PO_4)_3$:0.2%Eu^{3+} measured at 653K

Global Parameters

Number of used phases:	2
Number of variables:	14
Number of constraints:	1

Zero shift/ °2Theta:	0,000000
Specimen displacement/ mm :	-0,464(2)
Profile function:	Pseudo Voigt
Background:	Polynomial
R (expected)/ %:	2,86499
R (profile)/ %:	8,29462
R (weighted profile)/ %:	12,13894
GOF:	17,95214
d-statistic:	0,31579
U standard:	0,000000
V standard:	0,000000
W standard:	0,010000
U Left:	0,000000
V Left:	0,000000
W Left:	0,010000
U Right:	0,000000
V Right:	0,000000
W Right:	0,010000
Asymmetry Type:	No Asymmetry Function
Asymmetry 1:	0,000000
Asymmetry 2:	0,000000
Shape Type:	Shape Individual
Shape 1 Left:	0,600000
Shape 2 Left:	0,000000
Shape 3 Left:	0,000000
Shape 1 Right:	0,600000
Shape 2 Right:	0,000000
Shape 3 Right:	0,000000
K a1/a2 intensity ratio:	0,500000
K alpha/beta intensity ratio:	0,000000
Crystal Shape Factor K:	1,0000
Instrumental FWHM Curve Type:	Caglioti function
Instr. Gauss Curve Coefficient A:	0,0045(5)
Instr. Gauss Curve Coefficient B:	-0,0032(9)
Instr. Gauss Curve Coefficient C:	0,0046(3)
Instr. Lorentz Curve Coefficient A:	0,0062(7)
Instr. Lorentz Curve Coefficient B:	-0,004(1)
Instr. Lorentz Curve Coefficient C:	0,0064(5)

Relevant parameters of 202834-ICSD, Na3Sc2(PO4)3-HT

Structure and profile data:	
Formula sum:	$Sc_{12,00}P_{18,00}O_{72,00}Na_{7,16}$
Formula mass/ g/mol:	2413,6550
Density (calculated)/ g/cm^3	2,5569
F(000):	1176,8040
Weight fraction/ %:	98(1)
Space group (No.):	R -3 c (167)
Lattice parameters:	
a/ A:	8,9210(3)
b/ A:	8,9210(3)
c/ A:	22,7402(9)
alpha/ °:	90
beta/ °:	90
gamma/ °:	120
V/ 10^6 pm^3	1567,28900
Overall displacement parameter:	0,000000
Extinction:	0,000000
Flat Plate Absorption Correction:	0,000000
Porosity:	0,000000
Roughness:	0,000000
Fitting mode:	Structure Fit
U Left:	0,14(1)
V Left:	-0,104(8)
W Left:	0,026(1)
Preferred orientation direction/ hkl:	0,00 0,00 1,00
Preferred orientation parameter:	1,000000
Asymmetry parameter 1:	0,000000
Asymmetry parameter 2:	0,000000
Peak shape:	
parameter 1 Left:	0,60(3)
parameter 2 Left:	0,000000
parameter 3 Left:	0,000000
R (Bragg)/ %:	14,17356

Occupancy, atomic fract. coordinates and Biso for 202834-ICSD, Na3Sc2(PO4)3-HT

Atom	Wyck.	s.o.f.	x	y	z	B/ 10^4 pm^2
Sc1	12c	1,000000	0,000000	0,000000	0,148700	0,000000
P1	18e	1,000000	0,295100	0,000000	0,250000	0,000000
O1	36f	1,000000	0,023500	0,209100	0,194600	0,000000
O2	36f	1,000000	0,192170	0,172830	0,088730	0,000000
Na1	18e	0,347000	0,637200	0,000000	0,250000	0,000000
Na2	6b	0,153000	0,000000	0,000000	0,000000	0,000000

Relevant parameters of 74483-ICSD, ScPO4

Structure and profile data:	
Formula sum:	$Sc_{4,00}P_{4,00}O_{16,00}$
Formula mass/ g/mol:	559,7092
Density (calculated)/ g/cm^3	3,7043
F(000):	272,0000
Weight fraction/ %:	2,0(2)
Space group (No.):	I 41/a m d (141)
Lattice parameters:	
a/ A:	6,579000
b/ A:	6,579000
c/ A:	5,796000
alpha/ °:	90
beta/ °:	90
gamma/ °:	90
V/ 10^6 pm^3	250,86960
Overall displacement parameter:	0,000000
Extinction:	0,000000
Flat Plate Absorption Correction:	0,000000
Porosity:	0,000000
Roughness:	0,000000
Fitting mode:	Structure Fit
U Left:	0,000000
V Left:	0,000000
W Left:	0,010000
Preferred orientation direction/ hkl:	0,00 0,00 1,00
Preferred orientation parameter:	1,000000
Asymmetry parameter 1:	0,000000
Asymmetry parameter 2:	0,000000
Peak shape:	
parameter 1 Left:	0,600000
parameter 2 Left:	0,000000
parameter 3 Left:	0,000000
R (Bragg)/ %:	6,87881

Occupancy, atomic fract. coordinates and Biso for 74483-ICSD, ScPO4

Atom	Wyck.	s.o.f.	x	y	z	B/ 10^4 pm^2
Sc1	4a	1,000000	0,000000	0,750000	0,125000	0,000000
P1	4b	1,000000	0,000000	0,250000	0,375000	0,000000
O1	16h	1,000000	0,000000	0,068900	0,208400	0,378992

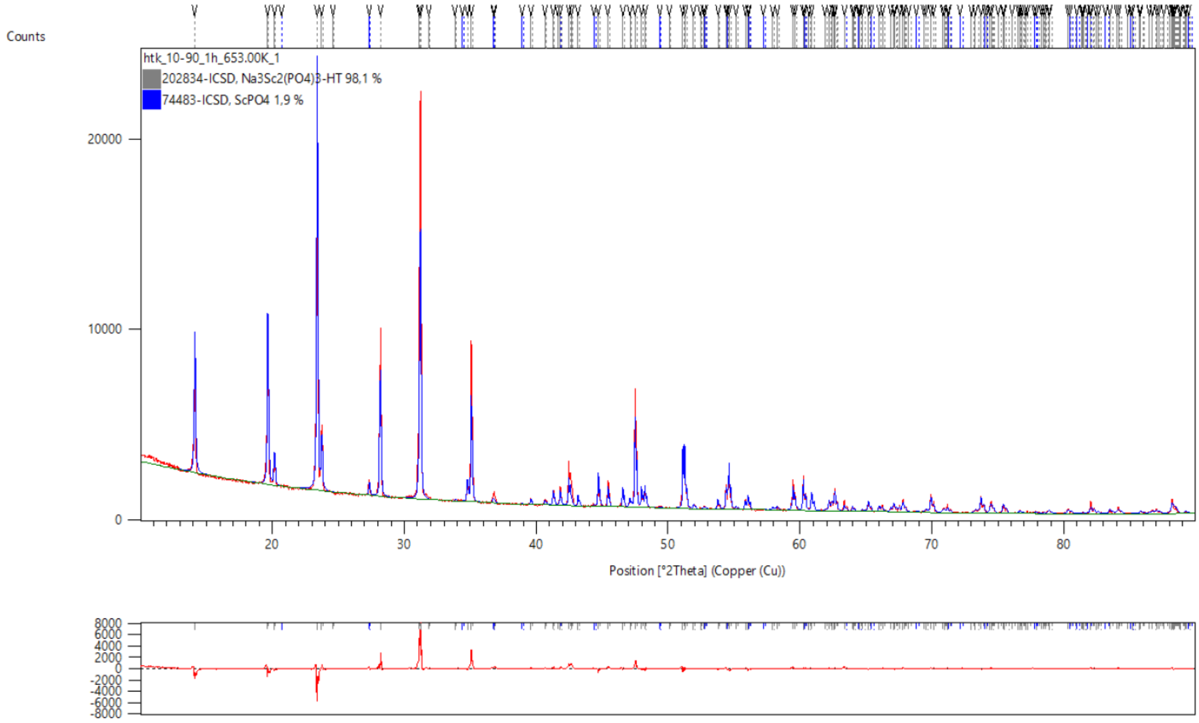

Figure S26. Rietveld refinement of XRD pattern of $Na_3Sc_2(PO_4)_3$:0.2%Eu^{3+} measured at 653K.

$Na_3Sc_2(PO_4)_3$:0.2%Eu^{3+} measured at 683K

Global Parameters

Number of used phases:	2
Number of variables:	14
Number of constraints:	1
Zero shift/ °2Theta:	0,000000
Specimen displacement/ mm :	-0,464(2)
Profile function:	Pseudo Voigt
Background:	Polynomial
R (expected)/ %:	2,86499
R (profile)/ %:	8,29462
R (weighted profile)/ %:	12,13894
GOF:	17,95214
d-statistic:	0,31579
U standard:	0,000000
V standard:	0,000000
W standard:	0,010000
U Left:	0,000000
V Left:	0,000000
W Left:	0,010000
U Right:	0,000000
V Right:	0,000000
W Right:	0,010000
Asymmetry Type:	No Asymmetry Function
Asymmetry 1:	0,000000
Asymmetry 2:	0,000000
Shape Type:	Shape Individual
Shape 1 Left:	0,600000
Shape 2 Left:	0,000000
Shape 3 Left:	0,000000
Shape 1 Right:	0,600000
Shape 2 Right:	0,000000
Shape 3 Right:	0,000000
K a1/a2 intensity ratio:	0,500000
K alpha/beta intensity ratio:	0,000000
Crystal Shape Factor K:	1,0000

Instrumental FWHM Curve Type:	Caglioti function
Instr. Gauss Curve Coefficient A:	0,0045(5)
Instr. Gauss Curve Coefficient B:	-0,0032(9)
Instr. Gauss Curve Coefficient C:	0,0046(3)
Instr. Lorentz Curve Coefficient A:	0,0062(7)
Instr. Lorentz Curve Coefficient B:	-0,004(1)
Instr. Lorentz Curve Coefficient C:	0,0064(5)

Relevant parameters of 202834-ICSD, Na3Sc2(PO4)3-HT

Structure and profile data:	
Formula sum:	$Sc_{12,00}P_{18,00}O_{72,00}Na_{7,16}$
Formula mass/ g/mol:	2413,6550
Density (calculated)/ g/cm^3	2,5569
F(000):	1176,8040
Weight fraction/ %:	98(1)
Space group (No.):	R -3 c (167)
Lattice parameters:	
a/ A:	8,9210(3)
b/ A:	8,9210(3)
c/ A:	22,7402(9)
alpha/ °:	90
beta/ °:	90
gamma/ °:	120
V/ 10^6 pm^3	1567,28900
Overall displacement parameter:	0,000000
Extinction:	0,000000
Flat Plate Absorption Correction:	0,000000
Porosity:	0,000000
Roughness:	0,000000
Fitting mode:	Structure Fit
U Left:	0,14(1)
V Left:	-0,104(8)
W Left:	0,026(1)
Preferred orientation direction/ hkl:	0,00 0,00 1,00
Preferred orientation parameter:	1,000000
Asymmetry parameter 1:	0,000000
Asymmetry parameter 2:	0,000000
Peak shape:	
parameter 1 Left:	0,60(3)
parameter 2 Left:	0,000000
parameter 3 Left:	0,000000
R (Bragg)/ %:	14,17356

Occupancy, atomic fract. coordinates and Biso for 202834-ICSD, Na3Sc2(PO4)3-HT

Atom	Wyck.	s.o.f.	x	y	z	B/ 10^4 pm^2
Sc1	12c	1,000000	0,000000	0,000000	0,148700	0,000000
P1	18e	1,000000	0,295100	0,000000	0,250000	0,000000
O1	36f	1,000000	0,023500	0,209100	0,194600	0,000000
O2	36f	1,000000	0,192170	0,172830	0,088730	0,000000
Na1	18e	0,347000	0,637200	0,000000	0,250000	0,000000
Na2	6b	0,153000	0,000000	0,000000	0,000000	0,000000

Relevant parameters of 74483-ICSD, ScPO4

Structure and profile data:	
Formula sum:	$Sc_{4,00}P_{4,00}O_{16,00}$
Formula mass/ g/mol:	559,7092
Density (calculated)/ g/cm^3	3,7043
F(000):	272,0000
Weight fraction/ %:	2,0(2)
Space group (No.):	I 41/a m d (141)
Lattice parameters:	
a/ A:	6,579000
b/ A:	6,579000
c/ A:	5,796000
alpha/ °:	90
beta/ °:	90
gamma/ °:	90
V/ 10^6 pm^3	250,86960

Overall displacement parameter:	0,000000
Extinction:	0,000000
Flat Plate Absorption Correction:	0,000000
Porosity:	0,000000
Roughness:	0,000000
Fitting mode:	Structure Fit
U Left:	0,000000
V Left:	0,000000
W Left:	0,010000
Preferred orientation direction/ hkl:	0,00 0,00 1,00
Preferred orientation parameter:	1,000000
Asymmetry parameter 1:	0,000000
Asymmetry parameter 2:	0,000000
Peak shape:	
parameter 1 Left:	0,600000
parameter 2 Left:	0,000000
parameter 3 Left:	0,000000
R (Bragg)/ %:	6,87881

Occupancy, atomic fract. coordinates and Biso for 74483-ICSD, ScPO4

Atom	Wyck.	s.o.f.	x	y	z	B/ 10^4 pm^2
Sc1	4a	1,000000	0,000000	0,750000	0,125000	0,000000
P1	4b	1,000000	0,000000	0,250000	0,375000	0,000000
O1	16h	1,000000	0,000000	0,068900	0,208400	0,378992

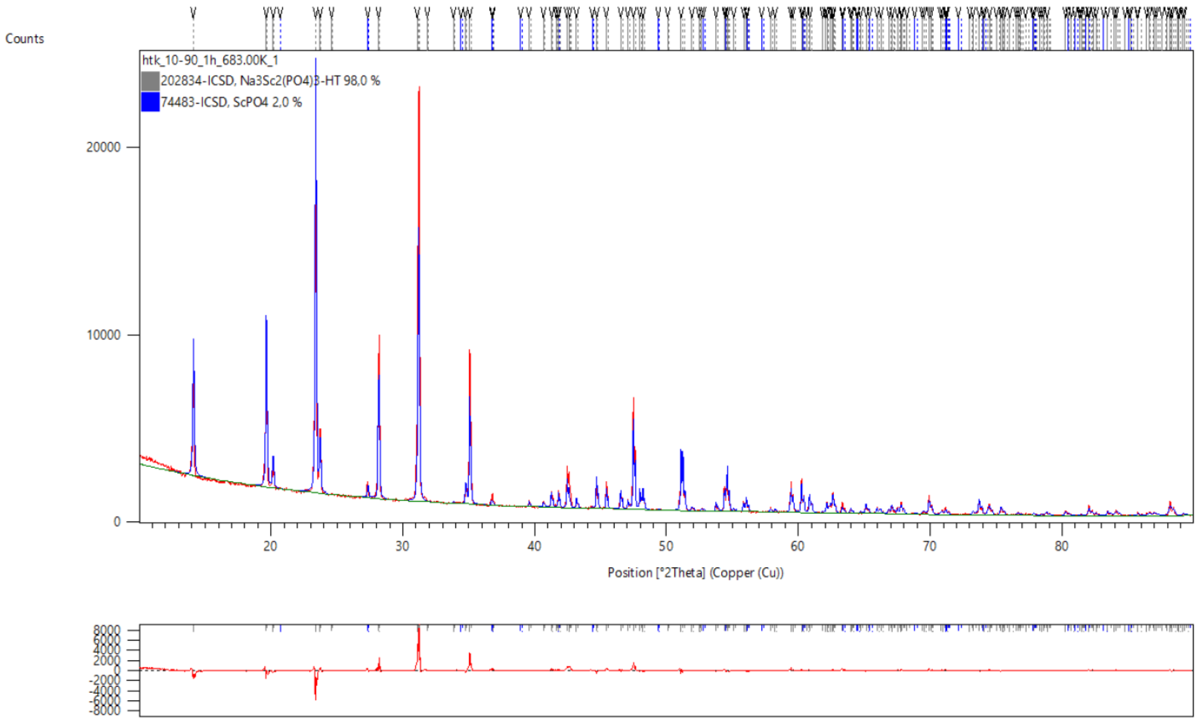

Figure S27. Rietveld refinement of XRD pattern of $Na_3Sc_2(PO_4)_3$:0.2%Eu^{3+} measured at 683K.

$Na_3Sc_2(PO_4)_3$:0.2%Eu^{3+} measured at 713K

Global Parameters

Number of used phases:	2
Number of variables:	14
Number of constraints:	1
Zero shift/ °2Theta:	0,000000

Specimen displacement/ mm :	-0,522(2)
Profile function:	Pseudo Voigt
Background:	Polynomial
R (expected)/ %:	2,86731
R (profile)/ %:	8,31556
R (weighted profile)/ %:	12,17369
GOF:	18,02586
d-statistic:	0,30668
U standard:	0,000000
V standard:	0,000000
W standard:	0,010000
U Left:	0,000000
V Left:	0,000000
W Left:	0,010000
U Right:	0,000000
V Right:	0,000000
W Right:	0,010000
Asymmetry Type:	No Asymmetry Function
Asymmetry 1:	0,000000
Asymmetry 2:	0,000000
Shape Type:	Shape Individual
Shape 1 Left:	0,600000
Shape 2 Left:	0,000000
Shape 3 Left:	0,000000
Shape 1 Right:	0,600000
Shape 2 Right:	0,000000
Shape 3 Right:	0,000000
K a1/a2 intensity ratio:	0,500000
K alpha/beta intensity ratio:	0,000000
Crystal Shape Factor K:	1,0000
Instrumental FWHM Curve Type:	Caglioti function
Instr. Gauss Curve Coefficient A:	0,0045(5)
Instr. Gauss Curve Coefficient B:	-0,0032(9)
Instr. Gauss Curve Coefficient C:	0,0046(3)
Instr. Lorentz Curve Coefficient A:	0,0062(7)
Instr. Lorentz Curve Coefficient B:	-0,004(1)
Instr. Lorentz Curve Coefficient C:	0,0064(5)

Relevant parameters of 202834-ICSD, Na3Sc2(PO4)3-HT

Structure and profile data:	
Formula sum:	$Sc_{12,00}P_{18,00}O_{72,00}Na_{7,16}$
Formula mass/ g/mol:	2413,6550
Density (calculated)/ g/cm^3	2,5555
F(000):	1176,8040
Weight fraction/ %:	98(1)
Space group (No.):	R -3 c (167)
Lattice parameters:	
a/ A:	8,9213(3)
b/ A:	8,9213(3)
c/ A:	22,751(1)
alpha/ °:	90
beta/ °:	90
gamma/ °:	120
V/ 10^6 pm^3	1568,16800
Overall displacement parameter:	0,000000
Extinction:	0,000000
Flat Plate Absorption Correction:	0,000000
Porosity:	0,000000
Roughness:	0,000000
Fitting mode:	Structure Fit
U Left:	0,15(1)
V Left:	-0,112(8)
W Left:	0,028(1)
Preferred orientation direction/ hkl:	0,00 0,00 1,00
Preferred orientation parameter:	1,000000
Asymmetry parameter 1:	0,000000
Asymmetry parameter 2:	0,000000
Peak shape:	
parameter 1 Left:	0,58(3)
parameter 2 Left:	0,000000
parameter 3 Left:	0,000000
R (Bragg)/ %:	14,23407

Occupancy, atomic fract. coordinates and Biso for 202834-ICSD, Na3Sc2(PO4)3-HT

Atom	Wyck.	s.o.f.	x	y	z	B/ 10^4 pm^2
Sc1	12c	1,000000	0,000000	0,000000	0,148700	0,000000
P1	18e	1,000000	0,295100	0,000000	0,250000	0,000000
O1	36f	1,000000	0,023500	0,209100	0,194600	0,000000
O2	36f	1,000000	0,192170	0,172830	0,088730	0,000000
Na1	18e	0,347000	0,637200	0,000000	0,250000	0,000000
Na2	6b	0,153000	0,000000	0,000000	0,000000	0,000000

Relevant parameters of 74483-ICSD, ScPO4

Structure and profile data:	
Formula sum:	$Sc_{4,00}P_{4,00}O_{16,00}$
Formula mass/ g/mol:	559,7092
Density (calculated)/ g/cm^3	3,7043
F(000):	272,0000
Weight fraction/ %:	1,8(2)
Space group (No.):	I 41/a m d (141)
Lattice parameters:	
a/ A:	6,579000
b/ A:	6,579000
c/ A:	5,796000
alpha/ °:	90
beta/ °:	90
gamma/ °:	90
V/ 10^6 pm^3	250,86960
Overall displacement parameter:	0,000000
Extinction:	0,000000
Flat Plate Absorption Correction:	0,000000
Porosity:	0,000000
Roughness:	0,000000
Fitting mode:	Structure Fit
U Left:	0,000000
V Left:	0,000000
W Left:	0,010000
Preferred orientation direction/ hkl:	0,00 0,00 1,00
Preferred orientation parameter:	1,000000
Asymmetry parameter 1:	0,000000
Asymmetry parameter 2:	0,000000
Peak shape:	
parameter 1 Left:	0,600000
parameter 2 Left:	0,000000
parameter 3 Left:	0,000000
R (Bragg)/ %:	7,67850

Occupancy, atomic fract. coordinates and Biso for 74483-ICSD, ScPO4

Atom	Wyck.	s.o.f.	x	y	z	B/ 10^4 pm^2
Sc1	4a	1,000000	0,000000	0,750000	0,125000	0,000000
P1	4b	1,000000	0,000000	0,250000	0,375000	0,000000
O1	16h	1,000000	0,000000	0,068900	0,208400	0,378992

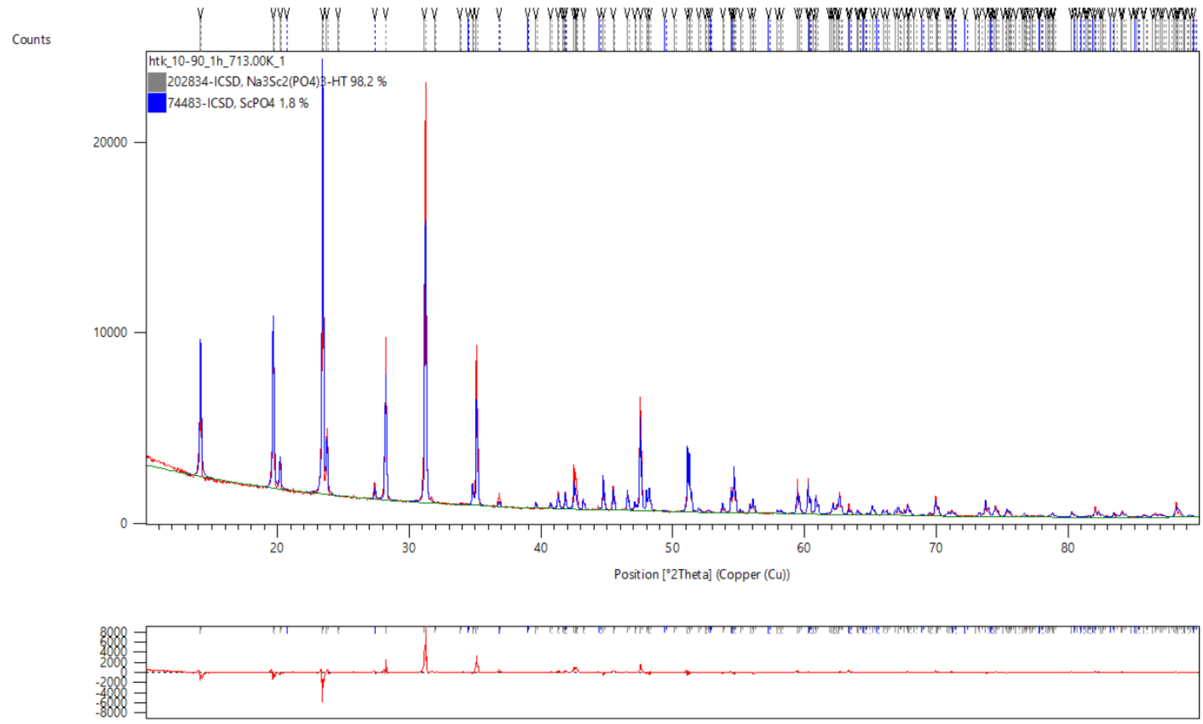

Figure S28. Rietveld refinement of XRD pattern of $Na_3Sc_2(PO_4)_3$:0.2%Eu^{3+} measured at 713K.

$Na_3Sc_2(PO_4)_3$:0.2%Eu^{3+} measured at 743K

Global Parameters

Number of used phases:	2
Number of variables:	14
Number of constraints:	1
Zero shift/ °2Theta:	0,000000
Specimen displacement/ mm :	-0,558(2)
Profile function:	Pseudo Voigt
Background:	Polynomial
R (expected)/ %:	2,86333
R (profile)/ %:	8,44162
R (weighted profile)/ %:	12,28327
GOF:	18,40290
d-statistic:	0,30766
U standard:	0,000000
V standard:	0,000000
W standard:	0,010000
U Left:	0,000000
V Left:	0,000000
W Left:	0,010000
U Right:	0,000000
V Right:	0,000000
W Right:	0,010000
Asymmetry Type:	No Asymmetry Function
Asymmetry 1:	0,000000
Asymmetry 2:	0,000000
Shape Type:	Shape Individual
Shape 1 Left:	0,600000
Shape 2 Left:	0,000000
Shape 3 Left:	0,000000
Shape 1 Right:	0,600000
Shape 2 Right:	0,000000
Shape 3 Right:	0,000000
K a1/a2 intensity ratio:	0,500000
K alpha/beta intensity ratio:	0,000000
Crystal Shape Factor K:	1,0000

Instrumental FWHM Curve Type:	Caglioti function
Instr. Gauss Curve Coefficient A:	0,0045(5)
Instr. Gauss Curve Coefficient B:	-0,0032(9)
Instr. Gauss Curve Coefficient C:	0,0046(3)
Instr. Lorentz Curve Coefficient A:	0,0062(7)
Instr. Lorentz Curve Coefficient B:	-0,004(1)
Instr. Lorentz Curve Coefficient C:	0,0064(5)

Relevant parameters of 202834-ICSD, Na3Sc2(PO4)3-HT

Structure and profile data:	
Formula sum:	$Sc_{12,00}P_{18,00}O_{72,00}Na_{7,16}$
Formula mass/ g/mol:	2413,6550
Density (calculated)/ g/cm^3	2,5541
F(000):	1176,8040
Weight fraction/ %:	98(1)
Space group (No.):	R -3 c (167)
Lattice parameters:	
a/ A:	8,9214(3)
b/ A:	8,9214(3)
c/ A:	22,763(1)
alpha/ °:	90
beta/ °:	90
gamma/ °:	120
V/ 10^6 pm^3	1569,01100
Overall displacement parameter:	0,000000
Extinction:	0,000000
Flat Plate Absorption Correction:	0,000000
Porosity:	0,000000
Roughness:	0,000000
Fitting mode:	Structure Fit
U Left:	0,16(1)
V Left:	-0,123(8)
W Left:	0,029(1)
Preferred orientation direction/ hkl:	0,00 0,00 1,00
Preferred orientation parameter:	1,000000
Asymmetry parameter 1:	0,000000
Asymmetry parameter 2:	0,000000
Peak shape:	
parameter 1 Left:	0,58(3)
parameter 2 Left:	0,000000
parameter 3 Left:	0,000000
R (Bragg)/ %:	14,17314

Occupancy, atomic fract. coordinates and Biso for 202834-ICSD, Na3Sc2(PO4)3-HT

Atom	Wyck.	s.o.f.	x	y	z	B/ 10^4 pm^2
Sc1	12c	1,000000	0,000000	0,000000	0,148700	0,000000
P1	18e	1,000000	0,295100	0,000000	0,250000	0,000000
O1	36f	1,000000	0,023500	0,209100	0,194600	0,000000
O2	36f	1,000000	0,192170	0,172830	0,088730	0,000000
Na1	18e	0,347000	0,637200	0,000000	0,250000	0,000000
Na2	6b	0,153000	0,000000	0,000000	0,000000	0,000000

Relevant parameters of 74483-ICSD, ScPO4

Structure and profile data:	
Formula sum:	$Sc_{4,00}P_{4,00}O_{16,00}$
Formula mass/ g/mol:	559,7092
Density (calculated)/ g/cm^3	3,7043
F(000):	272,0000
Weight fraction/ %:	2,0(2)
Space group (No.):	I 41/a m d (141)
Lattice parameters:	
a/ A:	6,579000
b/ A:	6,579000
c/ A:	5,796000
alpha/ °:	90
beta/ °:	90
gamma/ °:	90
V/ 10^6 pm^3	250,86960

Overall displacement parameter:	0,000000
Extinction:	0,000000
Flat Plate Absorption Correction:	0,000000
Porosity:	0,000000
Roughness:	0,000000
Fitting mode:	Structure Fit
U Left:	0,000000
V Left:	0,000000
W Left:	0,010000
Preferred orientation direction/ hkl:	0,00 0,00 1,00
Preferred orientation parameter:	1,000000
Asymmetry parameter 1:	0,000000
Asymmetry parameter 2:	0,000000
Peak shape:	
parameter 1 Left:	0,600000
parameter 2 Left:	0,000000
parameter 3 Left:	0,000000
R (Bragg)/ %:	7,31326

Occupancy, atomic fract. coordinates and Biso for 74483-ICSD, ScPO4

Atom	Wyck.	s.o.f.	x	y	z	B/ 10^4 pm^2
Sc1	4a	1,000000	0,000000	0,750000	0,125000	0,000000
P1	4b	1,000000	0,000000	0,250000	0,375000	0,000000
O1	16h	1,000000	0,000000	0,068900	0,208400	0,378992

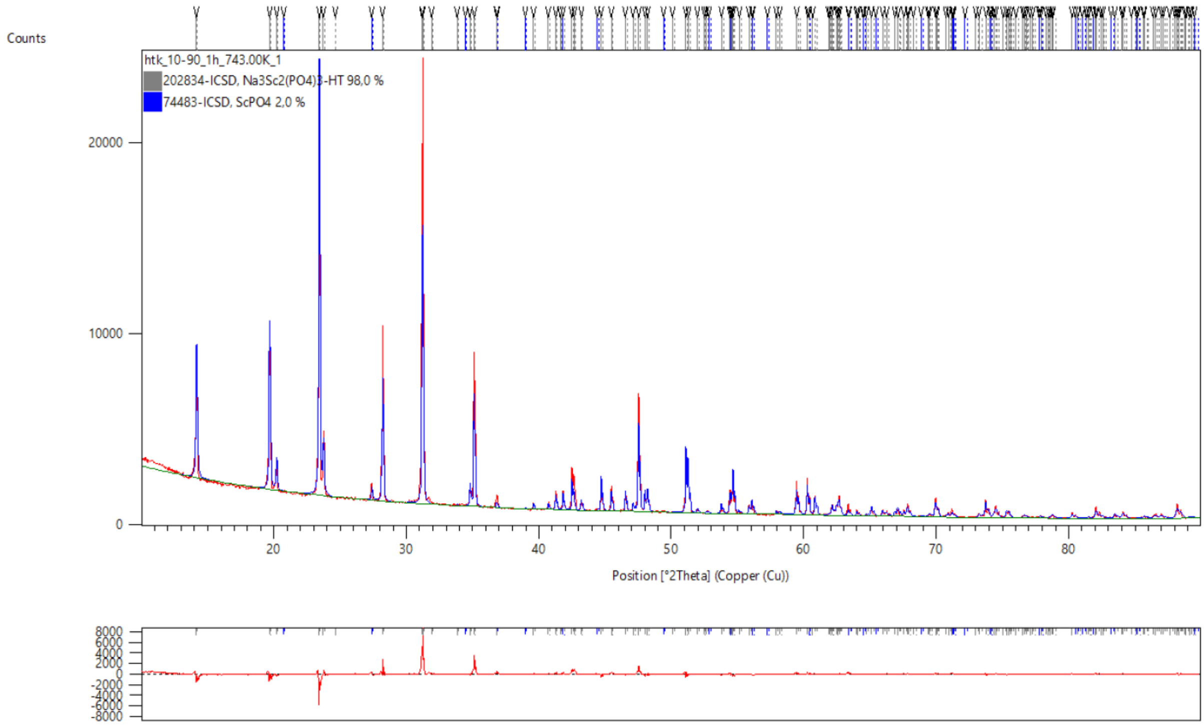

Figure S29. Rietveld refinement of XRD pattern of $Na_3Sc_2(PO_4)_3$:0.2%Eu^{3+} measured at 743K.

$Na_3Sc_2(PO_4)_3$:0.2%Eu^{3+} measured at 773K

Global Parameters

Number of used phases:	2
Number of variables:	14

Number of constraints:	1
Zero shift/ °2Theta:	0,000000
Specimen displacement/ mm :	-0,467(2)
Profile function:	Pseudo Voigt
Background:	Polynomial
R (expected)/ %:	2,87450
R (profile)/ %:	8,41188
R (weighted profile)/ %:	12,19360
GOF:	17,99448
d-statistic:	0,31654
U standard:	0,000000
V standard:	0,000000
W standard:	0,010000
U Left:	0,000000
V Left:	0,000000
W Left:	0,010000
U Right:	0,000000
V Right:	0,000000
W Right:	0,010000
Asymmetry Type:	No Asymmetry Function
Asymmetry 1:	0,000000
Asymmetry 2:	0,000000
Shape Type:	Shape Individual
Shape 1 Left:	0,600000
Shape 2 Left:	0,000000
Shape 3 Left:	0,000000
Shape 1 Right:	0,600000
Shape 2 Right:	0,000000
Shape 3 Right:	0,000000
K a1/a2 intensity ratio:	0,500000
K alpha/beta intensity ratio:	0,000000
Crystal Shape Factor K:	1,0000
Instrumental FWHM Curve Type:	Caglioti function
Instr. Gauss Curve Coefficient A:	0,0045(5)
Instr. Gauss Curve Coefficient B:	-0,0032(9)
Instr. Gauss Curve Coefficient C:	0,0046(3)
Instr. Lorentz Curve Coefficient A:	0,0062(7)
Instr. Lorentz Curve Coefficient B:	-0,004(1)
Instr. Lorentz Curve Coefficient C:	0,0064(5)

Relevant parameters of 202834-ICSD, Na3Sc2(PO4)3-HT

Structure and profile data:	
Formula sum:	$Sc_{12,00}P_{18,00}O_{72,00}Na_{7,16}$
Formula mass/ g/mol:	2413,6550
Density (calculated)/ g/cm^3	2,5510
F(000):	1176,8040
Weight fraction/ %:	98(1)
Space group (No.):	R -3 c (167)
Lattice parameters:	
a/ A:	8,9238(3)
b/ A:	8,9238(3)
c/ A:	22,7783(9)
alpha/ °:	90
beta/ °:	90
gamma/ °:	120
V/ 10^6 pm^3	1570,89900
Overall displacement parameter:	0,000000
Extinction:	0,000000
Flat Plate Absorption Correction:	0,000000
Porosity:	0,000000
Roughness:	0,000000
Fitting mode:	Structure Fit
U Left:	0,14(1)
V Left:	-0,109(8)
W Left:	0,027(1)
Preferred orientation direction/ hkl:	0,00 0,00 1,00
Preferred orientation parameter:	1,000000
Asymmetry parameter 1:	0,000000
Asymmetry parameter 2:	0,000000
Peak shape:	
parameter 1 Left:	0,61(3)
parameter 2 Left:	0,000000
parameter 3 Left:	0,000000
R (Bragg)/ %:	14,05594

Occupancy, atomic fract. coordinates and Biso for 202834-ICSD, Na3Sc2(PO4)3-HT

Atom	Wyck.	s.o.f.	x	y	z	B/ 10^4 pm^2
Sc1	12c	1,000000	0,000000	0,000000	0,148700	0,000000
P1	18e	1,000000	0,295100	0,000000	0,250000	0,000000
O1	36f	1,000000	0,023500	0,209100	0,194600	0,000000
O2	36f	1,000000	0,192170	0,172830	0,088730	0,000000
Na1	18e	0,347000	0,637200	0,000000	0,250000	0,000000
Na2	6b	0,153000	0,000000	0,000000	0,000000	0,000000

Relevant parameters of 74483-ICSD, ScPO4

Structure and profile data:	
Formula sum:	$Sc_{4,00}P_{4,00}O_{16,00}$
Formula mass/ g/mol:	559,7092
Density (calculated)/ g/cm^3	3,7043
F(000):	272,0000
Weight fraction/ %:	1,7(2)
Space group (No.):	I 41/a m d (141)
Lattice parameters:	
a/ A:	6,579000
b/ A:	6,579000
c/ A:	5,796000
alpha/ °:	90
beta/ °:	90
gamma/ °:	90
V/ 10^6 pm^3	250,86960
Overall displacement parameter:	0,000000
Extinction:	0,000000
Flat Plate Absorption Correction:	0,000000
Porosity:	0,000000
Roughness:	0,000000
Fitting mode:	Structure Fit
U Left:	0,000000
V Left:	0,000000
W Left:	0,010000
Preferred orientation direction/ hkl:	0,00 0,00 1,00
Preferred orientation parameter:	1,000000
Asymmetry parameter 1:	0,000000
Asymmetry parameter 2:	0,000000
Peak shape:	
parameter 1 Left:	0,600000
parameter 2 Left:	0,000000
parameter 3 Left:	0,000000
R (Bragg)/ %:	7,44401

Occupancy, atomic fract. coordinates and Biso for 74483-ICSD, ScPO4

Atom	Wyck.	s.o.f.	x	y	z	B/ 10^4 pm^2
Sc1	4a	1,000000	0,000000	0,750000	0,125000	0,000000
P1	4b	1,000000	0,000000	0,250000	0,375000	0,000000
O1	16h	1,000000	0,000000	0,068900	0,208400	0,378992

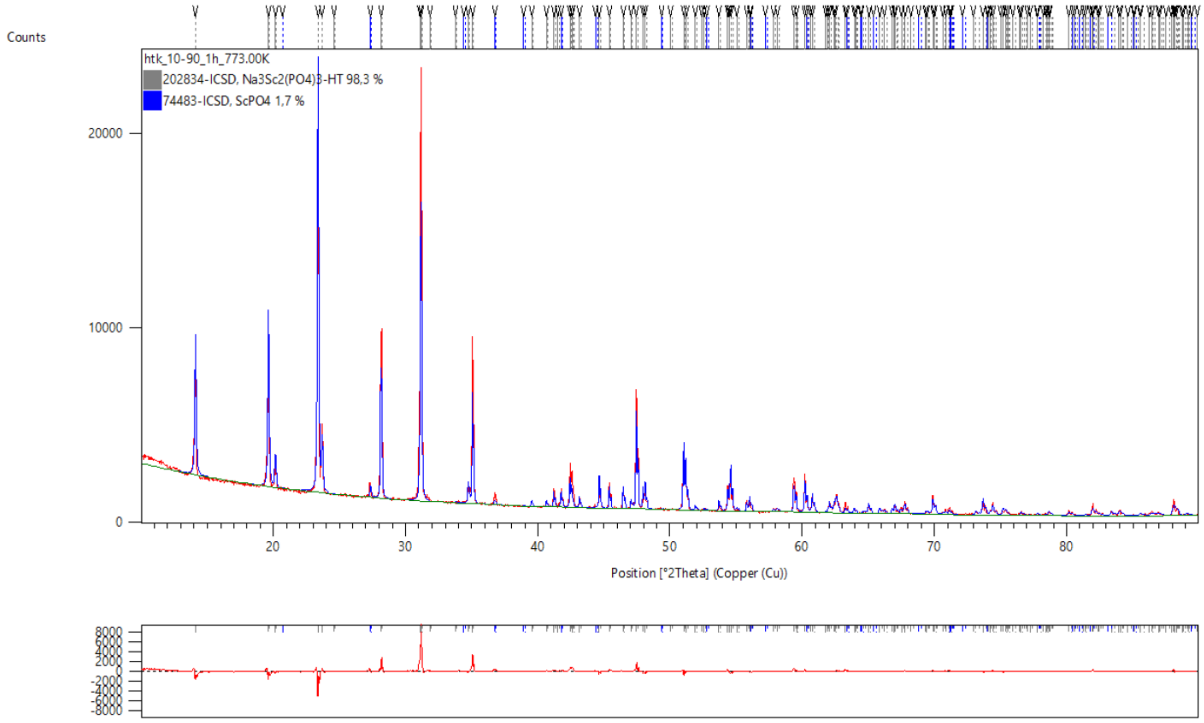

Figure S30. Rietveld refinement of XRD pattern of $Na_3Sc_2(PO_4)_3$:0.2%Eu^{3+} measured at 773K.

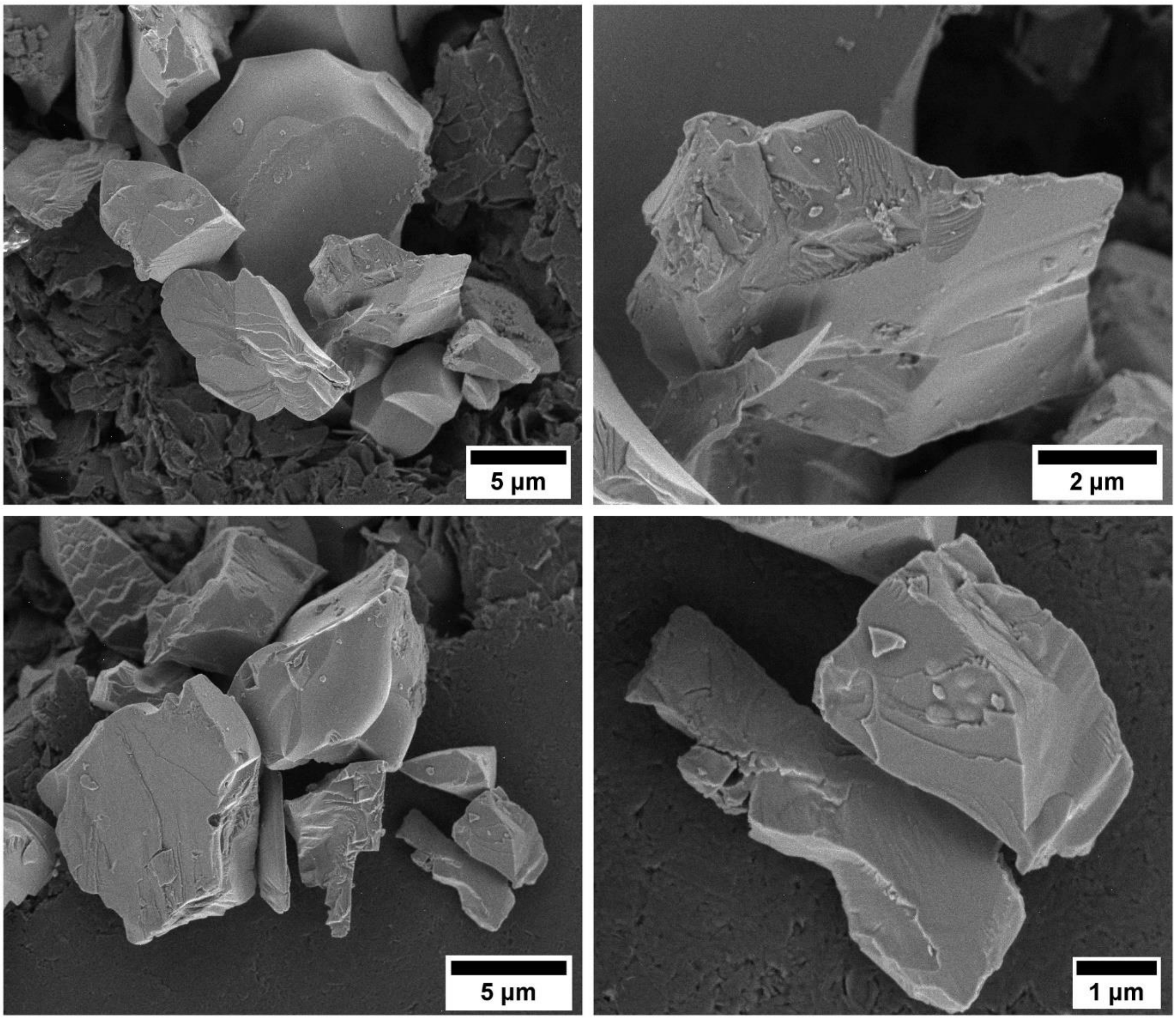

Figure S31. SEM images of $Na_3Sc_2(PO_4)_3$:0.2%Eu^{3+}.

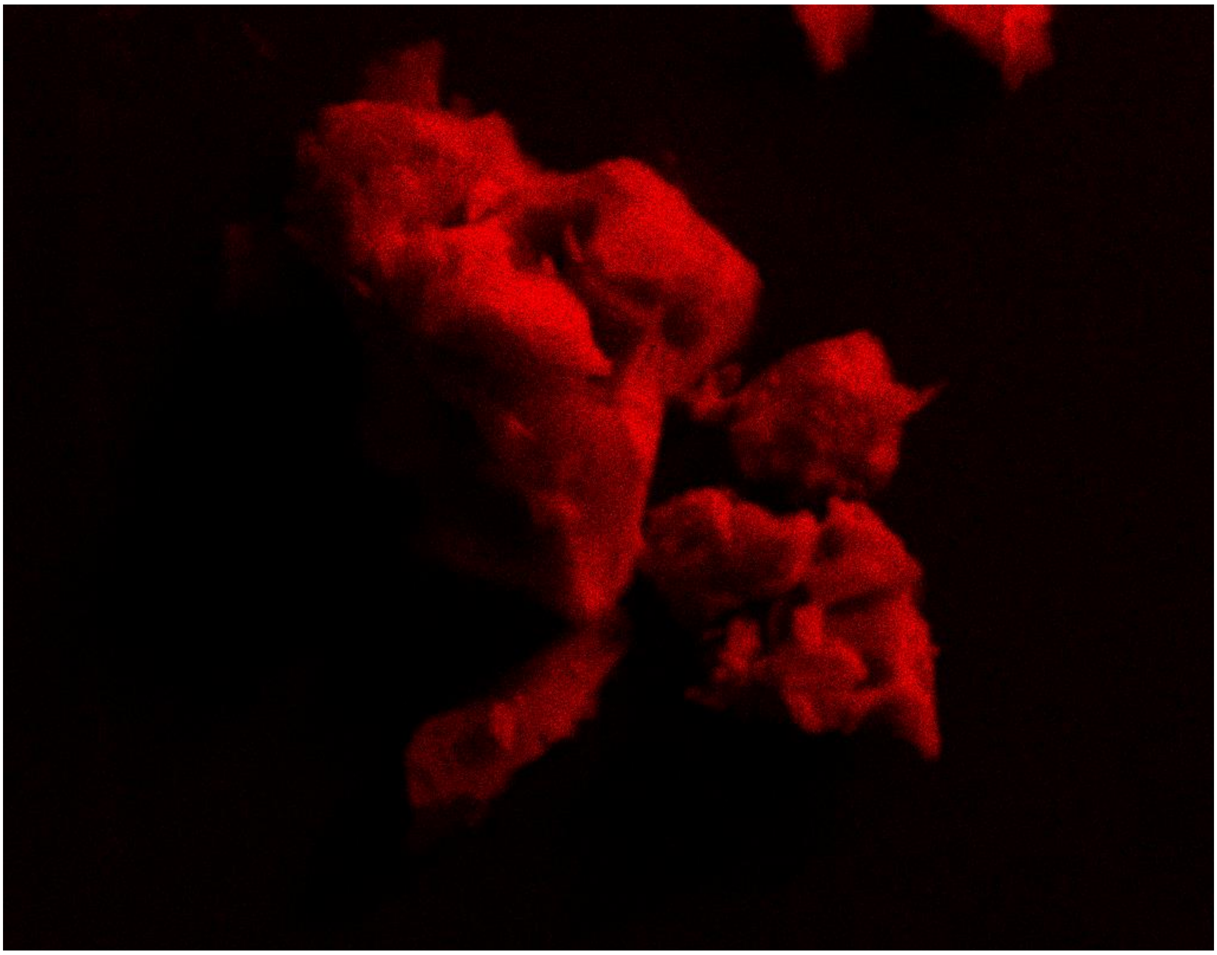

Figure S32. The elemental maps of O in the $Na_3Sc_2(PO_4)_3$:0.2%Eu^{3+} sample.

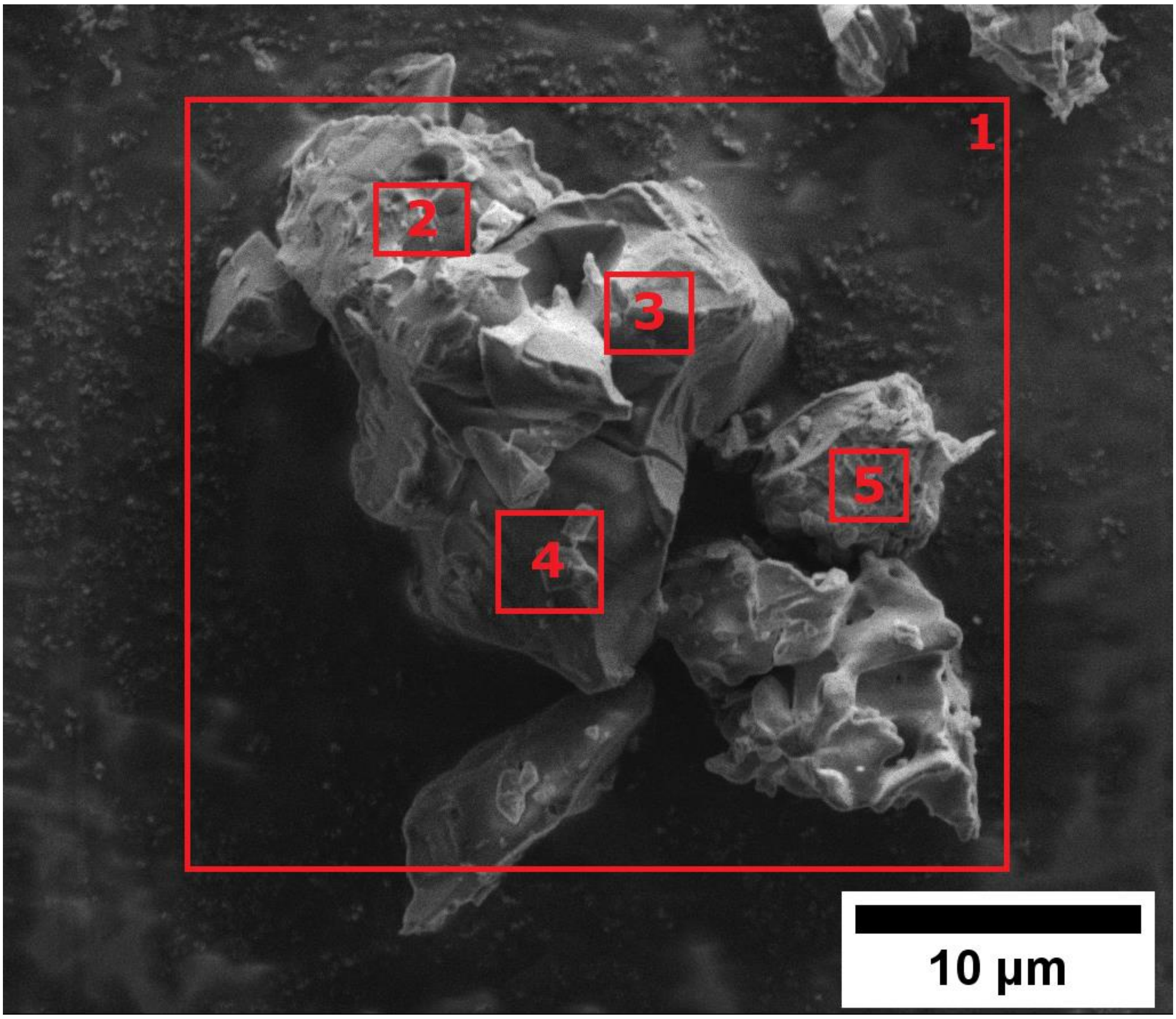

Figure S33. Regions of SEM image from which chemical elements for $Na_3Sc_2(PO_4)_3$:0.2%Eu^{3+} were analyzed.

Table S1. Average atomic content of the chemical elements for $Na_3Sc_2(PO_4)_3$:0.2%Eu^{3+} based on EDS results.

**No.**	**Chemical composition (at. %)(*)**				
	**Na**	**Sc**	**P**	**O**	**Eu**
1	14.1	11.0	14.9	59.9	0.1
2	15.5	10.2	15.5	58.7	0.1
3	15.4	10.1	15.7	58.7	0.1
4	15.2	11.2	14.2	59.3	0.1
5	15.2	10.9	15.5	58.3	0.1
Theoretical	15.0	10.0	15.0	60.0	<0.1

(*) The relative errors of EDS method are less than 2 %, 4 % and 50% for main (above 20 at. %), major (20 - 5. at. %) and trace (1 - 0.1) elements, respectively [D. E. Newbury, N. W. M. Ritchie, J Mater Sci 50 (2015) 493–518]

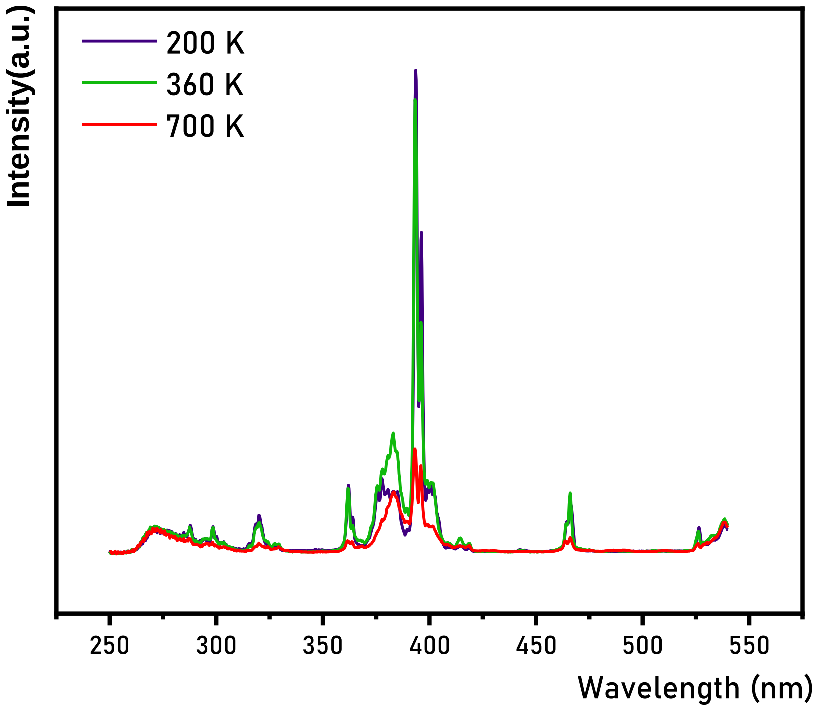

Figure S34. Comparison of excitation spectra of $Na_3Sc_2(PO_4)_3$:0.2%Eu^{3+} measured at three different temperatures (λ_{em}=612 nm).

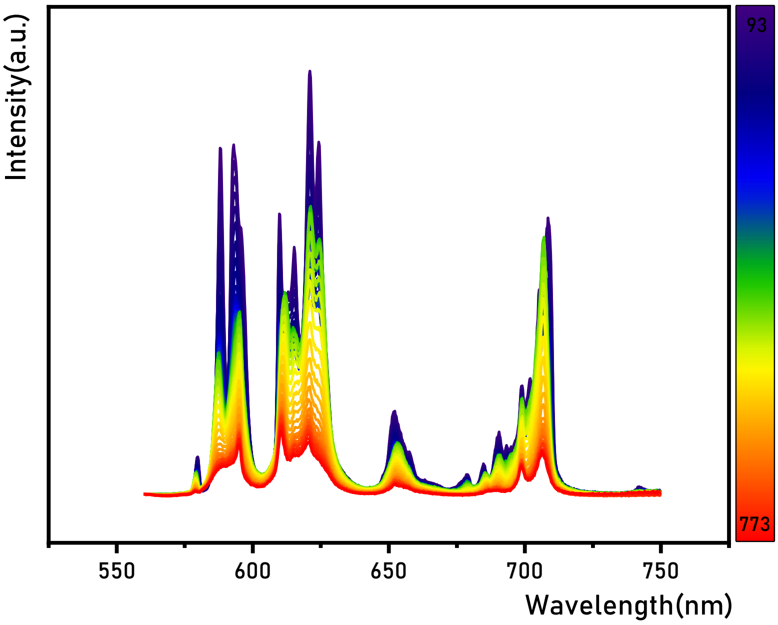

Figure S35. Emission spectra of $Na_3Sc_2(PO_4)_3$:0.2%Eu^{3+} measured as a function of temperature.

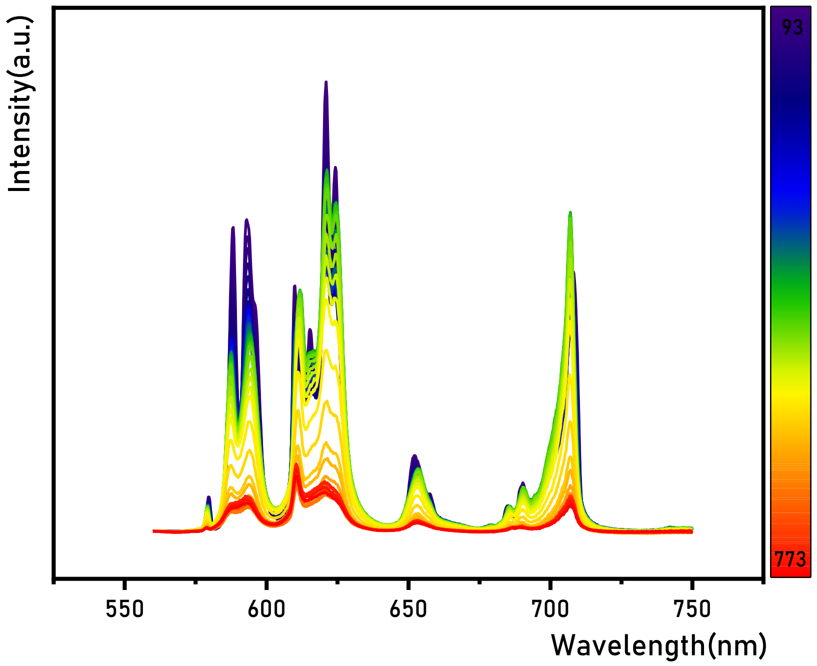

Figure S36. Emission spectra of $Na_3Sc_2(PO_4)_3$:0.3%Eu^{3+} measured as a function of temperature.

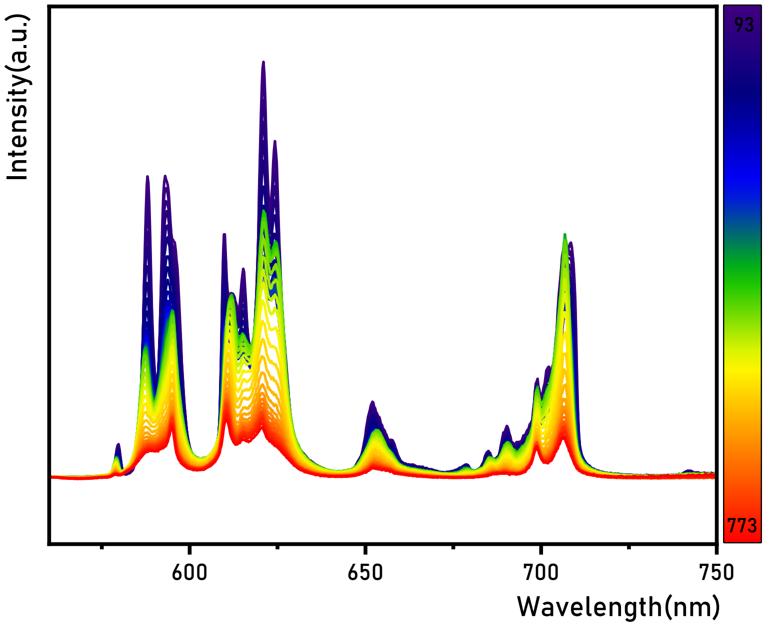

Figure S37. Emission spectra of $Na_3Sc_2(PO_4)_3$:0.4%Eu^{3+} measured as a function of temperature.

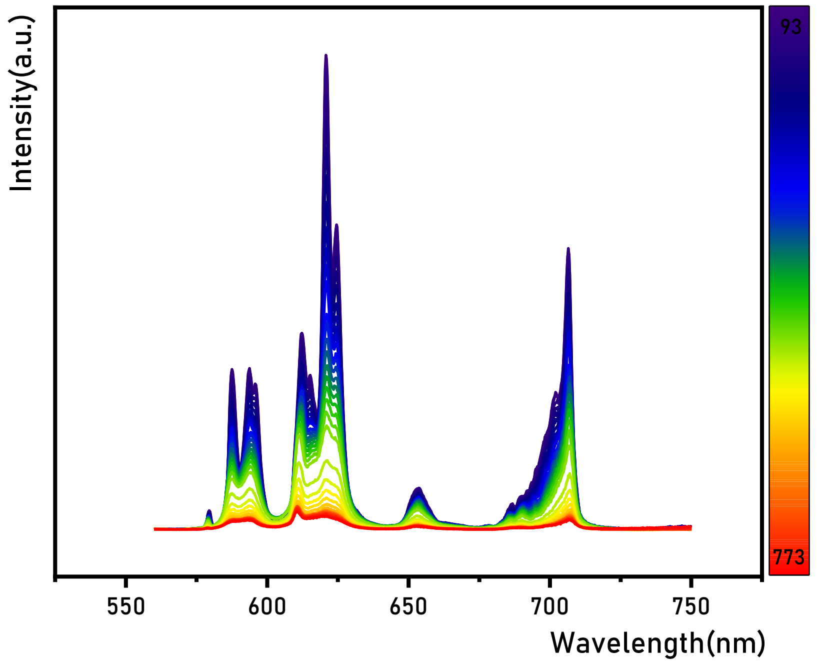

Figure S38. Emission spectra of $Na_3Sc_2(PO_4)_3$:0.5%Eu^{3+} measured as a function of temperature.

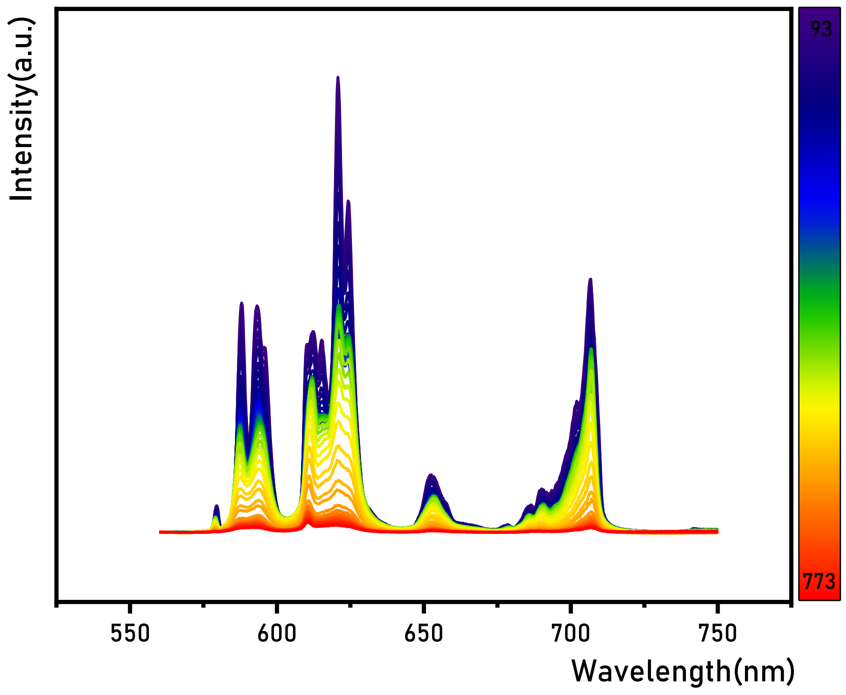

Figure S39. Emission spectra of $Na_3Sc_2(PO_4)_3$:1%Eu^{3+} measured as a function of temperature.

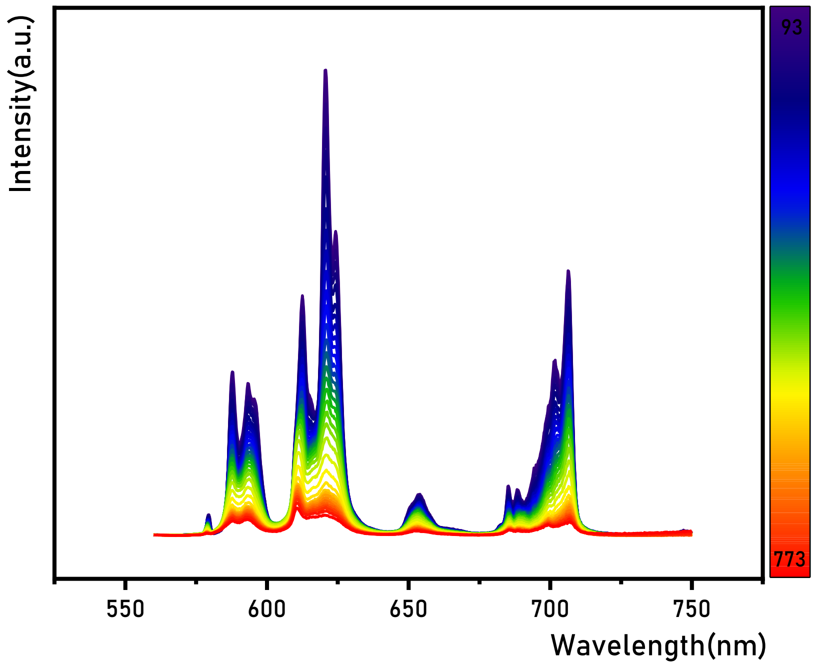

Figure S40. Emission spectra of $Na_3Sc_2(PO_4)_3$:5%Eu^{3+} measured as a function of temperature.

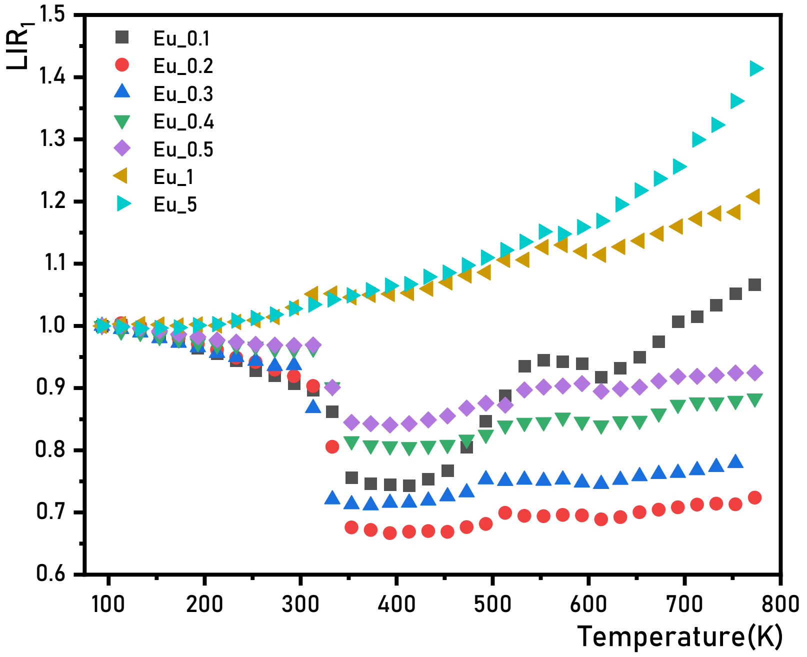

Figure S41. Thermal dependence of LIR_1 for different concentrations of Eu^{3+} ions.

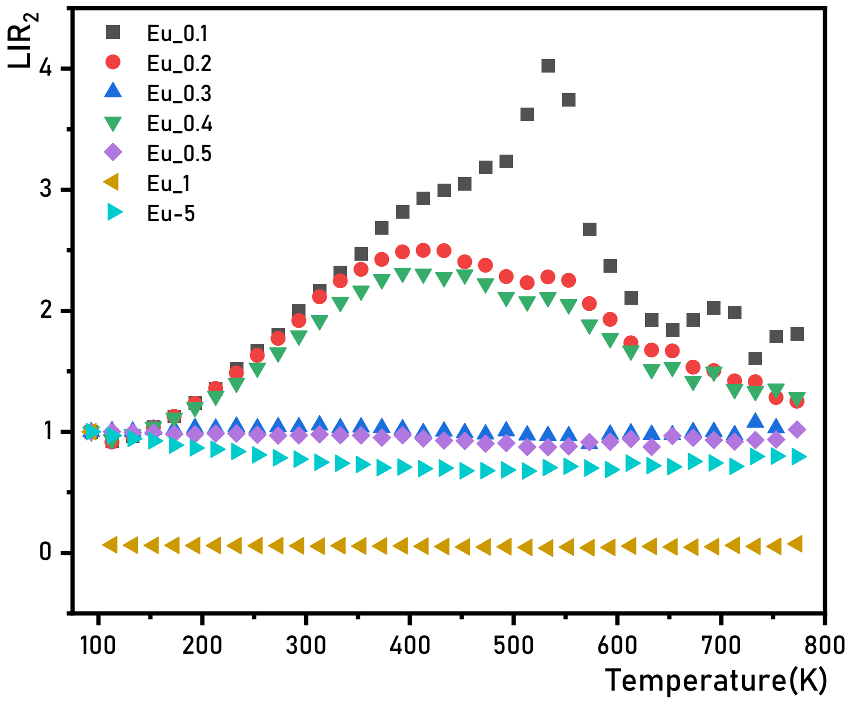

Figure S42. Thermal dependence of LIR_2 for different concentrations of Eu^{3+} ions.

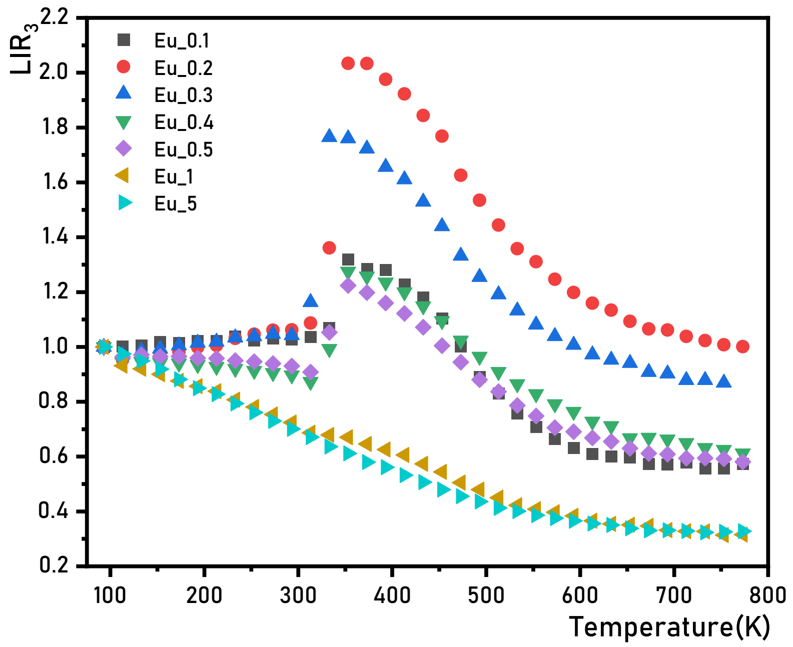

Figure S43. Thermal dependence of LIR_3 for different concentrations of Eu^{3+} ions.

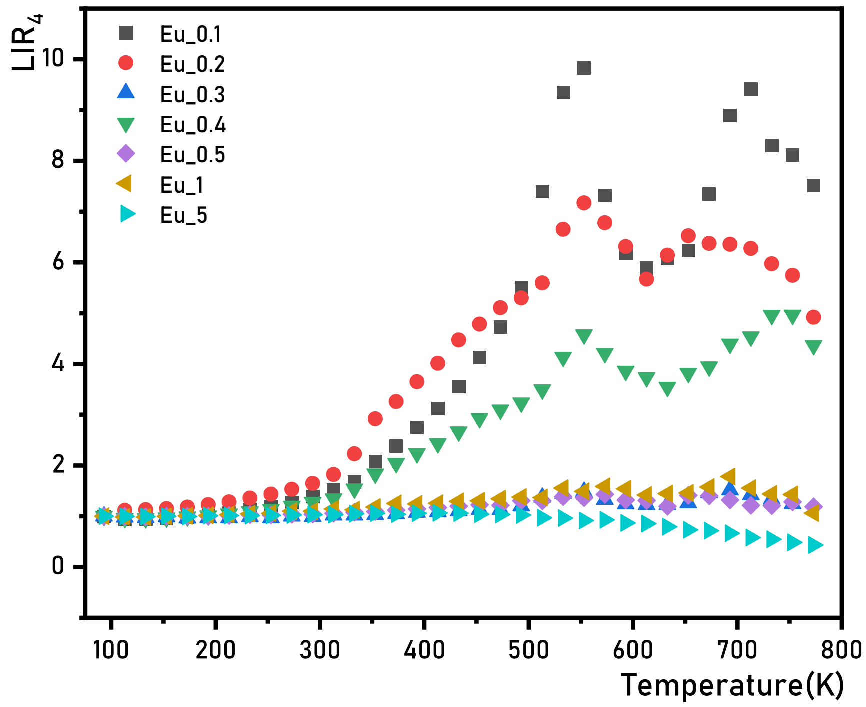

Figure S44. Thermal dependence of LIR_4 for different concentrations of Eu^{3+} ions.

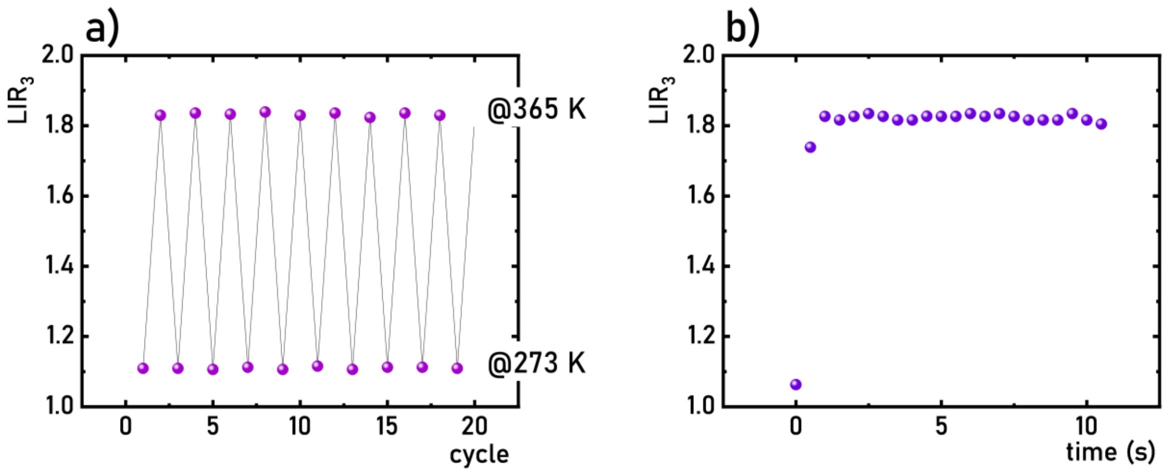

Figure S45. The LIR_3 value for $Na_3Sc_2(PO_4)_3$:0.3%Eu^{3+} measured within heating-cooling cycles -a) and temporal dependence of the same parameter exposed to 365 K-b).

Thermal dependence of LIR and τ_{avr} were fitted using the following formula:

$$\Omega(T) = A_1 + \frac{A_2 - A_1}{1 + 10^{((\log x) - T)p}} \qquad \text{(S1)}$$

where Ω is the τ_{avr} or LIR.

Representative values of fitting parameters for $Na_3Sc_2(PO_4)_3$:0.3%Eu^{3+} are given below:

Table S2. Fitting parameter for calibration curves of $Na_3Sc_2(PO_4)_3$:0.3%Eu^{3+} in LIR and lifetime based approaches

Approach	A1	A2	logx	p	R^2
LIR	0.95176±0.20206	1.87982±0.21756	221.310404±0.29198	0.18904±0.03906	0.99786
τ_{avr}	2.35522±0.02387	3.35597±0.02719	251.16922±13.16853	0.4891±0.03974	0.99762

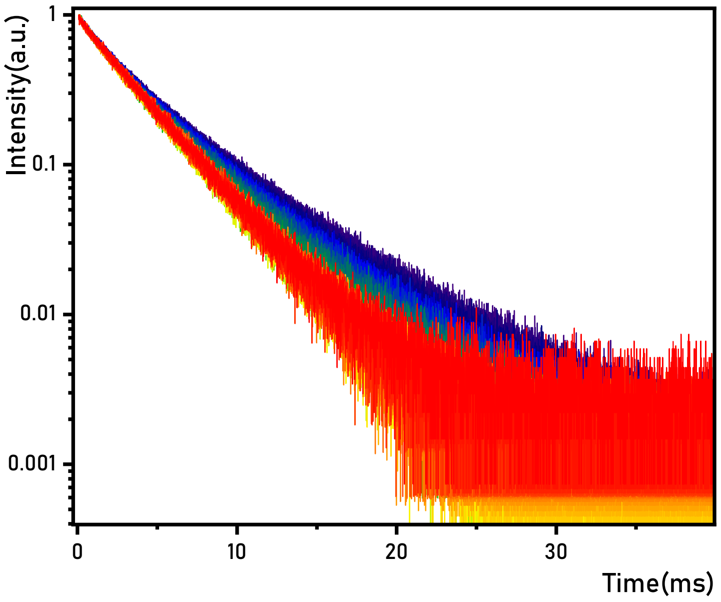

Figure S46. Luminescence decay profiles of $Na_3Sc_2(PO_4)_3$:0.2%Eu^{3+} measured as a function of temperature.

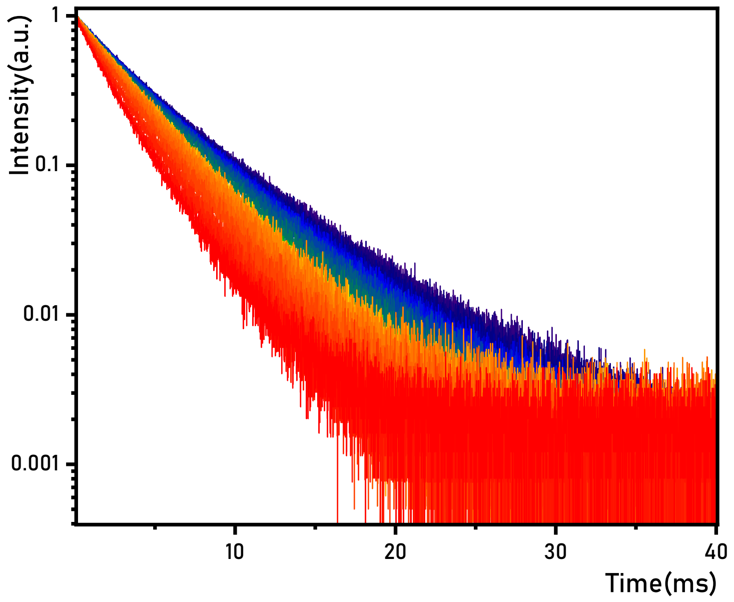

Figure S47. Luminescence decay profiles of $Na_3Sc_2(PO_4)_3$:0.3%Eu^{3+} measured as a function of temperature.

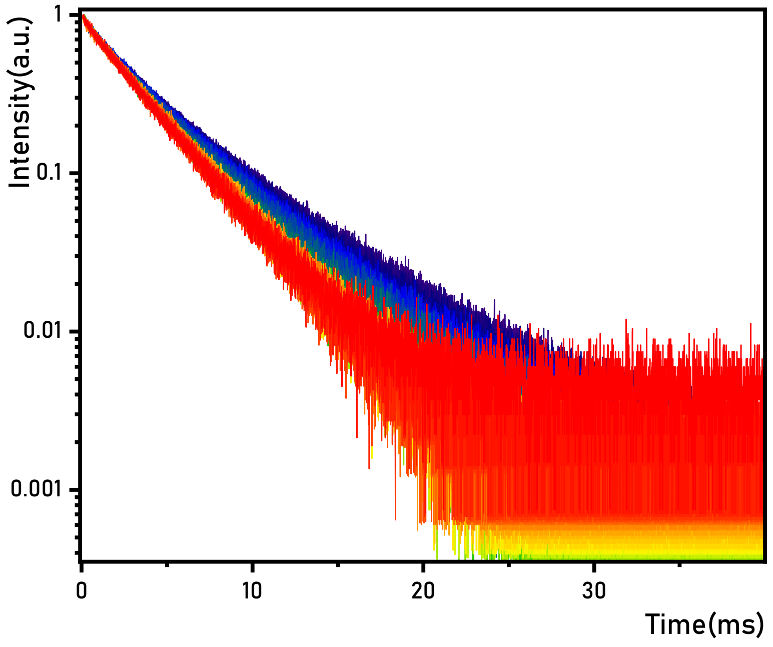

Figure S48. Luminescence decay profiles of $Na_3Sc_2(PO_4)_3$:0.4%Eu^{3+} measured as a function of temperature.

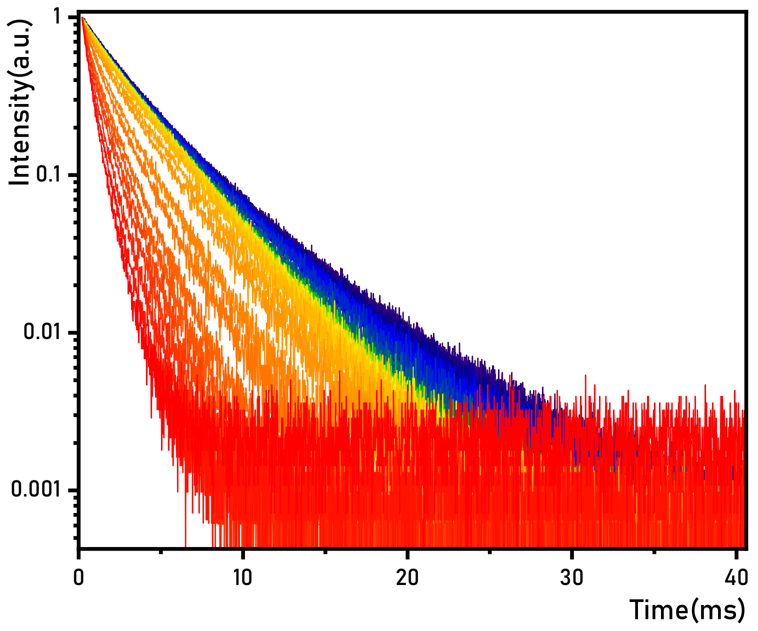

Figure S49. Luminescence decay profiles of $Na_3Sc_2(PO_4)_3$:0.5%Eu^{3+} measured as a function of temperature.

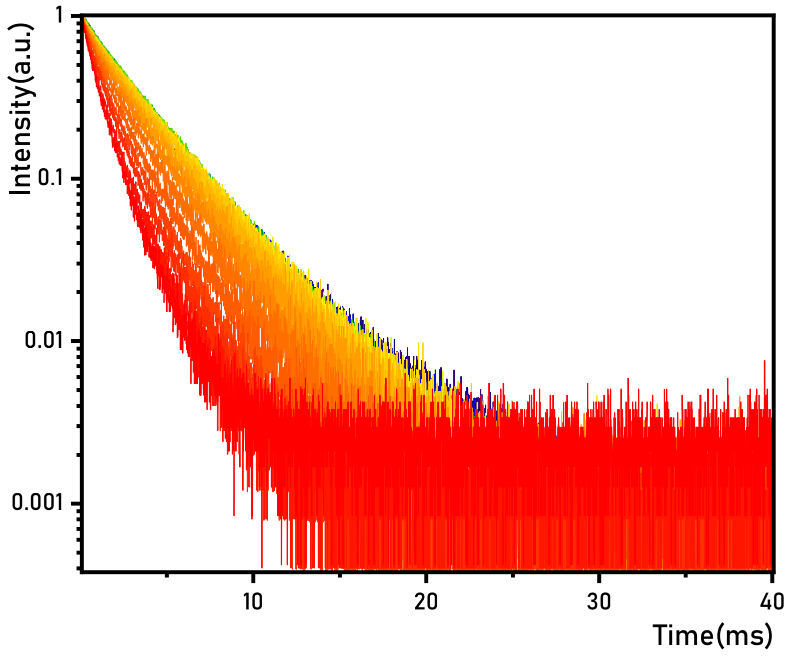

Figure S50. Luminescence decay profiles of $Na_3Sc_2(PO_4)_3$:1%Eu^{3+} measured as a function of temperature.

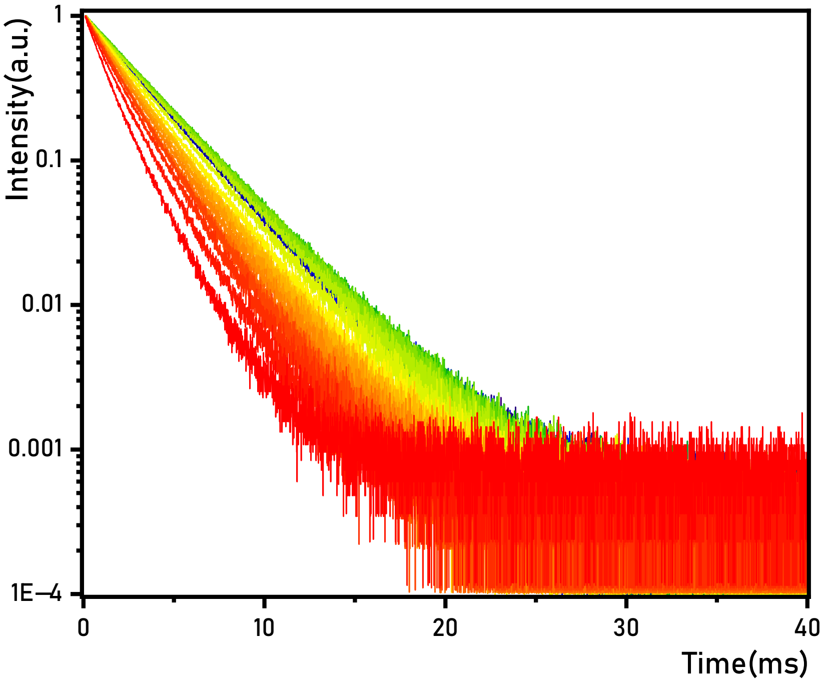

Figure S51. Luminescence decay profiles of $Na_3Sc_2(PO_4)_3$:5%Eu^{3+} measured as a function of temperature.

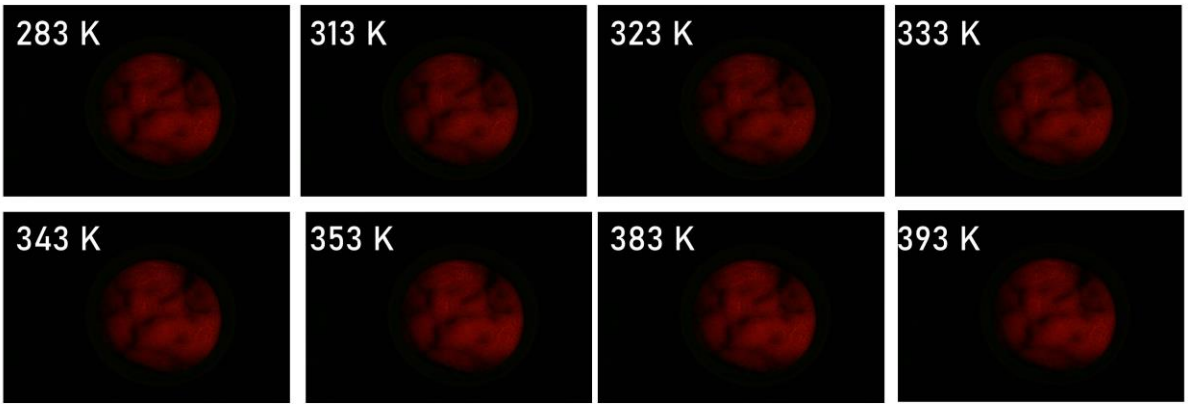

Figure S52. Photos of light emitted by $Na_3Sc_2(PO_4)_3$:0.2%Eu^{3+} at different temperatures.

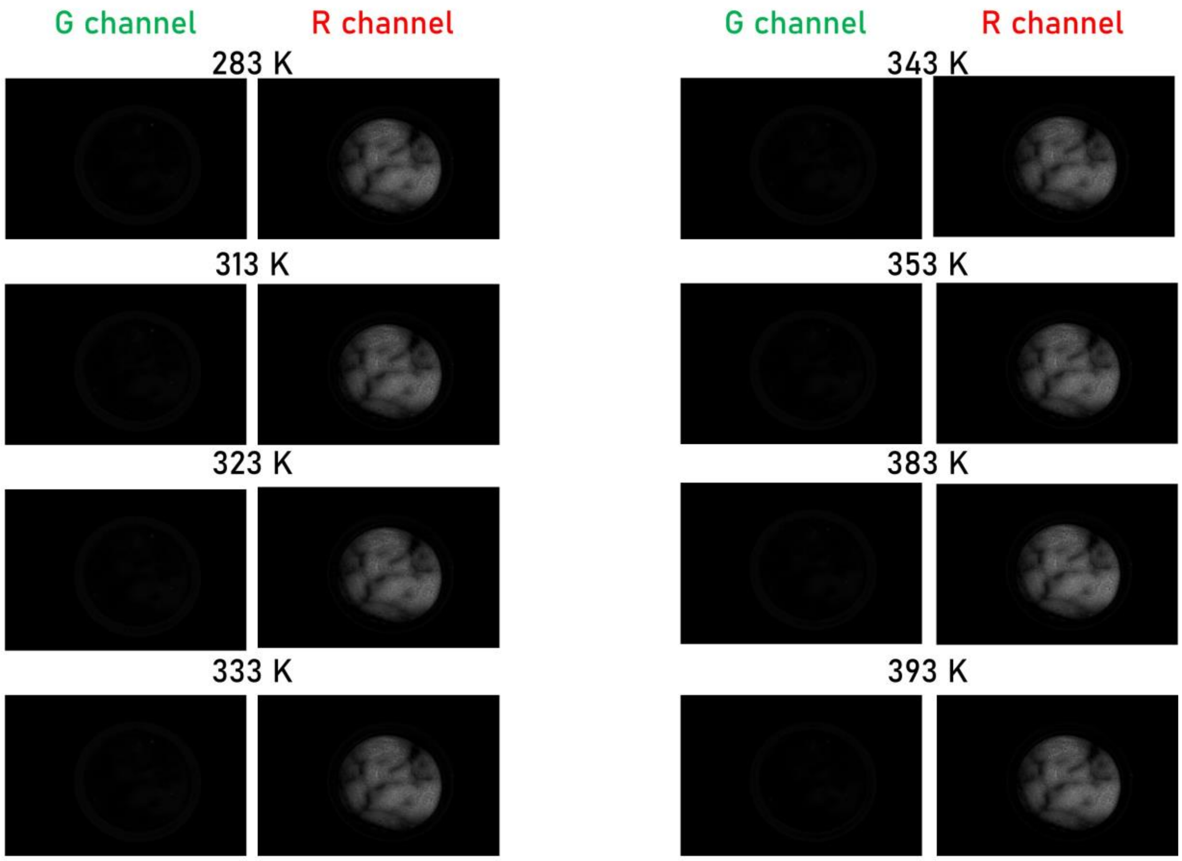

Figure S53. Intensities recorded in green (G) and red (R) channels of the digital camera extracted from photos presented in Figure S49.

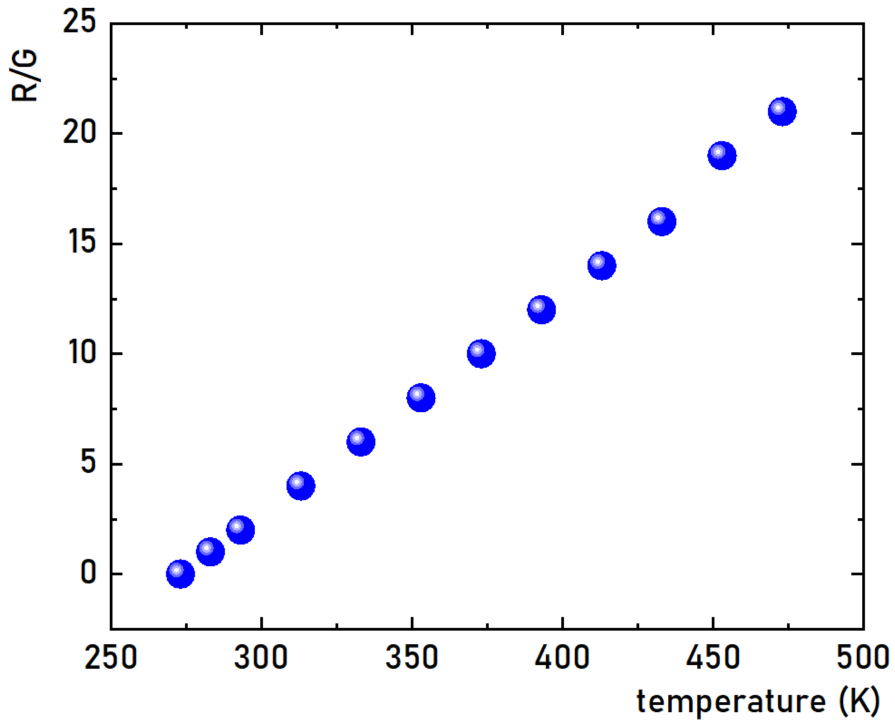

Figure S54. Calibration curve used in the thermal imaging experiment: the influence of temperature on the R/G ratio.

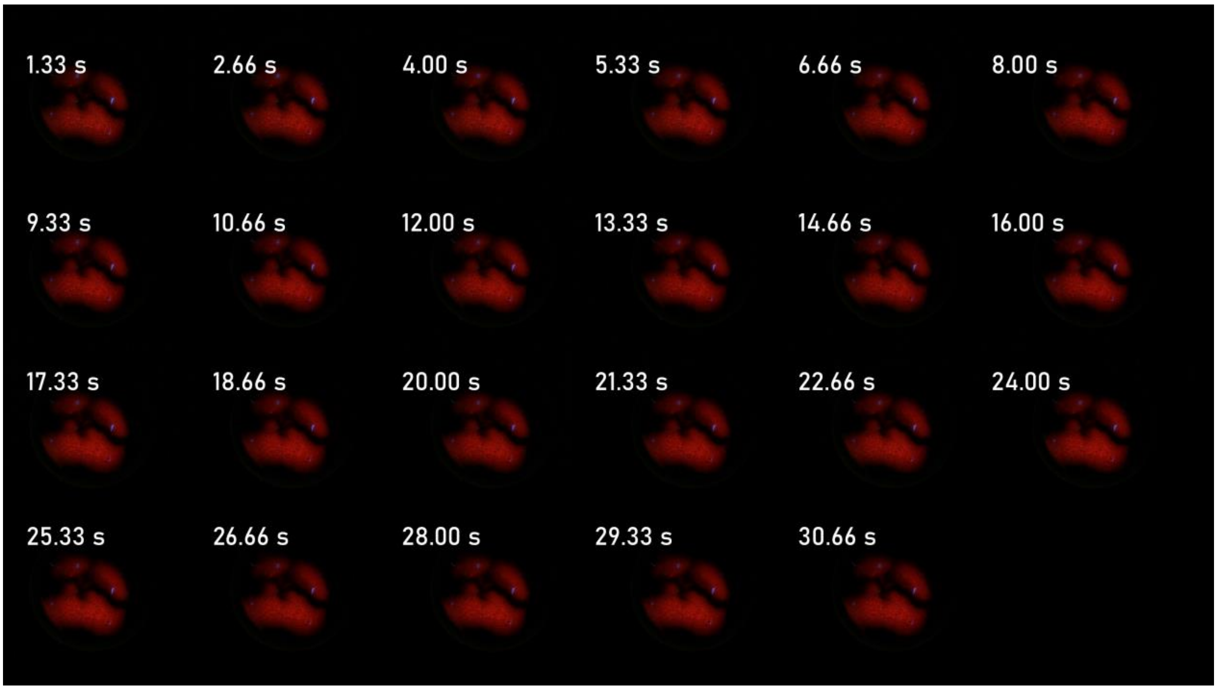

Figure S55. Photos of luminescence obtained using digital camera of analyzed luminescence thermometer in the experimental setup captured as a function of time after placing the quartz holder on the heating stage.

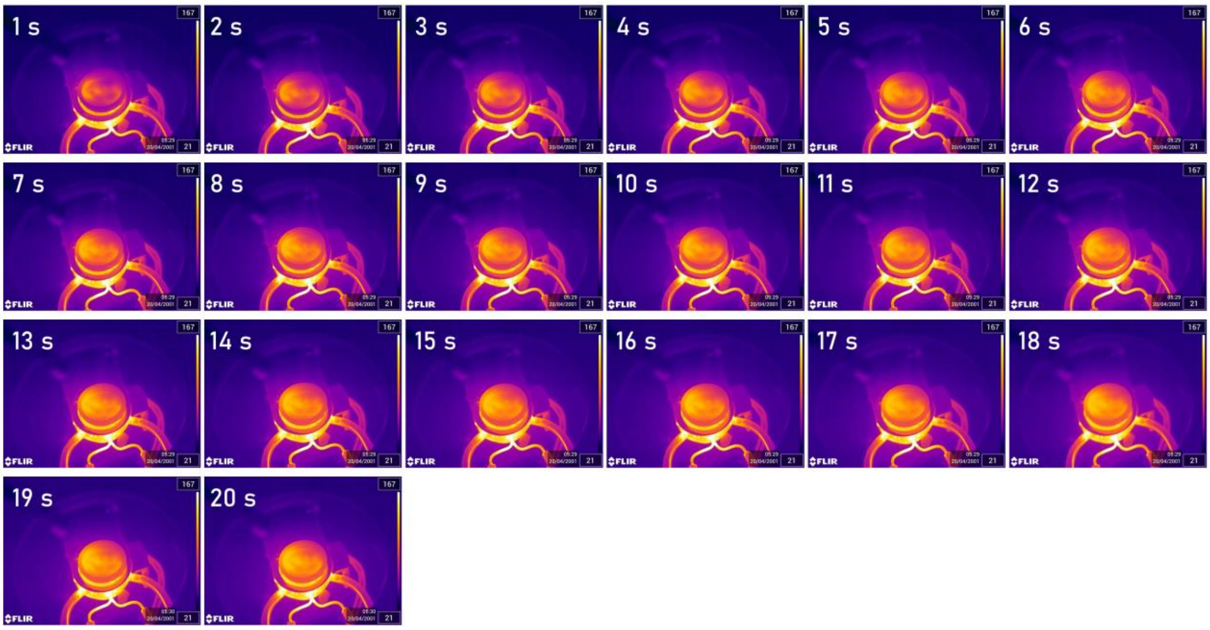

Figure S56. Photos of analyzed luminescence thermometer obtained using thermovision camera in the experimental setup captured as a function of time after placing the quartz holder on the heating stage.

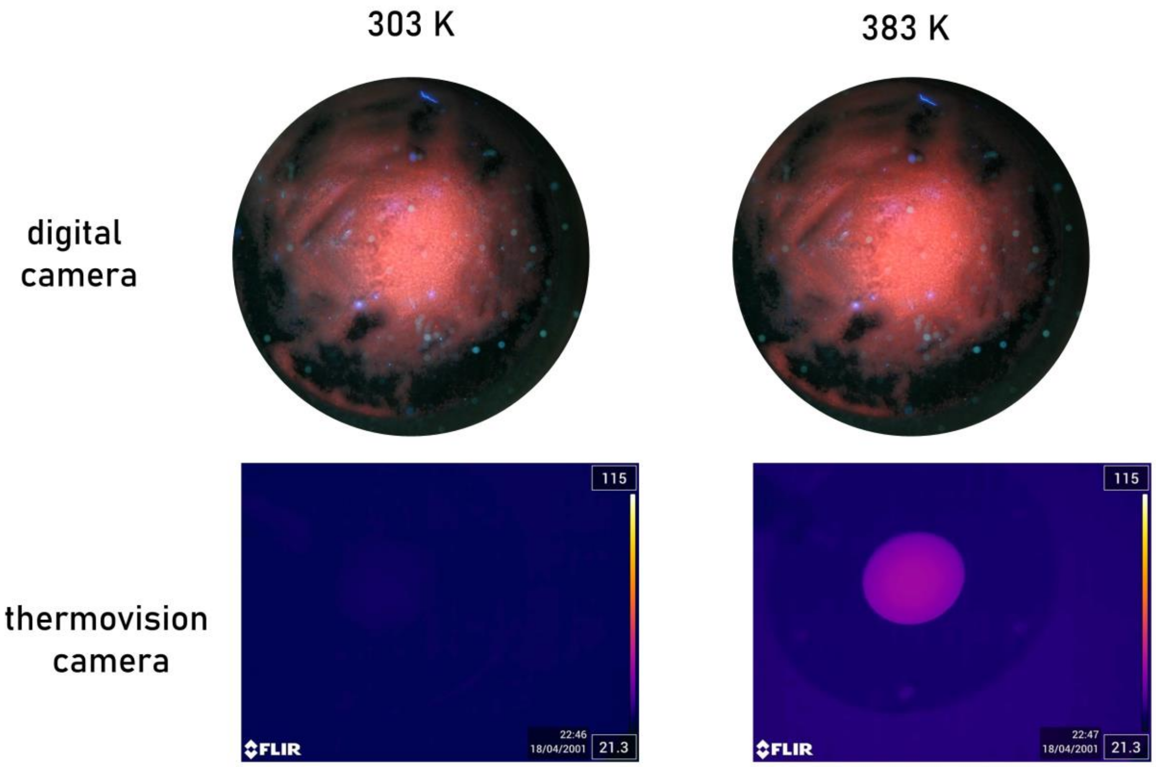

Figure S57. Comparison of the photos of analyzed luminescence thermometer at 303 K (left) and 383 K (right) obtained using digital camera (top) and thermovision camera (bottom) through the quartz optical window of the Linkam THMS600 heating cooling stage.
